# The secreted micropeptide C4orf48 enhances renal fibrosis via an RNA-binding mechanism

**DOI:** 10.1172/JCI178392

**Published:** 2024-04-16

**Authors:** Jiayi Yang, Hongjie Zhuang, Jinhua Li, Ana B. Nunez-Nescolarde, Ning Luo, Huiting Chen, Andy Li, Xinli Qu, Qing Wang, Jinjin Fan, Xiaoyan Bai, Zhiming Ye, Bing Gu, Yue Meng, Xingyuan Zhang, Di Wu, Youyang Sia, Xiaoyun Jiang, Wei Chen, Alexander N. Combes, David J. Nikolic-Paterson, Xueqing Yu

**Affiliations:** 1 Department of Nephrology, The First Affiliated Hospital, Sun Yat-sen University, Guangzhou, China.; 2 Key Laboratory of Nephrology, National Health Commission and Guangdong Province, Guangzhou, China.; 3 Department of Paediatrics, The First Affiliated Hospital, Sun Yat-sen University, Guangzhou, China.; 4 Department of Nephrology and; 5 Guangdong-Hong Kong Joint Laboratory on Immunological and Genetic Kidney Diseases, Guangdong Provincial People’s Hospital and Guangdong Academy of Medical Sciences, Guangzhou, China.; 6 The Second Clinical College, Guangdong Medical University, Dongguan, Guangdong, China.; 7 Department of Nephrology, Monash Health and Department of Medicine and; 8 Department of Anatomy and Developmental Biology, Monash Biomedicine Discovery Institute, Monash University, Clayton, Victoria, Australia.; 9 Department of Clinical Laboratory, Guangdong Provincial People’s Hospital and Guangdong Academy of Medical Sciences, Guangzhou, China.; 10 Department of Biostatistics, UNC Gillings School of Global Public Health, University of North Carolina, Chapel Hill, North Carolina, USA.; 11 School of Life Science, Tsinghua University, Beijing, China.

**Keywords:** Nephrology, Fibrosis

## Abstract

Renal interstitial fibrosis is an important mechanism in the progression of chronic kidney disease (CKD) to end-stage kidney disease. However, we lack specific treatments to slow or halt renal fibrosis. Ribosome profiling identified upregulation of a secreted micropeptide, C4orf48 (Cf48), in mouse diabetic nephropathy. *Cf48* RNA and protein levels were upregulated in tubular epithelial cells in human and experimental CKD. Serum Cf48 levels were increased in human CKD and correlated with loss of kidney function, increasing CKD stage, and the degree of active interstitial fibrosis. Cf48 overexpression in mice accelerated renal fibrosis, while *Cf48* gene deletion or knockdown by antisense oligonucleotides significantly reduced renal fibrosis in CKD models. In vitro, recombinant Cf48 (rCf48) enhanced TGF-β1–induced fibrotic responses in renal fibroblasts and epithelial cells independently of Smad3 phosphorylation. Cellular uptake of Cf48 and its profibrotic response in fibroblasts operated via the transferrin receptor. RNA immunoprecipitation–sequencing identified Cf48 binding to mRNA of genes involved in the fibrotic response, including *Serpine1*, *Acta2*, *Ccn2*, and *Col4a1*. rCf48 binds to the 3′UTR of *Serpine1* and increases mRNA half-life. We identify the secreted Cf48 micropeptide as a potential enhancer of renal fibrosis that operates as an RNA-binding peptide to promote the production of extracellular matrix.

## Abstract 2

The secreted micropeptide C4orf48 is correlated with loss of kidney function and promotes fibrosis in human and experimental chronic kidney disease.

## Graphical abstract

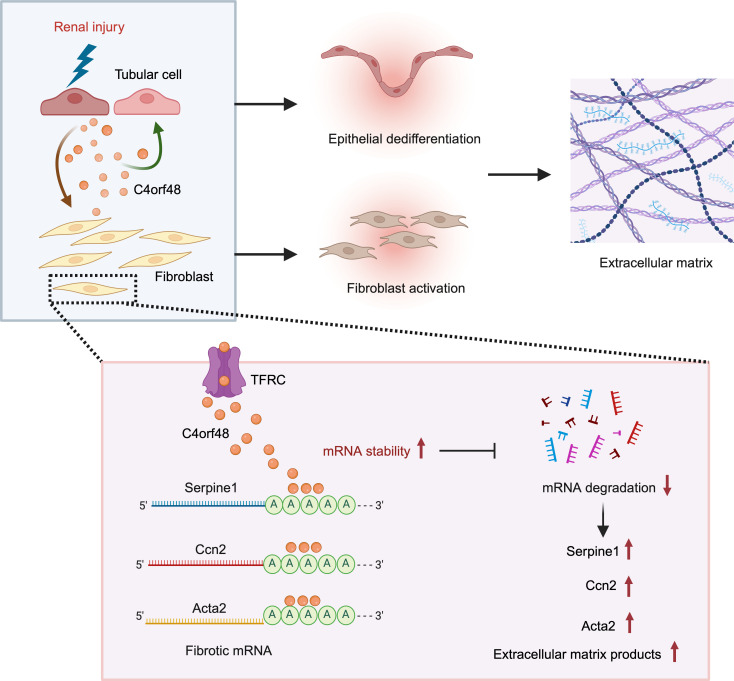

## Introduction

Approximately 8%–18% of adults suffer from chronic kidney disease (CKD) worldwide in both developed and developing countries (1). Diabetes and hypertension are the 2 leading causes of CKD (2). Hyperglycemia, hypertension, and albuminuria management, together with sodium/glucose cotransporter 2 inhibitor administration for type 2 diabetes, retard but do not halt the progression to end-stage renal disease (ESRD) (2, 3). Patients with ESRD require kidney replacement therapies, such as dialysis or kidney transplantation, which have a major impact on patients and their families, as well as a high healthcare costs. This urgent unmet clinical need prompted us to seek new therapeutic targets to suppress renal fibrosis.

Glomerular filtration barrier damage and glomerular hemodynamic changes are characteristic of most CKD forms. However, injury to the proximal tubular epithelium, which makes up 50% of kidney cells, is also an important driver in the progression of CKD. Cortical interstitial expansion is the best histologic predictor of renal functional decline across all CKD types (4–6). Across multiple cohorts of patients with CKD, urinary levels of KIM1 (also known as HAVCR1), which is a specific marker of proximal tubular epithelial cell injury, are highest in patients with reduced kidney function (7). In addition, diabetes-induced proximal tubular cell damage is an early event that both predicts and contributes to the development of diabetic nephropathy (DN) (8, 9). Furthermore, maladaptive repair of damaged proximal tubular cells is a key factor in the transition of acute kidney injury (AKI) to CKD (10). Thus, understanding how proximal tubular cell damage drives the progression to CKD may identify new therapeutic targets to target renal fibrosis.

Micropeptides are small proteins of less than 100 amino acids that are encoded by small open reading frames (smORFs). This class of molecule has been overlooked because smORFs often lack features of classical protein-coding genes, and a threshold length of 300 nucleotides, or 100 amino acids, was arbitrarily and historically set as a minimum size for ontological studies. However, the importance of micropeptides in regulating physiological and/or pathological processes is now being recognized, such as in embryonic development (11), DNA repair (12), metabolic homeostasis (13), muscle regeneration (14), and cell death (15). Nevertheless, the existence and biological function of micropeptides in the pathogenesis of CKD is unknown.

The present study used ribosome profiling and bioinformatics analysis to screen a mouse diabetic kidney disease and identified secreted micropeptide C4orf48 (Cf48 for brevity, also known as NICOL1) as a candidate molecule involved in renal fibrosis. Cf48 expression was upregulated in human and mouse CKD models, with serum Cf48 levels inversely correlated with kidney function in human CKD. Transgenic overexpression of Cf48 or *Cf48* gene deletion in mice substantially modulated renal fibrosis in models of streptozotocin-induced (STZ-induced) DN, folic acid–induced (FA-induced) nephropathy (FAN), and unilateral ureteral obstruction (UUO). Recombinant Cf48 (rCf48) enhanced transforming growth factor β1–induced (TGF-β1–induced) fibrotic responses in renal fibroblasts and epithelial cells independently of Smad3 activation (phosphorylation). rCf48 binds to and is taken up into fibroblasts by the transferrin receptor (TFRC, also known as CD71). Mechanistically, Cf48 binds to a large number of RNA species involved in extracellular matrix deposition. For example, Cf48 binds to the 3′-end of *Serpine1* mRNA, causing an increase in mRNA half-life. These data establish that the Cf48 micropeptide acts as a potential enhancer of renal fibrosis via binding to RNA molecules to promote extracellular matrix deposition. Cf48 may be a biomarker of active renal fibrosis and a therapeutic target in CKD.

## Results

### Identification of Cf48 as a putative profibrotic micropeptide in CKD.

**Figure 1:**
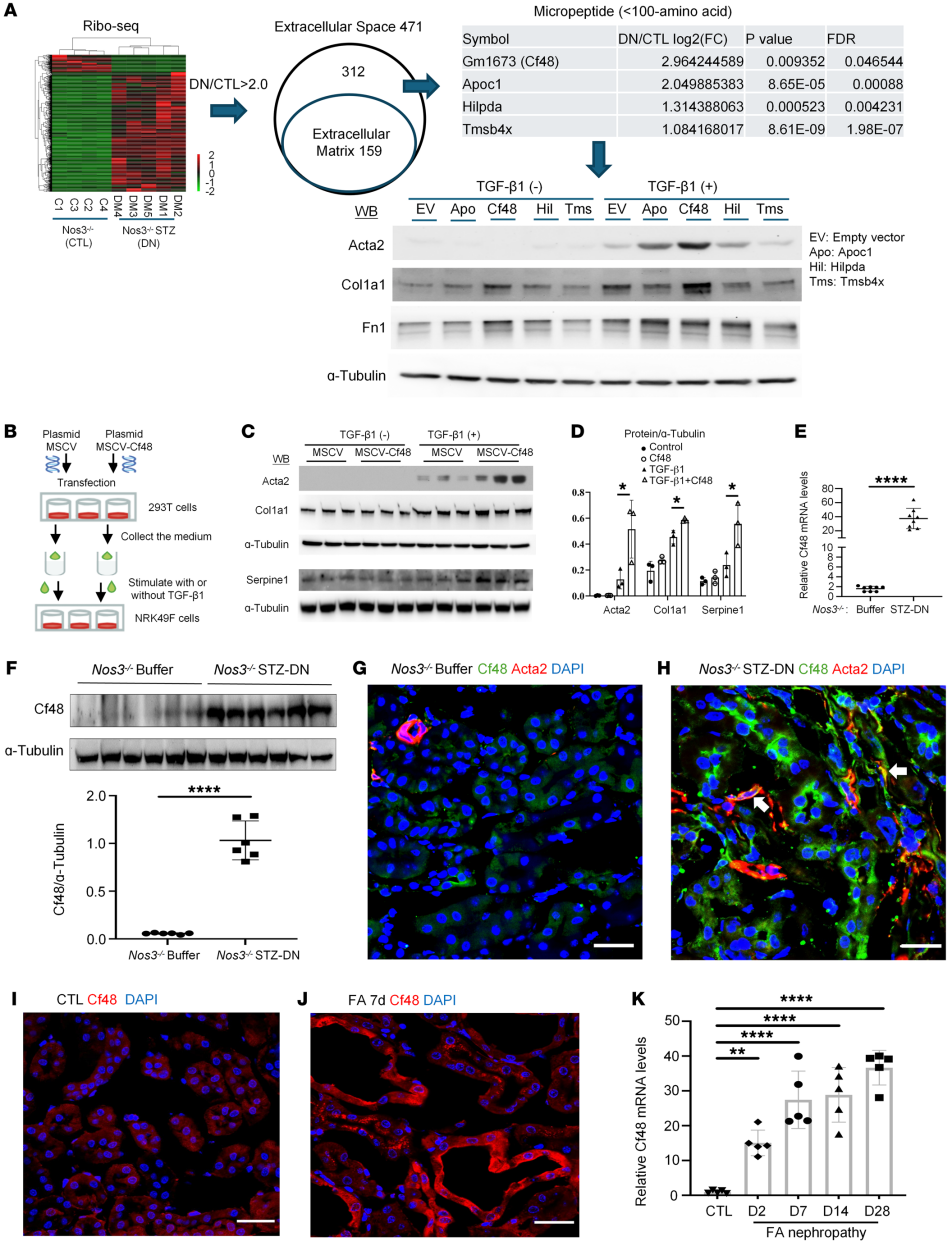
Identification of Gm1673 (C4orf48) as a candidate enhancer of renal fibrosis. (**A**) Flow chart of the screen of kidney tissue from streptozotocin-induced (STZ-induced) diabetic nephropathy (DN) in *Nos3*^−/−^ mice versus age-matched *Nos3*^−/−^ control (CTL) mice. Ribosome-sequencing (Ribo-seq) with bioinformatic analysis identified 471 differentially expressed RNAs as candidate extracellular molecules, including those encoding the following 4 peptides: mouse Gm1673 (C4orf48 in humans, Cf48), Apoc1, Hilpda, and Tmsb4x. Each peptide was overexpressed in NRK49F cells using a retroviral vector, and then cells underwent 48 hours with or without TGF-β1 stimulation and were analyzed for Acta2, collagen I (Col1A1), and fibronectin (Fn1) expression by Western blotting (WB). (**B**–**D**) 293T cells were transfected with an empty vector (MSCV) or Cf48-expressing vector (MSCV-Cf48). Cell-conditioned medium was collected after 48 hours and used to treat NRK49F cells with or without TGF-β1 stimulation for 48 hours. (**C**) WB analysis of Acta2, collagen I, and Serpine1 expression, with quantification (**D**). Data are expressed as mean ± SD. *P < 0.05 by 1-way ANOVA with Tukey’s multiple-comparison test. (**E** and **F**) Analysis of Cf48 expression in kidneys 6 weeks after STZ-induced DN in *Nos3*^−/−^ mice versus control buffer-treated mice showing (**E**) RT-qPCR and (**F**) WB. ****P < 0.0001 by 2-tailed Student’s *t* test. (**G** and **H**) Confocal microscopy of Cf48 (green) and Acta2 (red) staining kidney of control *Nos3^–/–^* (**G**) and *Nos3^–/–^* DN (**H**). Arrows indicate double-labeled Acta2^+^Cf48^+^ cells. (**I**–**K**) Analysis of kidney Cf48 expression on day 7 of folic acid–induced nephropathy (FAN) showing confocal microscopy of Cf48 staining in control (CTL) (**I**) and FAN kidneys (**J**), and RT-PCR analysis of Cf48 RNA expression (**K**). Data are expressed as mean ± SD. **P < 0.01; ****P < 0.0001 by 1-way ANOVA with Tukey’s multiple-comparison test. Scale bars: 50 μm.

Seeking to identify micropeptides involved in renal fibrosis, we used ribosome profiling to screen kidney tissue from *Nos3^−/−^* mice (which lack nitric oxide synthase 3 in endothelial cells) with STZ-induced DN — a model of progressive DN (16) — compared with age-matched, nondiabetic *Nos3^−/−^* control mice (Supplemental Table 1; supplemental material available online with this article; https://doi.org/10.1172/JCI178392DS1). Differentially expressed (*P* < 0.05 by 2-tailed Student’s *t* test) molecules were defined, with a criterion of a 2-fold or greater change between the 2 groups (Supplemental Table 2). Enrichment analysis identified upregulation of 471 molecules with a putative extracellular localization in DN versus control mice (Supplemental Table 3). After excluding 159 extracellular matrix molecules, 15 micropeptides (≤100 amino acids) were identified from 312 potential candidates. Chemokine ligands (Ccl5, Ccl7, Ccl8, Ccl11, Ccl17, Ccl20, Ccl22, Cxcl1, and Cxcl10), and micropeptides without orthologs in humans (Wfdc15b and Wfdc17) were excluded. The remaining 4 peptides were Gm1673 (C4orf48 in humans, Cf48), apolipoprotein C1 (Apoc1), Hilpda, and thymosin β4 (Tmsb4x). To screen for a profibrotic function, these 4 peptides were individually overexpressed in the rat renal fibroblast line NRK49F via a retroviral vector. Cf48 overexpression most clearly enhanced TGF-β1–induced upregulation of Acta2 (α-smooth muscle actin, α-SMA), collagen I, and fibronectin in comparison with the empty vector control (Figure 1A). Therefore, Cf48 was investigated as a potential enhancer of the fibrotic response.

A comparison of the Cf48 amino acid sequence shows high conservation between humans and mice, and substantial homology with more distant genera (Supplemental Figure 1A). A signal peptide is predicted in both human and mouse Cf48 (Supplemental Figure 1, B and C). To test for secretion of a bioactive peptide, 293T cells were transfected with a Cf48 overexpression plasmid, or empty control plasmid, and conditioned media collected. Mass spectrometry (MS) confirmed the presence of Cf48 in Cf48-conditioned media (Supplemental Figure 1D). Conditional media from Cf48-transfected cells substantially enhanced TGF-β1–stimulated expression of Acta2, collagen I, and Serpine1 in NRK49F cells (Figure 1, B–D).

### Upregulation of Cf48 expression in human and experimental CKD.

In contrast with the very low levels of *Cf48* RNA and protein seen in normal mouse kidney, mice with STZ-induced DN showed a very substantial increase in kidney Cf48 RNA and protein levels (Figure 1, E–H). Confocal microscopy showed very little staining for Cf48 in normal mouse kidney, but substantial tubular Cf48 staining was evident in mouse DN, with some Acta2^+^ myofibroblasts also showing Cf48 staining (Figure 1, G and H). Increased kidney Cf48 RNA levels and increased Cf48 protein expression in tubular cells (and some myofibroblasts) was also evident in FAN, a model of AKI-to-CKD transition (Figure 1, I–K).

**Figure 2:**
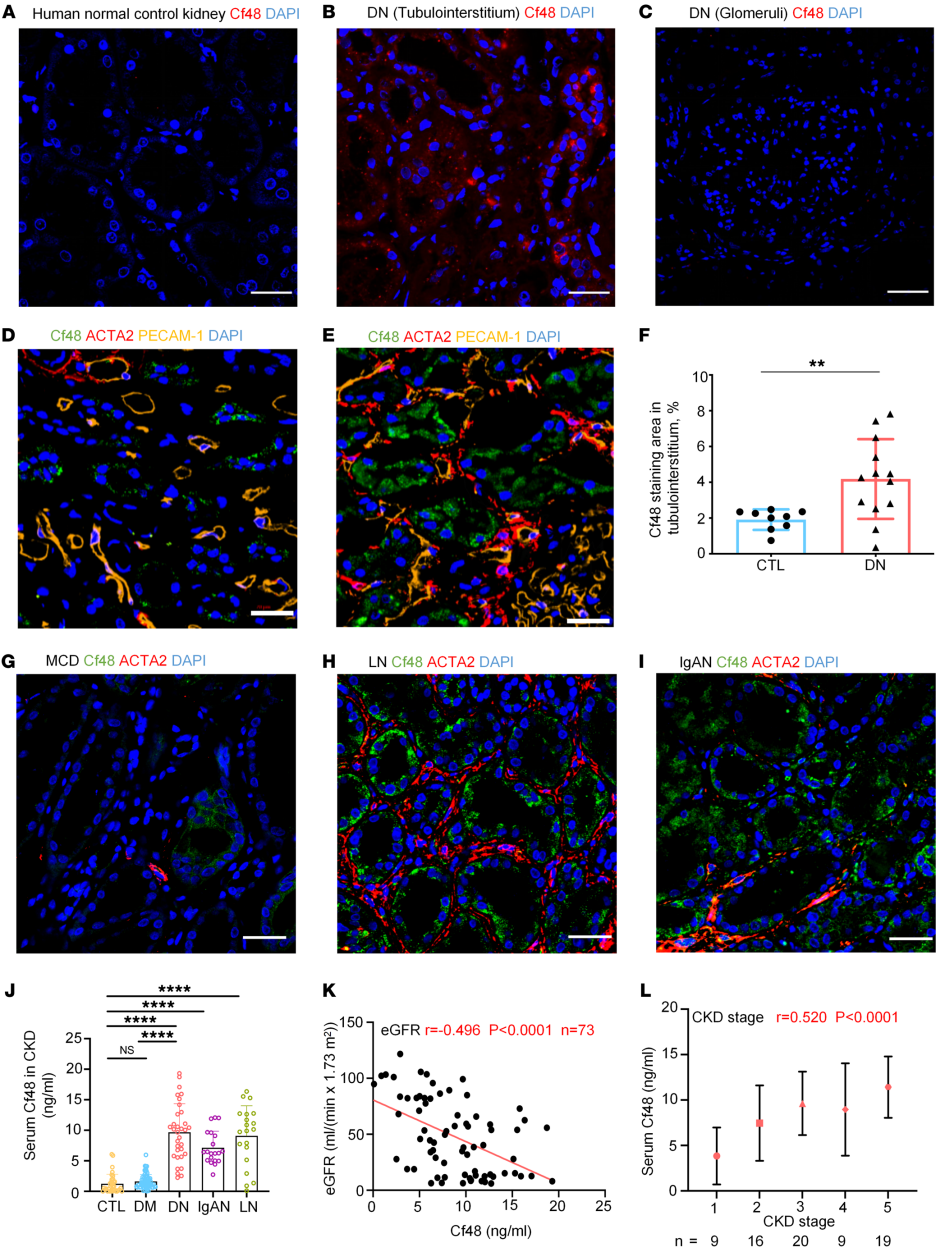
Cf48 expression in human kidney disease. (**A**–**C**) In situ hybridization shows a lack of *Cf48* RNA expression in normal control kidney (**A**), and upregulation of *Cf48* RNA in the tubulointerstitium in diabetic nephropathy (DN) (**B**), but it is virtually absent in the glomerular compartment in DN (**C**). (**D** and **E**) Confocal microscopy staining of Cf48 (green), Acta2 (red), PECAM-1 (CD31, yellow), and DAPI (blue) in normal control kidney (**D**) and DN (**E**). (**F**) Quantitation of the Cf48 staining area (%) in the tubulointerstitium in normal control (CTL) and DN. **P < 0.01 by 2-tailed Student’s *t* test. (**G**–**I**) Confocal microscopy staining of Cf48 (green), Acta2 (red), and DAPI (blue) in minimal change disease (MCD) (**G**), lupus nephritis (LN) (**H**), and IgA nephropathy (IgAN) (**I**). (**J**) Serum Cf48 levels in normal healthy controls (CTL), diabetes mellitus without kidney disease (DM), DN, IgAN, and LN. Data are expressed as mean ± SD. ****P < 0.0001 by 1-way ANOVA with Tukey’s multiple-comparison test. NS, not significant. (**K**) Spearman’s correlation between serum Cf48 levels and estimated glomerular filtration rate (eGFR) in DN, LN, and IgAN groups. (**L**) Spearman’s correlation between serum Cf48 levels and CKD stage in DN, LN, and IgAN groups. Scale bars: 50 μm.

In situ hybridization revealed very low *Cf48* mRNA levels in normal human kidney, but *Cf48* mRNA was markedly increased in tubular epithelial cells, but not glomerular cells, in human DN (Figure 2, A–C). Immunofluorescent staining showed marked Cf48 protein expression in tubular epithelial cells in DN, IgA nephropathy (IgAN), and lupus nephritis (LN), with little to no expression in normal human kidneys or in minimal change disease (Figure 2, D–I). In contrast with the high percentage of Cf48-positive cells in tubulointerstitum, a very low percentage of Cf48-positive cells in glomeruli was observed in human DN (Supplemental Figure 2), albeit no mRNA was detectable in the glomerular compartment by in situ hybridization (Figure 2C). A similar expression pattern in glomeruli could be seen in mouse STZ-induced DN in *Nos3^−/−^* mice (Supplemental Figure 3).

The pattern of Cf48 expression was compared to markers of the thick ascending limb (Umod and NKCC2), proximal tubules (*Lotus* *tetragonolobus* lectin, LTL), and damaged/dedifferentiated tubules (VCAM1) (17–19). In normal human and mouse kidney, Cf48 is primarily expressed in the thick ascending limb, as shown by colocalization with Umod and NKCC2, and is largely absent from LTL^+^ proximal tubules (Supplemental Figures 4 and 5). In human DN, Cf48 expression is evident in the thick ascending limb, but is also seen in VCAM1^+^ dedifferentiated tubules, with Cf48 staining also seen in some dilated proximal tubule cells that exhibited relatively weak LTL staining (Supplemental Figure 4). Similarly, in mouse CKD models, Cf48 expression is still seen in the thick ascending limb cells, but the main increase in Cf48 expression is evident in VCAM1^+^ dedifferentiated tubular cells, with little Cf48 expression seen in healthy-looking proximal tubular cells with strong luminal LTL staining (Supplemental Figures 5–7).

### Serum Cf48 levels correlate with loss of renal function in human CKD and as a potential biomarker of active fibrogenesis in a mouse model of CKD.

**Figure 3:**
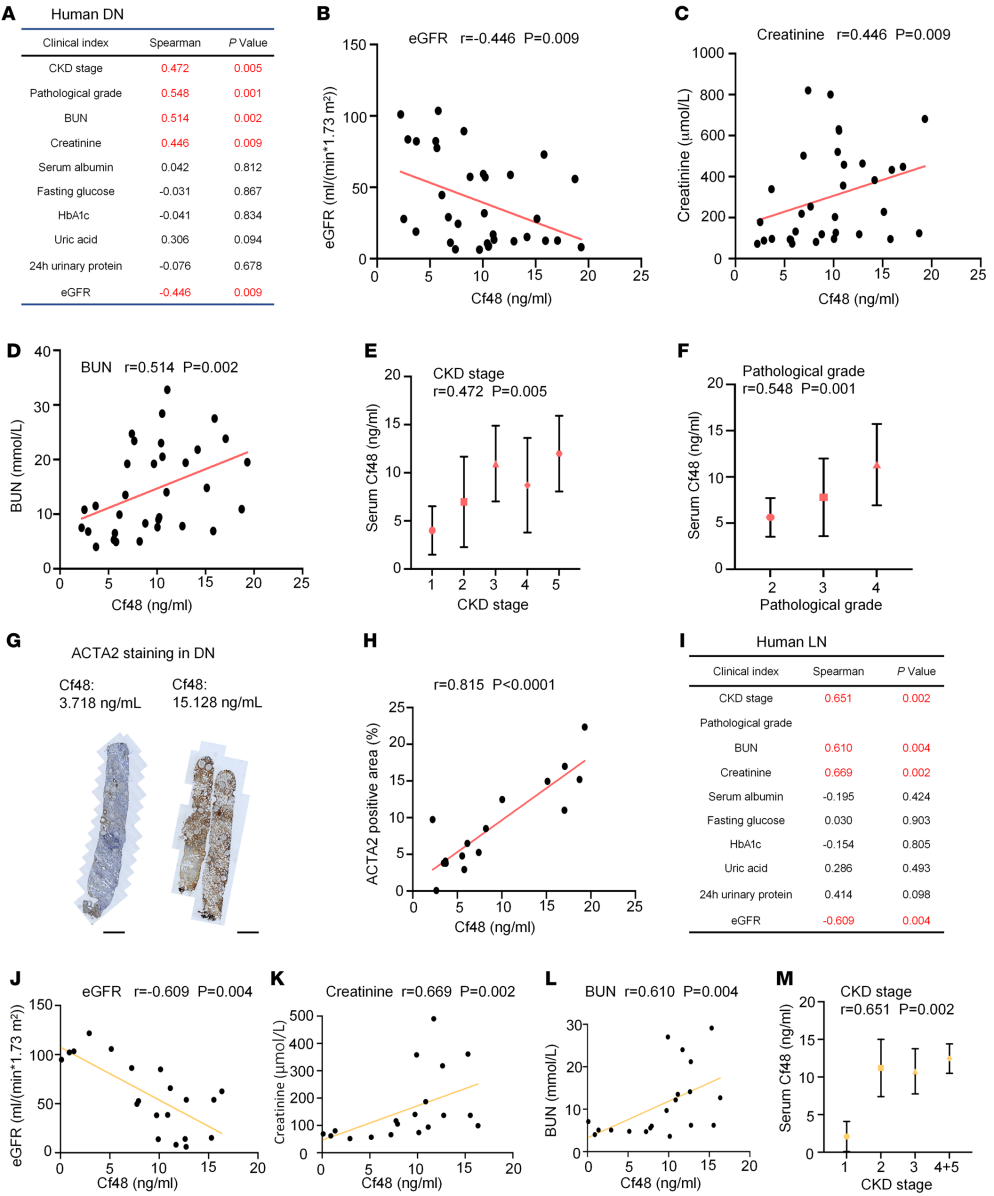
Correlation of serum Cf48 levels with clinical and pathological parameters in human CKD. (**A**–**H**) Analysis of human diabetic nephropathy (DN) (*n* = 33). (**A**) Correlation table of serum Cf48 levels and various clinical indices. Graphs show correlations between serum Cf48 levels and estimated glomerular filtration rate (eGFR) (**B**), serum creatinine levels (**C**), blood urea nitrogen (BUN) levels (**D**), CKD stage (**E**), and pathological grade (**F**). (**G**) Examples of Acta2 immunostaining in DN cases with low or high serum Cf48 levels. (**H**) Correlation of serum Cf48 levels with interstitial area of Acta2 staining. (**I**–**M**) Analysis of human lupus nephritis (LN) (*n* = 20). (**I**) Correlation table of serum Cf48 levels and various clinical indices. Graphs show correlations between serum Cf48 levels and eGFR (**J**), serum creatinine levels (**K**), BUN levels (**L**), and CKD stage (**M**). Scale bars: 1 mm.

Given the prediction of Cf48 as a secreted micropeptide (Supplemental Figure 1, B and C), we examined serum Cf48 levels in patients with CKD. Low levels of Cf48 were detected in the serum of healthy controls (median 0.6750 ng/mL, IQR: 0.1463–1.6760) and diabetes mellitus without kidney disease (DM) groups (median 1.3420 ng/mL, IQR: 0.7780–2.1640). A 7- to 10-fold increase in Cf48 serum levels was evident in the DN, IgAN, and LN groups (Figure 2J and Supplemental Table 4). Analysis of the combined DN, LN, and IgAN groups showed a negative correlation between serum Cf48 levels and renal function (estimated glomerular filtration rate, eGFR), and a positive correlation with CKD stage (Figure 2, K and L). This significant correlation between serum Cf48 levels and eGFR remained after multivariate analysis to adjust for potential confounding factors such as age, sex, and comorbidities (Supplemental Table 5). Further analysis was performed in each disease group. In DN patients (*n* = 33), serum Cf48 levels correlated with reduced renal function (eGFR, serum creatinine, and blood urea nitrogen [BUN] levels) and CKD stage, but no correlation was evident with fasting blood glucose, hemoglobin A1c (HbA1c), 24-hour urinary protein excretion, or serum uric acid levels (Figure 3, A–E). In the DN group, serum Cf48 levels also correlated with the pathological grade of disease and with the area of interstitial Acta2 staining (myofibroblast accumulation, indicating active fibrosis) (Figure 3, F–H). In the LN group (*n* = 20), serum Cf48 levels correlated with loss of renal function (eGFR, serum creatinine, and BUN) and with CKD stage and pathological grade (Figure 3, I–M). In the IgAN group (*n* = 20), serum Cf48 levels correlated only with the pathological grade of disease (Supplemental Figure 8). Independent confirmation of our results was obtained from CKD studies in the NephroSeq database (https://www.nephroseq.org/resource/login.html Accessed July 6, 2023.) (20, 21). Increased *Cf48* RNA levels in the tubulointerstitium correlated with a reduction in GFR in a European multicenter study and in a North American study (Supplemental Figures 9 and 10) (20, 21). In sum, these data suggest that Cf48 levels in both kidney and serum are associated with CKD stage.

To further clarify the relationship between serum Cf48 levels, renal function, and fibrosis, we performed a sham or 15-minute renal bilateral ischemia operation in C57BL/6J mice, a model of resolving acute renal failure. No difference in serum creatinine levels were evident on day 28 between ischemia-reperfusion injury (IRI) and sham groups — indicating recovery of renal function after IRI. However, mice that underwent IRI displayed marked renal fibrosis compared with the sham controls, with a significant increase in staining for fibronectin, collagen IV, and Acta2 in myofibroblasts. Notably, serum Cf48 levels were substantially increased in the IRI group, but not in the sham group (Supplemental Figure 11). Our data provide evidence that increased Cf48 levels are associated with renal fibrosis and are not dependent on renal function, and that serum Cf48 levels may be a potential biomarker of active fibrogenesis.

### Cf48 overexpression enhances renal fibrosis in mouse CKD models.

**Figure 4:**
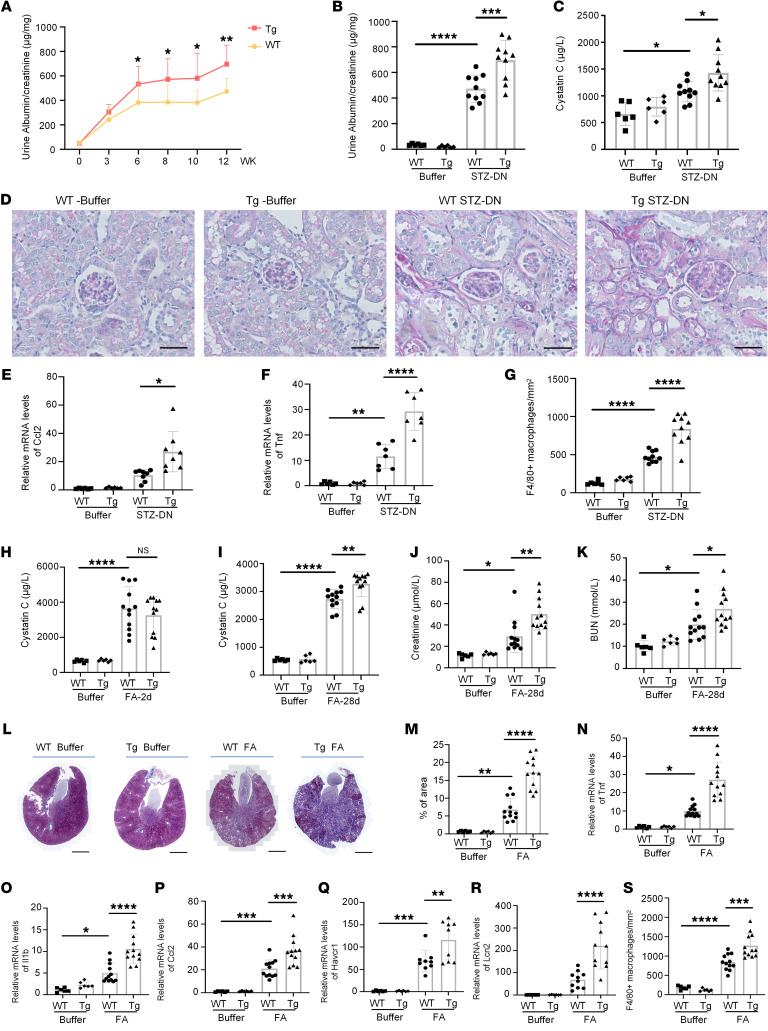
Cf48 overexpression enhances renal fibrosis and inflammation in mouse CKD models. (**A**–**G**) A 12-week streptozotoxin-induced (STZ-induced) DN model in WT and *Cf48*-transgenic (Tg) mice, with buffer injected mice as nondiabetic controls. (**A**) Time course of the urine albumin/creatinine ratio. *P < 0.05; **P < 0.01 by 2-tailed Student’s *t* test. (**B**) Urine albumin/creatinine ratio at week 12. (**C**) Serum cystatin C levels. (**D**) PAS staining in 12-week STZ-DN or buffer-treated WT or *Cf48*-Tg mouse kidneys. Scale bars: 50 μm. (**E** and **F**) RT-qPCR analysis of *Ccl2* (**E**) and *Tnf* (**F**) mRNA levels. (**G**) Number of F4/80^+^ macrophages scored from immunostaining. (**H**–**S**) Day 2 and 28 folic acid nephropathy (FAN) in WT and *Cf48*-Tg mice, with buffer-injected mice as controls. (**H**) Serum cystatin C levels on day 2 of FAN. (**I**–**K**) Serum levels of (**I**) cystatin C, (**J**) creatinine, and (**K**) blood urea nitrogen (BUN), on day 28 of FAN. (**L** and **M**) Representative whole-kidney cross sections (**L**) and quantification (**M**) of interstitial collagen deposition in Masson’s trichrome–stained kidneys on day 28 of FAN. Scale bars: 1 mm. (**N**–**R**) RT-qPCR analysis of kidney mRNA levels of (**N**) *Tnf*, (**O**) *Il1b*, (**P**) *Ccl2*, (**Q**) *Kim1/Havcr1*, and (**R**) *Ngal/Lcn2*, on day 28 of FAN. (**S**) Number of F4/80^+^ macrophages scored from immunostaining. Data are expressed as mean ± SD. *P < 0.05; **P < 0.01; ***P < 0.001; ****P < 0.0001 by 1-way ANOVA with Tukey’s multiple-comparison test.

Transgenic (Tg) mice were created in which the *Cf48* open reading frame was driven by the CAG promoter in all cells following doxycycline treatment. Wild-type (WT) and *Cf48*-Tg (Tg) mice given saline injections (controls) showed normal kidney function (serum cystatin C levels), urinary albumin excretion, and kidney histology (Figure 4, A–D); control Tg mice showed higher levels of kidney *Cf48* mRNA compared with control WT mice. Following induction of diabetes, *Cf48* mRNA levels were increased in both WT and Tg mice, although Cf48 expression was higher in Tg-DN compared with WT-DN mice (Supplemental Figure 12A). Type 1 diabetes was induced by low-dose STZ injections, with an equivalent increase in fasting blood glucose levels and plasma HbA1c levels evident in WT and Tg mice (Supplemental Figure 12, B and C). Tg-DN mice showed enhanced albuminuria and elevated serum cystatin C levels compared with WT-DN mice (Figure 4, A–C). PAS staining showed glomerular matrix expansion and basement membrane thickening in some tubules in WT-DN mice, both of which were more prominent in Tg-DN (Figure 4D). Immunofluorescent staining showed increased collagen IV deposition in the glomerulus and tubulointerstitium of Tg-DN mice compared with WT-DN mice (Supplemental Figure 12, D and E). A greater increase in fibronectin deposition and interstitial accumulation of Acta2^+^ myofibroblasts was also evident in Tg-DN versus WT-DN mice (Supplemental Figure 12, D, F, and G). Inflammation was enhanced in Tg-DN versus WT-DN mice based on monocyte chemoattractant protein-1 (*Mcp1*, also known as *Ccl2*) and tumor necrosis factor (*Tnf*) mRNA levels, and infiltration of F4/80^+^ macrophages (Figure 4, E–G).

We also investigated FAN in Tg mice, as a model of AKI-CKD transition (22). Acute renal failure (increased serum cystatin C levels) was not different between WT and Tg mice on day 2 of FAN, indicating that Cf48 overexpression did not affect AKI in this model (Figure 4H). While renal function partially recovered by day 28 of FAN in WT mice, renal function was significantly worse in Tg versus WT mice on day 28 of FAN based on serum cystatin C, serum creatinine, and BUN levels (Figure 4, I–K). Masson’s trichrome staining showed substantial renal interstitial fibrosis on day 28 of FAN in WT mice, which was significantly increased in Tg mice (Figure 4, L and M). Immunofluorescent staining showed a significant increase in the deposition of collagen IV and fibronectin and increased accumulation of Acta2^+^ myofibroblasts in day 28 FAN Tg mice compared with day 28 FAN WT mice (Supplemental Figure 12, H–K). Day 28 FAN Tg mice also showed enhanced mRNA levels of proinflammatory cytokines (*Tnf*, *Il1b*, and *Ccl2*), increased mRNA levels of tubular epithelial cell injury markers (*Havcr1* and *Lcn2*), and infiltration of F4/80^+^ macrophages (Figure 4, N–S). In sum, data from the DN and FAN models demonstrate that Cf48 overexpression enhanced renal fibrosis and inflammation in mouse CKD.

### Cf48 deletion suppresses renal fibrosis in mouse CKD models.

**Figure 5:**
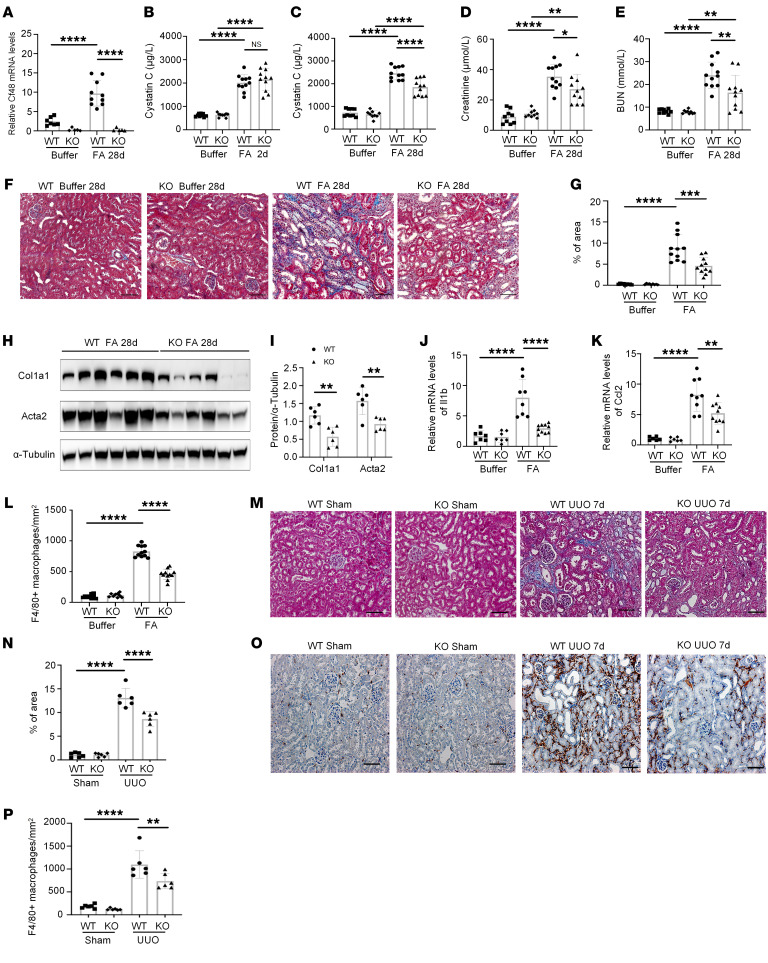
Cf48 deficiency suppresses renal fibrosis in mouse CKD models. Day 2 and 28 folic acid nephropathy (FAN) in WT and *Cf48*-KO mice, with buffer-injected mice as controls. (**A**) Kidney *Cf48* mRNA levels on day 28 of FAN or buffer control. (**B**) Serum cystatin C levels on day 2 of FAN. (**C**–**E**) Serum levels of (**C**) cystatin C, (**D**) creatinine, and (**E**) blood urea nitrogen (BUN), on day 28 of FAN. (**F**) Masson’s trichrome staining of kidney sections on day 28 of FAN, with (**G**) quantification of interstitial collagen deposition. (**H**) Western blot of kidney lysates for collagen I (Col1A1) and Acta2 on day 28 of FAN, with (**I**) quantification of bands compared to the α-tubulin control. (**J** and **K**) RT-qPCR analysis of kidney mRNA levels of (**J**) *Il1b* and (**K**) *Ccl2* on day 28 of FAN. (**L**) Quantification of F4/80^+^ cells in the day 28 FAN model. WT and *Cf48*-KO mice were also compared in a day 7 unilateral ureteral obstruction (UUO) model with sham surgery controls. (**M**) Masson’s trichrome staining of day 7 UUO and sham controls. (**N**) Quantification of Masson’s trichrome staining of interstitial collagen. (**O**) F4/80 immunostaining in day 7 UUO and sham controls. (**P**) Quantification of F4/80^+^ cells in the day 7 UUO model. Data are expressed as mean ± SD. *P < 0.05; **P < 0.01; ***P < 0.001; ****P < 0.0001 by 1-way ANOVA with Tukey’s multiple-comparison test: NS, not significant. Scale bars: 50 μm.

Given that overexpression of Cf48 enhances renal fibrosis, we sought to determine whether *Cf48* gene deletion would protect against fibrosis. Mice deficient in the *Cf48* open reading frame (*Cf48*-KO mice) were generated. *Cf48*-KO mice are healthy and viable, with no obvious abnormalities except for male homozygous *Cf48*-KO mice being infertile. WT and *Cf48*-KO mice were compared in the FAN model, with buffer-injected mice serving as controls. Control KO mice had no detectable *Cf48* mRNA in the kidney (Figure 5A), and both control WT and KO mice showed normal renal function and structure (Figure 5, B–G). Acute renal failure (increased serum cystatin C levels) was not different between WT and KO mice on day 2 of FAN (Figure 5B). WT mice showed increased kidney *Cf48* mRNA and protein levels on day 28 of FAN; however, no Cf48 mRNA or protein was detected in KO kidneys on day 28 of FAN (Figure 5A and Supplemental Figure 13A). *Cf48*-KO mice showed improved renal function on day 28 of FAN compared with WT mice based on serum cystatin C, creatinine, and BUN levels (Figure 5, C–E). A significant reduction in renal fibrosis in KO versus WT mice on day 28 of FAN was evident based on Masson’s trichrome staining, Western blotting for collagen I and Acta2, and immunofluorescent staining for collagen IV, Acta2, and fibronectin (Figure 5, F–I, and Supplemental Figure 13, B–E). In addition, a significant reduction in renal inflammation was seen in KO versus WT mice on day 28 of FAN based on reduced kidney mRNA levels of *Il1b* and *Ccl2* (Figure 5, J and K), and reduced infiltration of F4/80^+^ macrophages (Figure 5L).

Next, we tested *Cf48*-KO mice in a surgically induced model of UUO in which mechanical pressure induces aggressive renal fibrosis (23). Masson’s trichome, immunohistochemistry, and confocal microscopy demonstrated that Cf48 deficiency decreased renal tubulointerstitial fibrosis and inflammation (Figure 5, M and N, and Supplemental Figure 13, F–I). A substantial reduction in the F4/80^+^ macrophage infiltration was also evident in KO mice on day 7 of UUO (Figure 5, O and P). Thus, genetic approaches to overexpress or delete Cf48 establish that Cf48 regulates renal fibrosis and inflammation in 3 mouse CKD models of different etiologies.

### Cf48 antisense oligonucleotide treatment reduces renal fibrosis in mouse CKD models.

**Figure 6:**
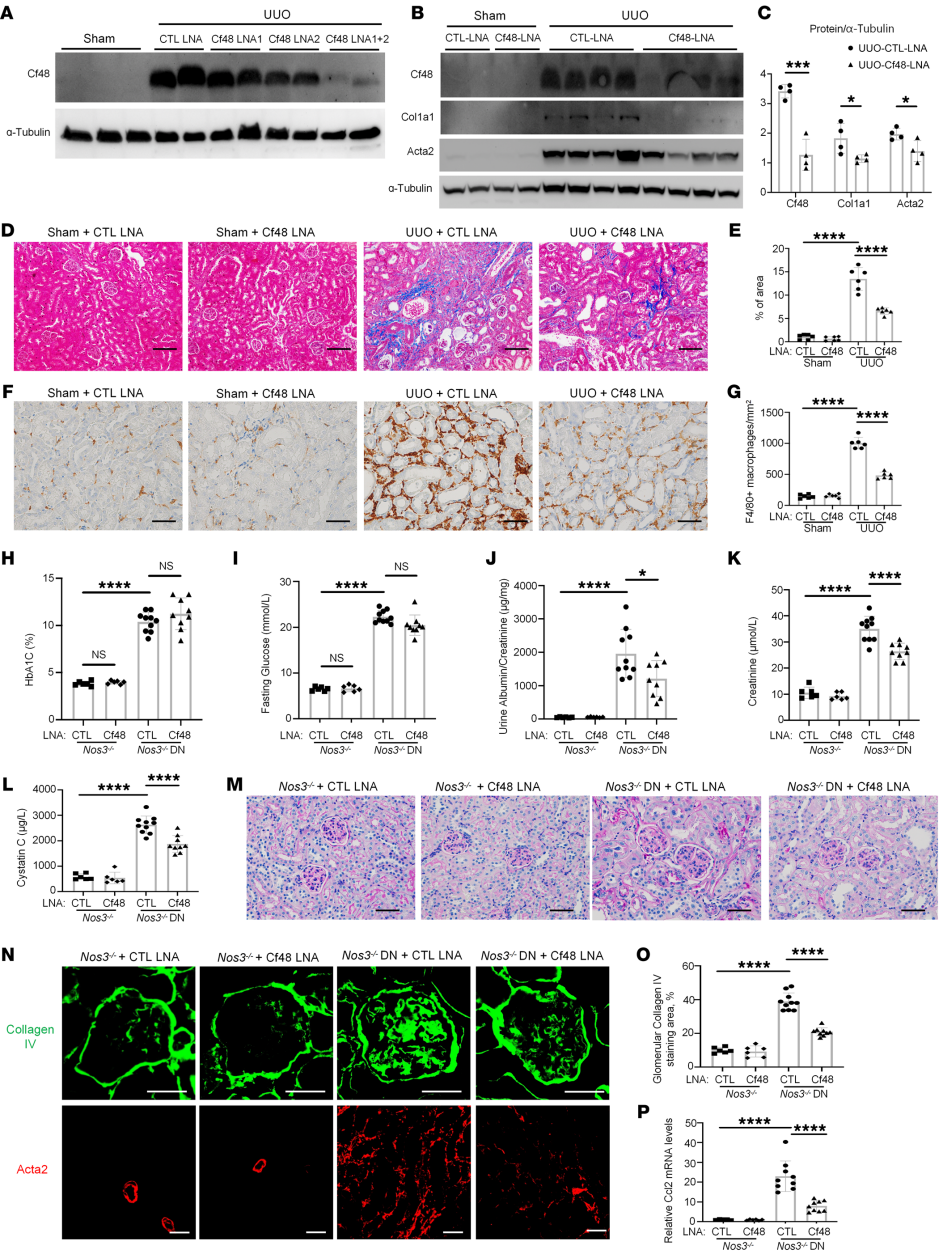
Cf48 LNA administration reduces renal fibrosis and inflammation in mouse CKD models. (**A**) Mice received 10 mg/kg control LNA (CTL LNA), 10 mg/kg Cf48 LNA1, 10 mg/kg Cf48 LNA2, or 5 mg/kg Cf48 LNA1 + 5 mg/kg Cf48 LNA2 combined treatment on days 1 and 4 after completing UUO surgery, with mice euthanized on day 7. Sham-operated mice were controls. Western blot of Cf48 expression in day 7 UUO and sham groups. (**B**–**G**) Day 7 UUO with 5 mg/kg Cf48 LNA1 + 5 mg/kg Cf48 LNA2 (termed Cf48 LNA) or CTL LNA treatment, with sham controls. (**B**) Western blot of kidney Cf48, collagen I (Col1A1), and Acta2 expression, with quantification of blots (**C**). (**D**) Masson’s trichrome staining, with quantification of interstitial collagen staining (**E**). (**F**) Immunostaining of F4/80^+^ macrophages, with quantification (**G**). (**H**–**P**) *Nos3*^–/–^ mice were treated weekly with Cf48 LNA or CTL LNA from 3 weeks after STZ (or buffer administration) until being euthanized on week 8. (**H**) Glycated hemoglobin A1c levels (HbA1c). (**I**) Fasting blood glucose levels. (**J**) Urinary albumin-to-creatinine levels. (**K**) Serum creatinine levels. (**L**) Serum cystatin C levels. (**M**) PAS staining of kidney sections. (**N**) Immunofluorescent staining of collagen IV and Acta2. (**O**) Quantification of percentages of glomerular collagen IV staining. (**P**) RT-qPCR analysis of kidney *Ccl2* mRNA levels. Data are expressed as mean ± SD. *P < 0.05; ***P < 0.001; ****P < 0.0001 by 1-way ANOVA with Tukey’s multiple-comparisons test. NS, not significant. Scale bars: 100 μm (**D**), 50 μm (**F** and **M**), and 25 μm (**N**).

Cf48 antisense locked nucleic acid (LNA) oligonucleotides were tested as a therapeutic strategy to reduce renal fibrosis. LNA refers to an RNA oligonucleotide that has been modified to create an additional bridge connecting the 2′ oxygen and the 4′ carbon of the ribose ring, making it resistant to enzymatic degradation and improving its specificity and affinity as an oligonucleotide in vivo (24). Additionally, LNA oligonucleotides are preferentially taken up by the kidney after administration (25). First, we tested 2 different Cf48 LNAs in the UUO model. Mice received intraperitoneal injections of Cf48 LNA1, Cf48 LNA2, Cf48 LNA1 plus LNA2, or control LNA (CTL LNA) on days 1 and 4 after UUO, with mice euthanized on day 7. Western blotting showed a strong upregulation of Cf48 protein levels in the CTL LNA–treated UUO compared with the sham surgery group (Figure 6A). As single treatments, Cf48 LNA1 and LNA2 produced only minor reductions in Cf48 protein expression; however, the combination of Cf48 LNA1 plus LNA2 was highly effective in knocking down Cf48 expression (Figure 6A). Thus, subsequent experiments used Cf48 LNA1 plus LNA2 (referred to hereafter as Cf48 LNA), which produced a substantial reduction in renal fibrosis as shown by Western blotting for collagen I and Acta2 (Figure 6, B and C), and collagen deposition based on Masson’s trichrome staining (Figure 6, D and E). In addition, Cf48 LNA treatment significantly reduced infiltration of F4/80^+^ macrophages (Figure 6, F and G).

We tested Cf48 LNA as an early intervention treatment in DN using the STZ-DN model in susceptible *Nos3^−/−^* mice. Diabetic *Nos3^−/−^* mice were treated with Cf48 LNA or CTL LNA given once weekly from week 3 until being euthanized at week 8. Treatment with Cf48 LNA did not affect fasting blood glucose or HbA1C levels (Figure 6, H and I). However, Cf48 LNA treatment significantly decreased albuminuria and protected against loss of renal function (Figure 6, J–L). Furthermore, Cf48 LNA treatment significantly reduced glomerular matrix expansion as indicated by PAS staining (Figure 6M), and confirmed by immunostaining (Figure 6N) and quantification of the area of glomerular collagen IV deposition (Figure 6O). Interstitial accumulation of Acta2^+^ myofibroblasts was also reduced by Cf48 LNA treatment (Figure 6N), in concert with a reduction in inflammation as indicated by *Ccl2* mRNA levels (Figure 6P). Collectively, these data demonstrate that Cf48 LNA administration inhibits endogenous Cf48 expression in the kidney, reduces renal fibrosis in the aggressive UUO model, and retards the progression of established DKD.

### Cf48 enhances the TGF-β1–induced fibrotic response via the TFRC.

Given that tubular epithelial cells are the main site of Cf48 expression in human and mouse kidney disease, we investigated whether factors implicated in development of CKD induced tubular Cf48 production. Stimulation of human tubular epithelial cells (HK2) with TGF-β1, TNF, angiotensin II, or cobalt chloride (CoCl_2_) all increased expression of the Cf48 peptide (Supplemental Figure 14), suggesting that a profibrotic, proinflammatory, overactivated renin-angiotensin system and hypoxic conditions may induce expression of Cf48 in renal tubular epithelial cells.

**Figure 7:**
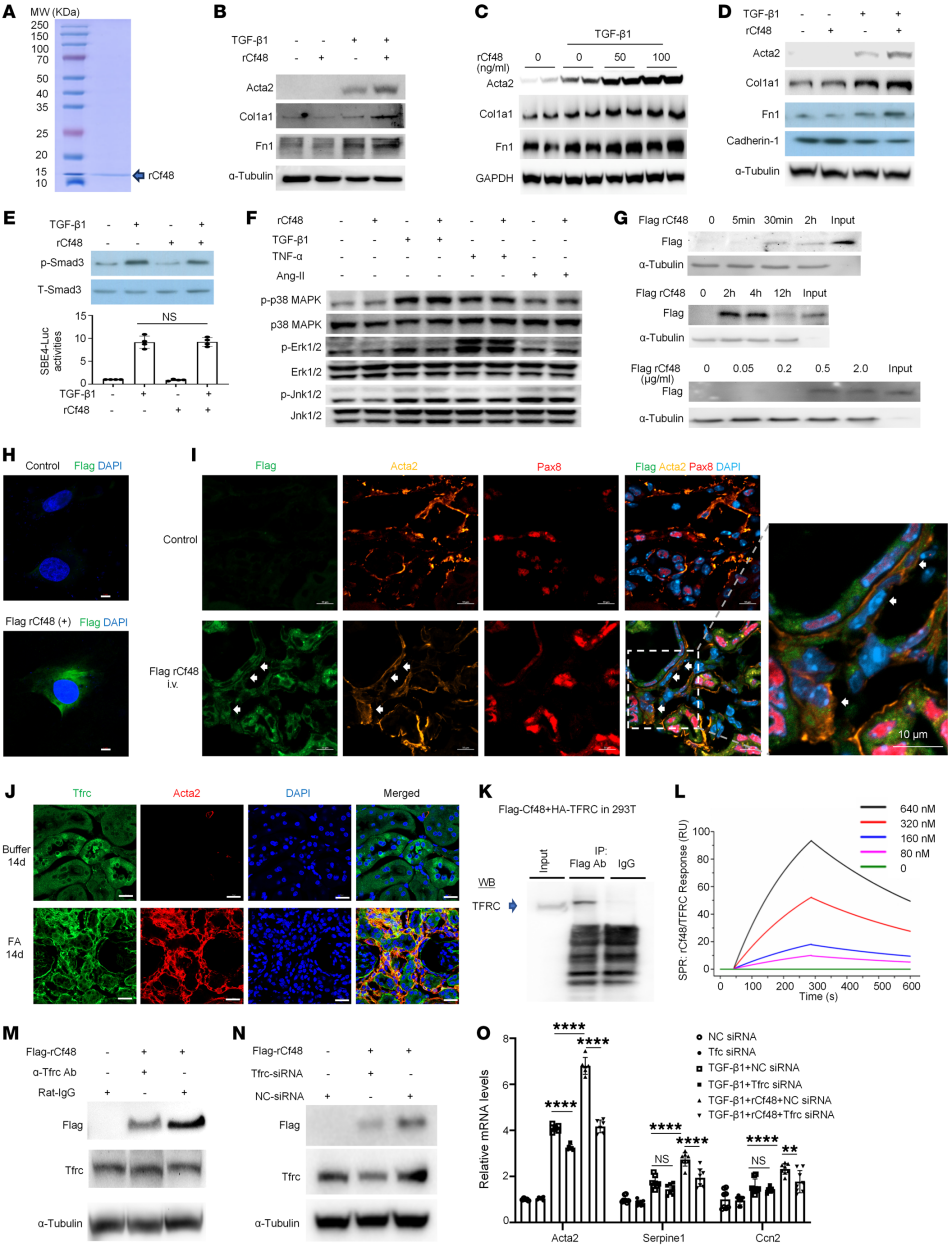
Cf48 enhances the TGF-β1–induced fibrotic response via the transferrin receptor (TFRC). (**A**) Characterization of recombinant mouse Cf48 (rCf48) lacking the signal peptide (i.e., secreted form) via SDS-PAGE. Stimulation of NRK49F cells (**B** and **C**) or NRK52E cells (**D**) with rCf48 with or without TGF-β1 for 48 hours. Western blots (WBs) show Acta2, collagen I (Col1A1), fibronectin (Fn1), and cadherin-1 expression. (**E**) Upper panel: NRK49F cells were stimulated for 30 minutes with rCf48 with or without TGF-β1 and expression of total Smad3 (T-Smad3) and C-terminal phosphorylation of Smad3 (p-Smad3) shown by WB. Lower panel: SBE4-luciferase activity assessed in 293T cells 15 hours after stimulation with rCf48 with or without TGF-β1. (**F**) WB analysis of phosphorylation of p38 MAPK, ERK1/2, and JNK1/2 30 minutes after stimulation of NRK49F cells with rCf48 with or without TGF-β1, TNF-α, or angiotensin II (Ang-II). (**G**) NRK49F cells were cultured with FLAG-tagged rCf48 for different times (top 2 blots), or with different doses of FLAG-tagged rCf48 for 2 hours (bottom blot). Uptake of FLAG-tagged rCf48 was assessed by WB. (**H**) Confocal microscopy shows uptake of FLAG-tagged rCf48 (green) 2 hours after addition to NRK49F cells. Scale bars: 5 μm. (**I**) Confocal microscopy of mouse kidney 5 minutes after tail vein injection of 200 μg of FLAG-tagged rCf48 (green) on day 14 of folic acid–induced neuropathy (FAN). Arrows indicate myofibroblasts double stained for FLAG and Acta2 (yellow). Scale bars: 10 μm. (**J**) Confocal microscopy showing TFRC expression in renal tubular epithelial cells and myofibroblasts in buffer control mice and on day 14 of FAN. Scale bars: 20 μm. (**K**) Immunoprecipitation/WB shows an interaction between FLAG-tagged Cf48 and HA-tagged TFRC after both were overexpressed in 293T cells. (**L**) Surface plasmon resonance (SPR) shows an interaction between rCf48 and recombinant TFRC. (**M** and **N**) WB shows that uptake of FLAG-tagged rCf48 after a 2-hour incubation with NRK49F cells can be substantially reduced by pretreatment with a neutralizing anti-TFRC antibody (versus rat IgG control) (**M**), or by pretreatment with *Tfrc* siRNA (versus negative control [NC] siRNA) (**N**). (**O**) RT-qPCR analysis of *Acta2*, *Ccn2*, and *Serpine1* mRNA levels 6 hours after FLAG-tagged rCf48 stimulation in NRK49F cells pretreated with control or *Tfrc* siRNA. Data are expressed as mean ± SD. **P < 0.01; ****P < 0.0001 by 1-way ANOVA with Tukey’s multiple-comparisons test. NS, not significant.

While tubular cells are the main site of Cf48 production in the injured kidney, it is fibroblasts/myofibroblasts that are the main cell types responsible for pathogenic collagen deposition (26). Therefore, we hypothesized that Cf48 may enhance the fibrotic response in a paracrine fashion. We generated a recombinant form of the secreted Cf48 peptide (rCf48), which lacks the signal peptide (Figure 7A). The addition of rCf48 to NRK49F renal fibroblasts had no effect by itself, but enhanced the TGF-β1 profibrotic response (Figure 7, B and C). Similarly, addition of rCf48 to NRK52E tubular epithelial cells had no effect by itself, but enhanced TGF-β1–induced dedifferentiation, with increased expression of Acta2, collagen I, and fibronectin, and a reduction in cadherin-1 expression (Figure 7D).

Smad3 is activated by C-terminal phosphorylation and plays an essential role in the TGF-β1–induced fibrotic response (27). The addition of rCf48 to NRK49F renal fibroblasts did not induce Smad3 phosphorylation or enhance TGF-β1–induced Smad3 phosphorylation (Figure 7E). In addition, rCf48 failed to induce Smad3 transcriptional activity or modify TGF-β1–induced Smad3 transcriptional activity (Figure 7E). Pathways involved in noncanonical TGF-β1 signaling were investigated; however, rCf48 did not induce phosphorylation of p38 MAPK, ERK1/2, or JNK1/2 in renal fibroblasts, nor did Cf48 affect phosphorylation of these kinases in response to TGF-β1, TNF, or angiotensin II (Figure 7F and Supplemental Figure 15).

Next, we looked for evidence of rCf48-induced covalent modification of proteins in renal fibroblasts and proximal tubular epithelial cells 30 minutes after rCf48 addition. However, a Western blot–based screen using antibodies to detect phosphotyrosine, acetyllysine, propionyllysine, butyryllysine, succinyllysine, crotonyllysine, 2-hydroxyisobutyryllysine, malonyllysine, ubiquitin, sumo1/2/3, glutaryllysine, β-hydroxybutyryllysine, and lactyllysine modifications revealed no obvious changes (data not shown). As this appeared to rule out conventional receptor-based signaling pathways, we investigated whether the rCf48 peptide is endocytosed into cells in order to exert its effects without protein modification. To address this possibility, NRK49F cells were incubated with varying concentrations of FLAG-tagged rCf48 for different periods of time. Western blotting detected FLAG-rCf48 uptake in NRK49F cells after 30 minutes, which peaked at 2–4 hours, and uptake of FLAG-rCf48 increased as the amount of FLAG-rCf48 added to cells was increased (Figure 7G). Confocal imaging showed a perinuclear distribution of FLAG-rCf48 uptake in NRK49F cells at 2 hours (Figure 7H). To examine Cf48 uptake by cells in the fibrosing kidney, day 14 FAN mice were given FLAG-rCf48 by tail vein injection and then euthanized 5 minutes later. Confocal imaging revealed FLAG-rCf48 uptake by Acta2^+^ myofibroblasts in vivo (Figure 7I). These data suggest that Cf48 produced by tubular cells can act on fibroblasts in a paracrine fashion, although the mechanism of uptake into fibroblasts is unclear.

We investigated potential Cf48 binding proteins in 293T cells using immunoprecipitation coupled to MS (IP/MS), which identified TFRC (CD71) as a potential Cf48 receptor (Supplemental Table 6). TFRC is a cell surface receptor that takes up iron into the cell via receptor-mediated endocytosis and is a receptor for new-world arenaviruses (28, 29). Confocal microscopy demonstrated that TFRC is expressed in renal tubular epithelial cells and Acta2^+^ myofibroblasts (Figure 7J). IP/Western blotting and surface plasmon resonance studies confirmed the interaction between Cf48 and TFRC (Figure 7, K and L). Use of a neutralizing anti-TFRC antibody or siRNA-mediated *Tfrc* knockdown significantly reduced rCf48 uptake by NRK49F renal fibroblasts (Figure 7, M and N). Furthermore, *Tfrc* knockdown in NRK49F renal fibroblasts abrogated rCf48 enhancement of TGF-β1–induced *Acta2*, *Serpine1*, and *Ccn2* mRNA expression (Figure 7O). These data identify TFRC as the main receptor through which Cf48 enhances the TGF-β1–induced fibrotic response.

### Quantitative proteomic analysis identifies downregulation of extracellular matrix products in FAN in Cf48-deficient mice.

**Figure 8:**
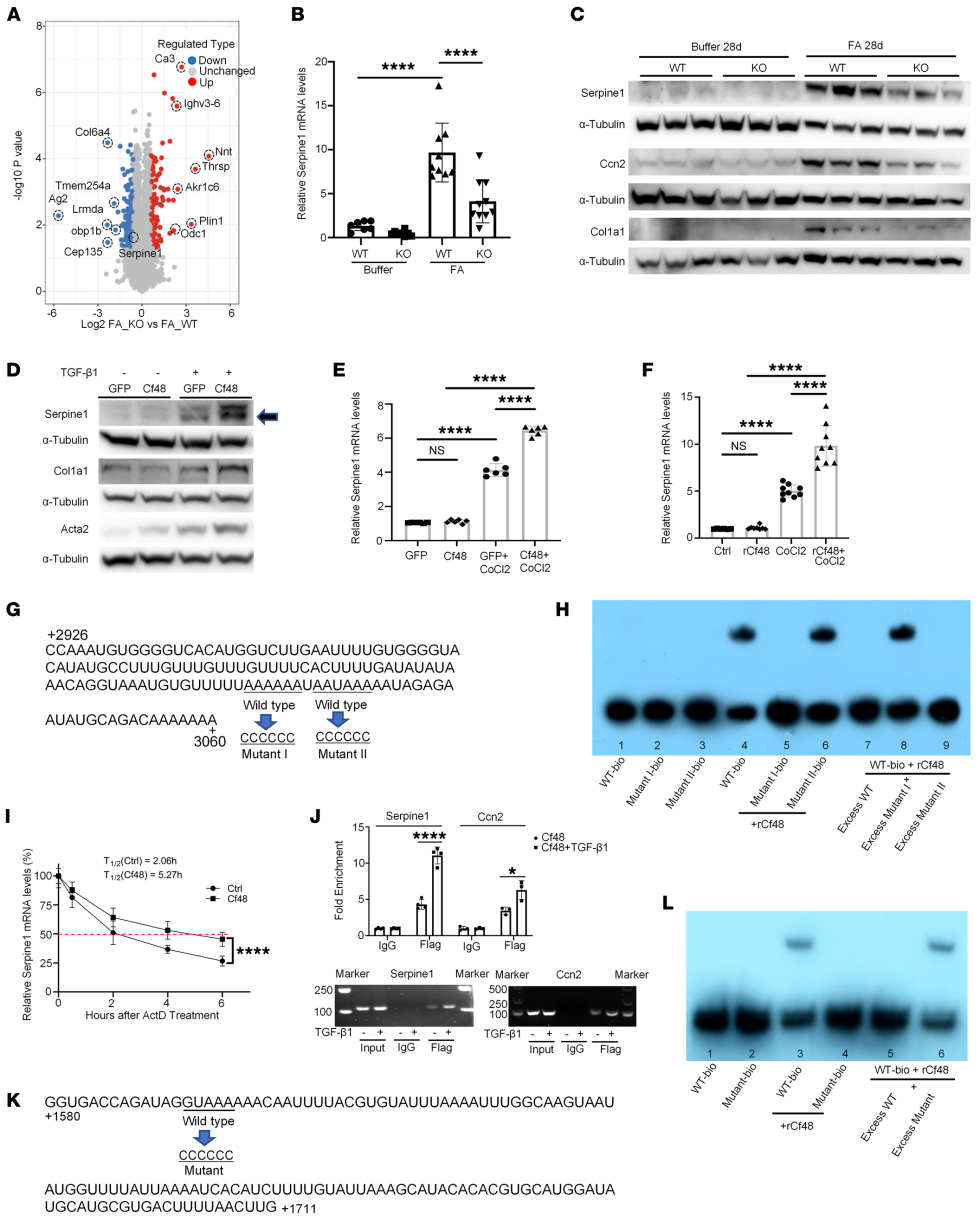
Cf48 is an mRNA-binding protein that regulates mRNA stability. (**A**) Volcano plot of changes in protein abundance on day 28 of folic acid-induced nephropathy (FAN) in WT or *Cf48*-KO kidneys. Average protein expression ratio of 3 replicates (log_2_ transformed) between FA KO and FA WT. Different treatment groups were plotted against *P* values obtained by 2-tailed Student’s *t* test (–log_10_ transformed). The cutoffs of *P* = 0.05 and 2-fold change are marked by blue and red dots, respectively. (**B**) RT-qPCR analysis of *Serpine1* mRNA levels in day 28 FAN or buffer-treated control WT or *Cf48*-KO kidneys. Data are expressed as mean ± SD. ****P < 0.0001 by 1-way ANOVA with Tukey’s multiple-comparison test. (**C**) Western blot (WB) analysis of Serpine1, Ccn2, and collagen I (Col1A1) protein levels in day 28 FAN and control WT and KO kidneys. (**D** and **E**) NRK49F cells were transduced with retroviral vector PMSCV-Cf48-IRES-GFP or PMSCV-IRES-GFP. GFP-positive cells were isolated by FACS and then cultured with or without TGF-β1 for 2 days for WB analysis (**D**), or cultured with and without CoCl_2_ for 6 hours and *Serpine1* mRNA levels were assessed by RT-qPCR (**E**). (**F**) NRK49F cells were cultured with or without rCf48 with or without CoCl_2_ for 6 hours and *Serpine1* mRNA levels were assessed by RT-qPCR. (**G**) Nucleotide sequence of *Serpine1* mRNA from position 2926–3060 with potential binding sites and their mutated versions shown. (**H**) RNA electrophoretic mobility shift assay (RNA-EMSA) analysis of binding interactions between biotin-labeled *Serpine1* probes and the rCf48 peptide. Lanes are the following: 1, bio-labeled WT (WT-bio) probe; 2, mutant I-bio; 3, mutant II-bio; 4, WT-bio + rCf48; 5, mutant I-bio + rCf48; 6, mutant II-bio + rCf48; 7, WT-bio + excess WT + rCf48; 8, WT-bio + excess mutant I + rCf48; and 9, WT-bio + excess mutant II + rCf48. (**I**) NRK49F cells were stimulated with TGF-β1 for 6 hours, and then actinomycin D was added and decay of *Serpine1* mRNA in the presence or absence of rCf48 was measured by RT-qPCR. RT-qPCR demonstrated levels of *Serpine1* mRNA after TGF-β1 with or without rCf48 treatment. ****P < 0.0001 by unpaired, 2-tailed Student’s *t* test. (**J**) Upper panel: RIP and RT-qPCR identified rCf48-binding mRNAs, *Serpine1* and *Ccn2*, while TGF-β1 increased the binding of Cf48 to *Serpine1* and *Ccn2* mRNAs in NRK49F cells. Data are expressed as mean ± SD. *P < 0.05; ****P < 0.0001 by 2-tailed Student’s *t* test. Lower panel: End products of RIP–RT-qPCR for *Serpine1* and *Ccn2* were visualized in agarose gels. (**K**) Nucleotide sequence of *Acta2* mRNA from position 1580–1711 with the potential Cf48 binding site, and its mutation. (**L**) RNA-EMSA for interactions between *Acta2* probes and the rCf48 peptide. Lanes are the following: 1, biotin-labeled WT (WT-bio) probe; 2, mutant-bio; 3, WT-bio + rCf48; 4, mutant-bio + rCf48; 5, WT-bio + excess WT + rCf48; and 6, WT-bio + excess mutant + rCf48.

To investigate the molecular mechanism(s) by which Cf48 promotes renal fibrosis, MS-based quantitative proteomic analysis of kidney samples from Cf48-deficient and WT mice on day 28 after FA or vehicle administration was performed and identified 8,286 quantifiable proteins in the 4 experimental groups with 3 biological replicates using 4D-DIA proteomic quantification. We defined significantly different (*P* < 0.05 by 2-tailed Student’s *t* test) proteins and used a criterion of 1.5-fold or greater change between 2 groups as differential protein candidates. Subsequently, the numbers of downregulated and upregulated proteins in the 4 groups were identified (Supplemental Table 7). Cluster of orthologous groups (COG) classification analysis revealed molecules in the category of translation and ribosomal structure and biogenesis were upregulated in the FAN KO group compared with the FAN WT group, while molecules classified in the categories of nucleotide transport and metabolism, RNA processing and modification, posttranslational modification, protein turnover, chaperones, and extracellular structures were downregulated (Supplemental Table 8). This indicated a selective effect of Cf48 deficiency on certain COGs. As one example, Serpine1 protein levels were reduced by more than 50% in the FAN KO group compared with the FAN WT group (Figure 8A). A separate analysis of kidney samples confirmed that Cf48 deficiency decreased Serpine1 production at the mRNA and protein levels in the FAN model (Figure 8, B and C). A retroviral vector was used to express green fluorescent protein (GFP), with or without full-length Cf48, in renal fibroblasts. Western blots of GFP-expressing cells showed that Cf48 overexpression enhanced TGF-β1–induced upregulation of Serpine1, Acta2, and collagen I proteins (Figure 8D). We investigated CoCl_2_-induced hypoxia-inducible factors as a second stimulus to induce Serpine1 expression. Use of virus-induced Cf48 overexpression, or the addition of rCf48 peptide, did not affect basal *Serpine1* mRNA levels in renal fibroblasts, but this did significantly increase CoCl_2_-induced upregulation of *Serpine1* mRNA (Figure 8, E and F). Given the importance of Serpine1 in renal fibrosis (30), we investigated how Cf48 regulates Serpine1 expression in renal fibroblasts.

### Cf48 is an mRNA-binding peptide that interacts with and stabilizes Serpine1 mRNA.

Cf48 increased Serpine1 production at mRNA and protein levels in renal fibroblasts. In addition, Cf48 interacts with SERBP1 (Supplemental Table 6), an mRNA-binding protein that binds to the 3′-most 134 nucleotides of *Serpine1* mRNA and acts to destabilize *Serpine1* mRNA (31). Using an RNA electrophoretic mobility shift assay (RNA-EMSA), we demonstrated that Cf48 can bind to 3′-most 134 nucleotides of *Serpine1* mRNA. Mutation at SERBP1 binding site 1, but not at binding site 2, abolished the interaction between rCf48 and *Serpine1* mRNA (Figure 8, G and H), indicating that Cf48 is an mRNA-binding peptide. Renal fibroblasts were stimulated with TGF-β1 for 6 hours to increase *Serpine1* mRNA levels, and then actinomycin D was added to stop transcription and enable mRNA decay assessment by RT-qPCR in the presence or absence of Cf48. rCf48 increased the half-life of *Serpine1* mRNA from an estimated 2.06 hours to 5.27 hours and the half-life of *Acta2* mRNA from 3.14 hours to 6.16 hours (Figure 8I and Supplemental Figure 16). Western blotting and an enzyme activity assay demonstrated that Cf48 enhanced TGF-β1–induced upregulation of Serpine1 protein level and activity after actinomycin D treatment in renal fibroblasts (Supplemental Figure 17). RNA immunoprecipitation-sequencing (RIP-seq) was performed for a broader view of Cf48 as an mRNA-binding protein. This found that Cf48 can bind to various RNAs (Supplemental Table 9), identifying 1,515 Cf48 target RNAs, including *Acta2*, *Serpine1, Ccn2*, and *Col4a1* (Supplemental Table 10 and Figure 8J). GO and KEGG analyses further revealed extracellular matrix structural constituent is one of the major Cf48-binding mRNAs (Supplemental Table 11). We also corroborated rCf48 binding to the 3′-most 132 nucleotides of the *Acta2* mRNA via RNA-EMSA (Figure 8, K and L). Vimentin (Vim) was not identified as a Cf48 target, and therefore served as a negative control in RNA-EMSA studies, with no interaction between rCf48 and the *Vim* mRNA probe seen (data not shown), even though it contains the proposed consensus rCf48 binding sequence, AAAAAA. These data suggest that other factors may be required for the interaction between the Cf48 peptide and the target RNA, which warrants further investigation.

To corroborate the proposed mechanism identified in animals, primary human renal fibroblasts were employed. The addition of Cf48 to human renal fibroblasts enhanced TGF-β1–induced production of Acta2, collagen I, and Serpine1 (Supplemental Figure 18A). Knockdown of TFRC by siRNA decreased uptake of FLAG-Cf48 and almost abrogated Cf48 enhancement of the TGF-β1–induced fibrotic response (Supplemental Figure 18, B–D). Furthermore, Cf48 increased the half-life of *Serpine1* mRNA (Supplemental Figure 18E). Taken together, our studies provided evidence that Cf48 promotes the TGF-β1–induced fibrotic response via the TFRC and an increase in the half-life of profibrotic gene mRNAs in both human and animal renal fibroblasts.

## Discussion

The present study identified Cf48 as a secreted micropeptide that is upregulated in both serum and kidneys across several types of human CKD (DN, LN, and IgAN), and upregulated in the kidney across 3 mouse CKD models. Serum Cf48 levels are strongly associated with loss of renal function in human DN. Overexpression, KO, or knockdown of Cf48 modulates renal fibrosis across 3 pathologically distinct mouse CKD models. Mechanistic studies demonstrate that TFRC mediates the uptake of Cf48 into renal fibroblasts. Cf48 interacts with mRNAs of profibrotic molecules, including *Acta2*, *Collagen IV*, *Ccn2*, and *Serpine1*, and stabilizes *Serpine1* mRNA. Our study provides robust evidence that Cf48 is a secreted peptide that enhances renal fibrosis and is a potential therapeutic target.

### Apoc1.

Ribosome profiling and bioinformatics analysis identified 4 secreted peptides as candidates to have a profibrotic function in mouse DN, including Cf48, Apoc1, Hilpda, and Tmsb4x. While Cf48 was selected for further investigation, 2 of the other peptides have been implicated in renal fibrosis. Apoc1 is an inhibitor of lipoprotein binding to the low-density lipoprotein (LDL) receptor, LDL receptor–related protein, and very-low-density lipoprotein receptor (32). Patients with DM have a higher Apoc1 plasma level, and meta-analysis demonstrates an association between a polymorphism in *Apoc1* and an increased risk of developing nephropathy (33). *Apoc1*-Tg mice exhibited albuminuria, renal dysfunction, glomerulosclerosis, inflammatory cell infiltration, and increased inflammatory cytokine and fibrotic growth factor expression (34), suggesting that Apoc1 may play a role in DN pathogenesis. Tmsb4x is a peptide that acts to suppress renal fibrosis (35), and loss of endogenous Tmsb4x accelerates kidney disease (36). The identification of these peptides supports the effectiveness of the screening strategy employed.

### Cf48 in the brain and the male reproductive organs.

Cf48 was first characterized by Endele et al. (37) as a putative evolutionarily conserved neuropeptide. It is 1 of the 3 genes within a DNA microdeletion found in a patient with a mild form of Wolf-Hirschhorn syndrome. Western blotting showed Cf48 protein expressed in brain tissue, but it was absent in other organs. In situ hybridization and Northern blotting demonstrated Cf48 expression in different zones during cortical and cerebellar development, and Cf48 expression in all cortical and subcortical regions of the adult mouse brain (37). The function of Cf48 was not studied, although Cf48 was proposed to be involved in intellectual and fine motor disabilities based on its pattern of expression. Very recently, Cf48 (NICOL1) was identified as a secreted protein expressed in mouse male reproductive organs. Cf48 plays an important role in lumicrine-mediated sperm maturation and male fertility through interaction with NELL2 (38). Male homozygous *Cf48*-KO mice are infertile (38). Our study confirmed that male homozygous *Cf48*-KO mice are infertile, and that Cf48 is expressed at very low levels in normal adult kidneys. However, Cf48 was upregulated in both human and mouse CKD. We also demonstrated that the secreted form of Cf48 enhances the TGF-β1–induced fibrotic response. In addition, our IP/MS study in 293T cells did not identify the interaction between Cf48 and NELL2 (Supplemental Table 6). Thus, it may be that Cf48 plays distinct roles in different organs.

*Do serum Cf48 levels predict progression of CKD?* Serum Cf48 peptide levels correlated with loss of kidney function, disease stage, and pathological grade of disease in human CKD. Indeed, renal interstitial fibrosis is a strong predictor of the progression of CKD to ESRD (4–6). However, renal fibrosis is a stochastic process, with biopsies showing varying degrees of active and inactive fibrosis, with active fibrotic lesions identified by the presence of Acta2^+^ myofibroblasts. The finding that serum Cf48 peptide levels correlated with the area of Acta2^+^ myofibroblast staining in renal biopsies, and that serum Cf48 levels were significantly increased while serum levels of creatinine reduced to a normal range in a mouse model of resolving AKI accompanying renal fibrosis, indicates that serum Cf48 could be a biomarker of active renal fibrosis, and potentially predictive of disease progression. However, our study could not rule out the possibility that the correlation between higher plasma Cf48 levels and reduced eGFR could be due to reduced glomerular filtration of the circulating Cf48 peptide.

Another limitation of our current study is that it uses a cross-sectional design at a single time point. To move these findings toward the clinic, our group is currently undertaking prospective studies in larger patient cohorts to define the range of serum Cf48 levels that are associated with active renal fibrosis, and to determine whether serum Cf48 levels can predict the loss of renal function over time.

### TFRC mediates uptake of Cf48 into renal tubular epithelial cells and fibroblasts.

TFRC can bind and internalize multiple ligands into the cell. While best known for the cellular uptake of iron through endocytosis (28), TFRC is a receptor for new-world hemorrhagic fever arenaviruses (29), and can endocytose IgA (39). The affinity (KD) between TFRC and Cf48 is 5.71 × 10^−7^ M, indicating that it is not a strong interaction and Cf48 may be easily released from TFRC after endocytosis. Antibody-based blockade or siRNA-mediated knockdown of TFRC was effective in preventing Cf48 uptake and function in renal fibroblasts. Supporting a role for TFRC in renal fibrosis are studies in which mice heterozygous for the *Tfrc* gene show reduced renal fibrosis in the UUO and DN models (40). However, how Cf48 interacts with TFRC remains to be investigated. Finally, it remains to be determined whether Cf48 can bind to other cell-surface receptors in kidneys.

### Cf48 is a potential antifibrotic target independent of TGF-β/Smad3 signaling.

TGF-β/Smad signaling plays a central role in fibrogenesis (41). However, TGF-β1 is a multifunctional protein involved in various processes, including cell differentiation, and is a negative regulator of the immune system. Indeed, complete TGF-β1 blockade causes uncontrolled immune-mediated organ destruction in neonatal mice (42). A key feature of the Cf48 peptide is that it enhances renal fibrosis without activating or altering the TGF-β/Smad signaling pathway. Thus, targeting endogenous Cf48 expression and/or function may circumvent the concerns associated with inhibiting the TGF-β/Smad signaling pathway itself. Supporting Cf48 as a potential therapeutic target, mice lacking *Cf48* are healthy and protected from renal fibrosis. Our study provides new therapeutic opportunities to target renal fibrosis. Cf48 expression may be inhibited via an siRNA or LNA-based oligonucleotide approach, while the function of the extracellular Cf48 peptide could be targeted by a neutralizing antibody. It may also be possible to design a TFRC antagonist that can prevent Cf48 binding, and thus Cf48 profibrotic action, without affecting transferrin uptake. Finally, further dissection of the mechanism of action of the Cf48 peptide may unveil a new paradigm in the regulation of the fibrotic response.

Our in vitro and in vivo studies provide evidence that Cf48 enhances renal fibrosis. However, renal fibrosis and inflammation are tightly linked in the pathogenesis of CKD, and effects of Cf48 on inflammation (cytokine expression and macrophage infiltration) were evident. Thus, a role for Cf48 in promoting inflammation may contribute to the overall profibrotic effect of Cf48 in the development and progression of CKD. Dissecting the contribution of Cf48 to inflammation per se warrants further investigation.

### Cf48 is an RNA-binding protein.

RNA-binding proteins (RBPs) interact with various RNAs to play fundamental roles in posttranscriptional and translational processes (43). Dysfunctional RBPs are associated with human diseases, such as genetic disorders (44) and cancer (45). Far upstream element–binding protein 1 (FUBP1) binds to the 3′UTR of polycystic kidney disease 2 (PKD2) mRNA to inhibit PKD2 translation (46). RIP-seq reveals that Cf48 interacts with extracellular matrix structural constituent mRNAs, including *Serpine1*, *Acta2*, *Ccn2*, and *Col4a1*. RNA-EMSA confirmed rCf48 binding to the *Serpine1* and *Acta2* mRNA consensus AAAAAA sequence, while an RNA decay study showed that Cf48 increased the half-life of *Serpine1* mRNA. The direct binding of SERBP1 to the 3′-most 134 nucleotides of the *Serpine1* mRNA increases its degradation (31). Cf48 may compete with SERBP1 to bind to *Serpine1* mRNA, thus avoiding mRNA degradation and enhancing mRNA accumulation and protein production, which promotes fibrosis. The proposed rCf48 binding mRNA consensus AAAAAA sequence is a relatively nonspecific binding site. We also identified potential Cf48 binding proteins in 293T cells using IP/MS (Supplemental Table 6). This could involve other protein(s) that are required to enable effective binding of the Cf48 peptide to the specific target mRNAs; however, investigation of such a possibility will be the subject of a new study.

### Tubulopathy versus glomerulopathy.

Renal tubular epithelial cells have a very high demand for energy to fulfill their extensive reabsorption and secretion functions, making these cells susceptible to diverse insults such as hypoxia, hypertension, hyperglycemia, proteinuria, toxins, and mechanical stress. Tubular epithelial cells may undergo dedifferentiation after injury, developing a profibrotic and proinflammatory phenotype that drives CKD progression (47). Our study revealed that Cf48 is predominantly expressed in renal tubular epithelial cells in CKD, and Cf48 promoted dedifferentiation in cultured proximal tubular epithelial cells. Cf48 overexpression or deletion/knockdown modulated renal interstitial fibrosis and CKD progression. Thus, while the Cf48 micropeptide clearly affects tubular epithelial cells and fibroblasts in the tubulointerstitial compartment of the kidney, the role of Cf48 in glomerular damage is much less clear. Of note, *Cf48*-Tg mice showed enhanced glomerular collagen deposition in the STZ-DN model, and Cf48 LNA treatment reduced glomerular collagen deposition in the *Nos3^–/–^* STZ-DN model. Thus, despite there being few Cf48-positive cells in the glomerular compartment, Cf48 still modified glomerulosclerosis. The impact of Cf48 on glomerular cells may be via direct interaction with mRNAs to promote fibrosis and/or inflammation, or via indirect actions through increased circulating levels of profibrotic and/or proinflammatory cues, such as Ccn2. Indeed, TRFC is upregulated in glomerular mesangial cells in patients with progressive IgAN and Henoch-Schonlein purpura (48, 49), although little information on podocyte TRFC expression is available. As the study used whole-body overexpression or KO of Cf48 in mice, the specific role of tubular cell production of Cf48 was not addressed, nor was the question of whether tubular cell–derived Cf48 acts in a paracrine fashion on renal interstitial fibroblasts and glomerular cells. The systemic effect could not be ruled out. Thus, an important area for future investigation is to determine how tubular cell–derived Cf48 acts to promote glomerulosclerosis and tubulointerstitial fibrosis in CKD.

In summary, our study identifies Cf48 as a micropeptide that enhances renal fibrosis independently of the TGF-β/Smad signaling pathway. A strong correlation was seen between serum Cf48 levels and loss of renal function. TFRC was identified as a Cf48 receptor, and Cf48 was shown to act as an RBP to regulate RNA metabolism and gene expression. These findings identify serum Cf48 levels as a potential biomarker of active renal fibrosis, while pharmacological approaches to inhibit Cf48 expression, neutralize the Cf48 peptide, or target the Cf48 peptide receptor, represent therapeutic opportunities for the treatment of CKD.

## Methods

### Sex as a biological variable.

Serum and biopsy samples were obtained from both males and females for analysis of Cf48 expression, with similar findings seen in both sexes. Female mice are resistant to STX-induced diabetes, and so this model was performed only in male mice. Both the UUO model (50) and the FA model (51) are induced equally in male and female mice; therefore, we performed studies in only male mice in order to minimize animal use (recognizing the goal of animal ethics for replacement, reduction and refinement), knowing that the results are highly likely to apply equally to both sexes.

### Study design.

The objective of the study was to investigate the role of the Cf48 micropeptide in CKD. Serum levels of the Cf48 peptide were measured by ELISA (CSB-EL003989HU, CUSABIO) in a cohort of patients with CKD (DN, IgAN, and LN) undergoing renal biopsy in the Department of Nephrology, the First Affiliated Hospital of Sun Yat-Sen University from June 2020 to December 2021 (see Supplemental Table 4). Controls included diabetic individuals without kidney disease (DM)and healthy controls (serum only). Serum Cf48 levels were compared between groups and correlated with kidney function and features of kidney biopsies. Indirect immunofluorescent staining for Cf48 was performed in formalin-fixed, paraffin-embedded sections of renal biopsies using rabbit monoclonal anti-Cf48 antibody (ab185315, Abcam).

Tg mice with doxycycline-inducible ubiquitous expression of *C4orf48* (gm1673) under the CAG promoter, and *Cf48*-KO mice, were created by Cyagen Biosciences (Supplemental Figures 19 and 20). Homozygous *Cf48*-KO and WT mice were produced by crossbreeding of heterozygous mice. Male homozygous *Cf48*-KO mice were infertile, which is consistent with the report by Kiyozumi et al. (38). *Nos3^–/–^* mice were obtained from The Jackson Laboratory (strain 002684). All mouse lines were on the C57BL/6J background and bred at Sun Yat-Sen University Animal Services and Guangdong Medical University Animal Services. Age- and sex-matched littermates were used as WT controls.

Mice were used in 3 contrasting models of CKD. DN was induced in 8-week-old mice by low-dose STZ (Sigma-Aldrich; 55 mg/kg) in 0.1 mmol/L sodium citrate buffer (pH 4.5) given intraperitoneally once daily for 5 days (16). FAN was induced in 8-week-old mice by intraperitoneal injection of 250 mg/kg FA (Sigma-Aldrich) in 0.3 mol/L sodium bicarbonate, with AKI assessed by renal function on day 2 and CKD assessed on day 28 (22). Renal interstitial fibrosis was induced by UUO surgery, as previously described (23). Measurement of kidney function, blood glucose levels, kidney histology, kidney immunostaining, RNA analysis, and Western blotting are described in the Supplemental Methods.

Mouse Cf48 LNA and control LNA were designed by and purchased from QIAGEN. LNA sequences are shown in Supplemental Methods. In the UUO model, LNAs were administered by intraperitoneal injection on days 1 and 4 after surgery and then animals were euthanized on day 7. In the STZ-DN model induced in *Nos3^–/–^* mice, LNAs were administered by weekly injection on weeks 3 to 8 after STZ administration and then animals were euthanized.

The ability of recombinant Cf48 peptide to enhance the fibrotic response of normal rat kidney fibroblasts (NRK49F cells, CRL-1570, ATCC) or normal rat kidney epithelial cells (NKR52E cells, CRL-1571, ATCC) was tested in the presence and absence of TGF-β1. Recombinant FLAG-tagged mouse Cf48 peptide (see Supplemental Table 12) was purified from CHO cells and used in studies of peptide uptake by renal fibroblasts and tubular cells in vitro and in vivo. IP/MS identified the TFRC (CD71) as a potential Cf48 receptor. Use of a neutralizing anti-TFRC antibody or *Tfrc* siRNA blocked Cf48 peptide uptake and biological response in NRK49F cells.

Kidney tissues from *Cf48*-KO and WT mice on day 28 of FAN were analyzed by MS-based 4D-DIA proteomic quantification and COG classification, which showed downregulation of extracellular matrix products in *Cf48*-KO mice. The role of Cf48 as an RBP for mRNA species involved in the fibrotic response was investigated by RIP-seq, RNA-EMSA, and RNA decay studies.

### Statistics.

Data are presented as mean ± SD. Statistical comparisons between 2 groups were conducted using the 2-tailed Student’s *t* test, or by 1-way analysis of variance (ANOVA) followed by Tukey’s multiple-comparison test for 3 or more groups. Correlation analysis of parametric data without normal distribution used Spearman’s coefficient. A *P* value of less than 0.05 was considered significant. Analyses were performed using Prism version 8.0 (GraphPad Software) and SPSS 24.0 software (IBM).

### Study approval.

The human study was reviewed and approved by the First Affiliated Hospital of Sun Yat-Sen University Institutional Review Board (IRB approved number [2016] 215, Guangzhou, China). All patients gave their written informed consent. All animal studies were reviewed and approved by the Sun Yat-Sen University Institutional Animal Care and Use Committee (nos. SYSU-IACUC-2022-000134, -000361, and -000943) and the Guangdong Medical University Institutional Animal Care and Use Committee (no. GDY2204005).

### Data and materials availability.

All data are available in the main text or the Supplemental Material, including the Supporting Data Values file.

## Author contributions

JL and DJNP conceptualized the study. JL, DJNP, JY, HZ, ABNN, NL, HC, XQ, JF, XZ, and DW developed methodology. JL, JY, HZ, ABNN, NL, HC, AL, XQ, QW, JF, XB, ZY, BG, YM, XZ, DW, YS, XJ, WC, ANC, DJNP, and XY conducted experiments. JL, JY, HZ, NL, HC, AL, XQ, QW, JF, XB, ZY, YS, XJ, WC, DJNP, and XY generated figures. JL, DNP, JY, JF, XB, XJ, WC, and XY acquired funding. JL and JF provided project administration. JL, DJNP, XJ, WC, and XY supervised the study. JL, JY, and HZ wrote the original draft of the manuscript, which was reviewed and edited by JL, DJNP, AL, JY, HZ, XJ, WC, and XY.

## Supplementary Material

Supplemental data

Unedited blot and gel images

Supplemental table 1

Supplemental table 10

Supplemental table 11

Supplemental table 12

Supplemental table 13

Supplemental table 14

Supplemental table 2

Supplemental table 3

Supplemental table 4

Supplemental table 5

Supplemental table 6

Supplemental table 7

Supplemental table 8

Supplemental table 9

Supporting data values

## Version 1 04/16/2024

In-Press Preview

## Version 2 05/15/2024

Electronic publication
