# Supplementary material for: The secreted micropeptide C4orf48 enhances renal fibrosis via an RNA-binding mechanism
Source: J Clin Invest. 2024 Apr 16;134(10):e178392. doi: 10.1172/JCI178392 (PMC11093611; doi:10.1172/JCI178392)
Supplement: Supplemental data [file jci-134-178392-s175.pdf]

Supplementary Figure S1

(A)

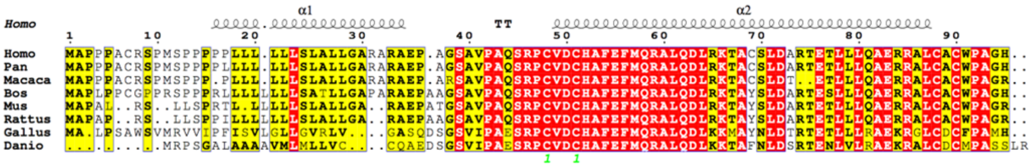

(B) Mouse Cf48

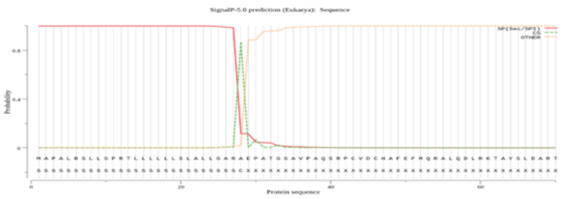

Human Cf48

(C)

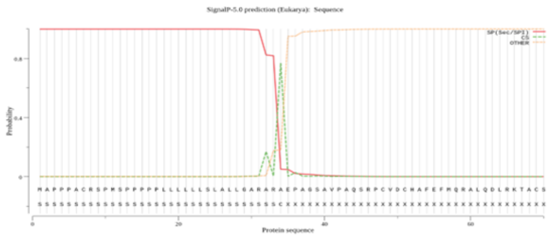

(D)

ID	Accession	Gene Name	FC (Cf48_vs_Ctrl)	pValue (Cf48_vs_Ctrl)	FDR (Cf48_vs_Ctrl)
CD048_HUMAN	Q5BLP8	C4orf48	34.90332	0.000171	0.031563
PRPC_HUMAN	P02810	PRH2	3.274368	0.00077	0.047065
EVPL_HUMAN	Q92817	EVPL	2.992943	0.000792	0.047065
REEP5_HUMAN	Q00765	REEP5	2.865452	0.000424	0.034383
DECR_HUMAN	Q16698	DECR1	2.687202	0.000422	0.034383

Supplementary Figure S1*. *Cf48 is a highly conserved and secreted micropeptide.

(A) A comparison of the micropeptide sequence of Cf48 between human and several different species using multiple sequence alignment is shown. In the alignment, some positions are denoted in red or yellow, which indicate absolute conservation or a high degree of conservation across species, respectively. SignalP-5.0 predicted the signal peptide of mouse (B) and human (C) Cf48. Cf48 expression in Cf48 transgene 293T cell culture medium. (D) Components in conditioned medium 24hrs after Cf48 transgene in 293T cells detected by Mass spectrometry. N=5/group. Unpaired t test.

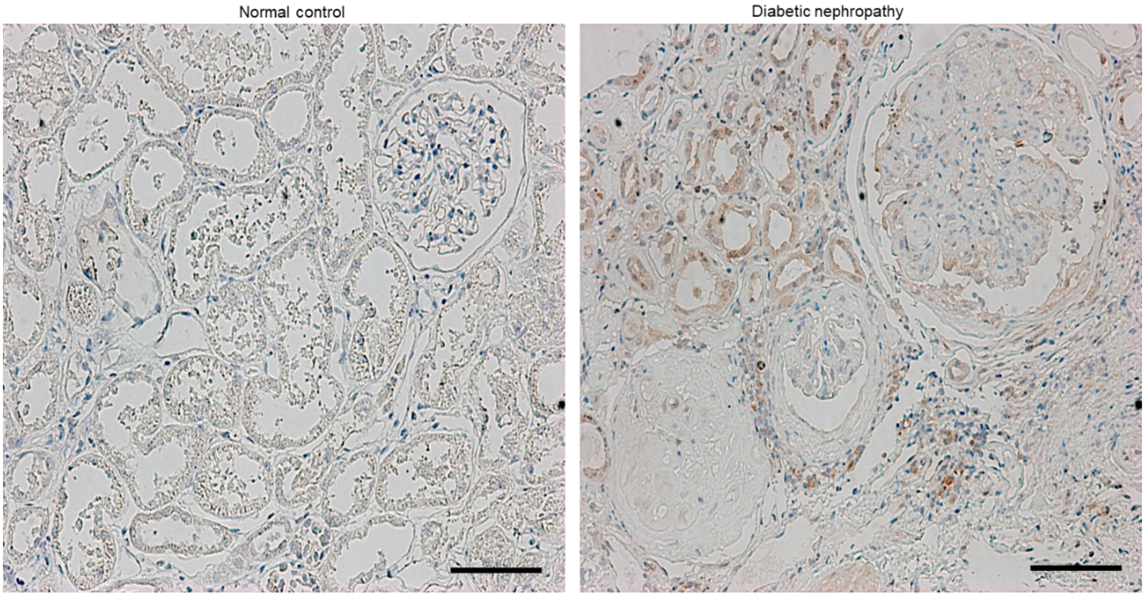

Supplementary Figure S2. Cf48 expression in human kidneys. *Immunostaining demonstrating Cf48 expression (brown) in human normal control and diabetic nephropathy. Scale bar, 100μm.*

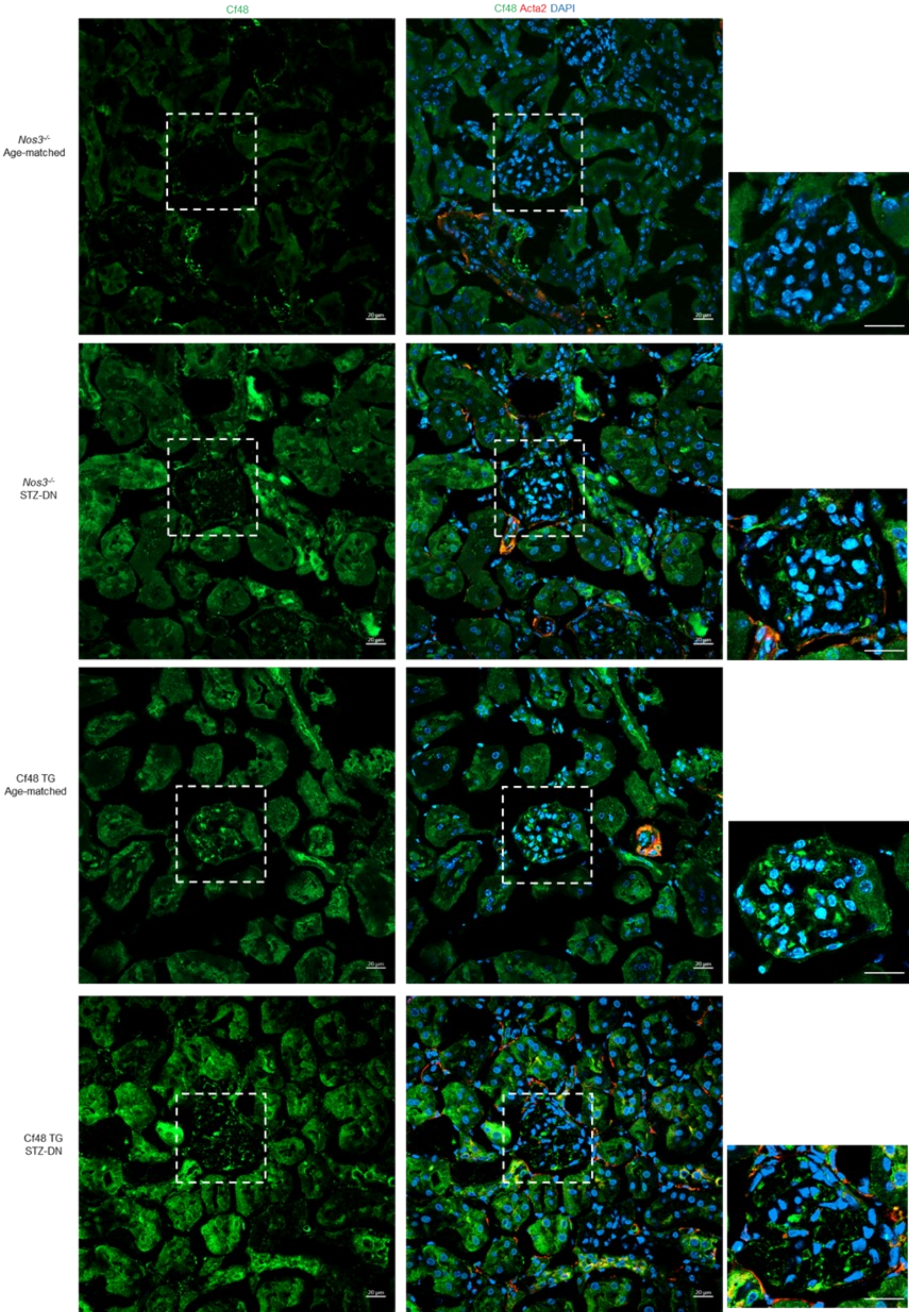

Supplementary Figure S3. Cf48 expression in mouse kidneys. *Confocal microscopy demonstrating Cf48 staining (green), DAPI (cyan), Acta2 (red) and merged images in 6-week streptozotocin (STZ)-induced diabetic nephropathy (DN) in Nos3 knockout*

(Nos3$^{-/-}$) and Cf48 transgenic (TG) mice. Age-matched mice as controls. Scale bar, 20μm. Square frames: location of the glomeruli; insets: digital enlargement of the glomeruli.

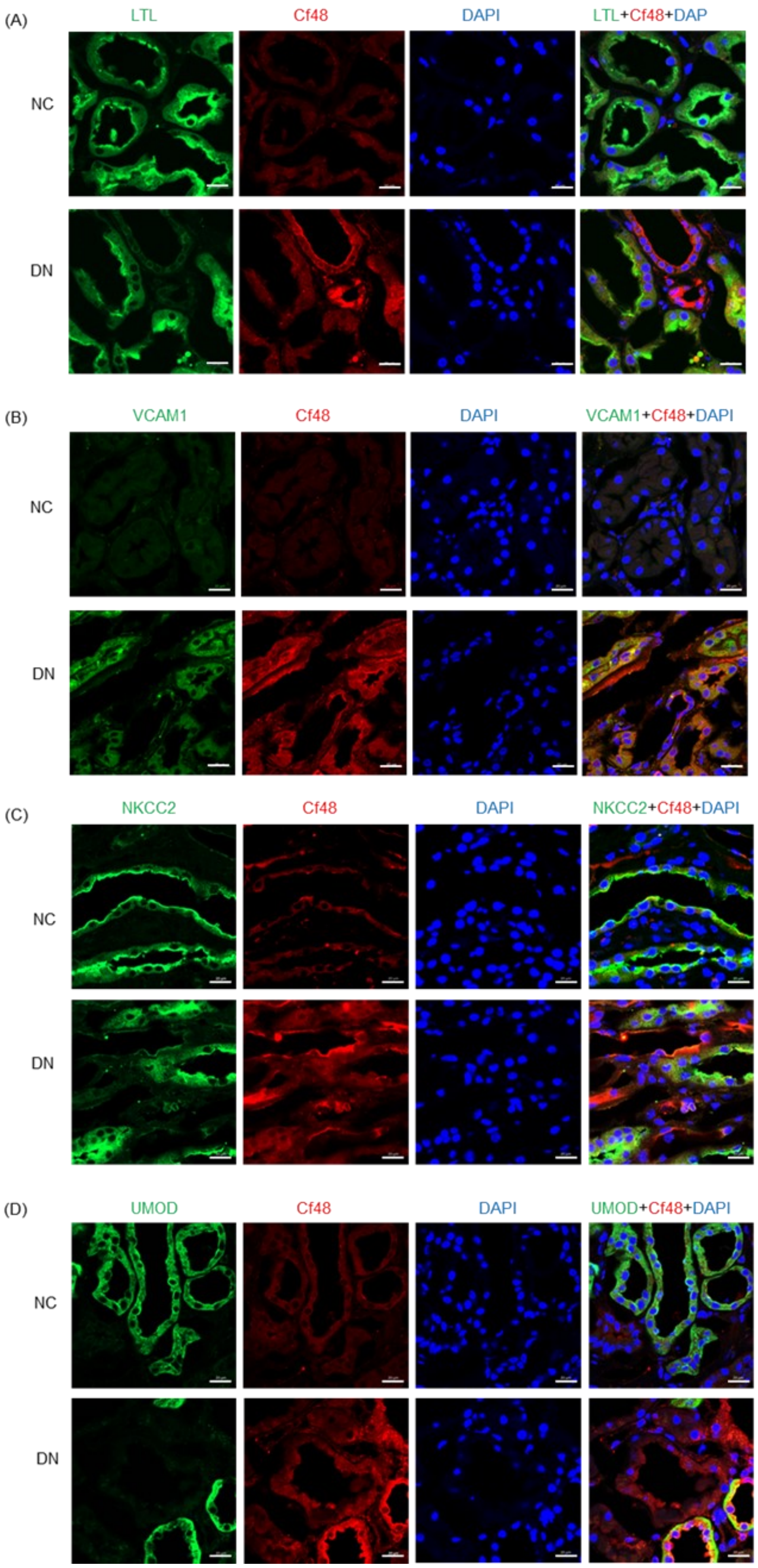

Supplementary Figure S4. Expression of Cf48 in normal control and human diabetic nephropathy. *Confocal microscopy shows staining of LTL (green, A),*

VCAM1 (green, B), NKCC2 (green, C), UMOD (green, D), Cf48 (red, A-D), DAPI (blue, A-D) in normal control (NC) and human diabetic nephropathy (DN). The merged images show: (A) LTL+Cf48+DAPI; (B) VCAM1+Cf48+DAPI; (C) NKCC2+Cf48+DAPI, and; (D) UMOD+Cf48+DAPI. Scale bar, 20 μm.

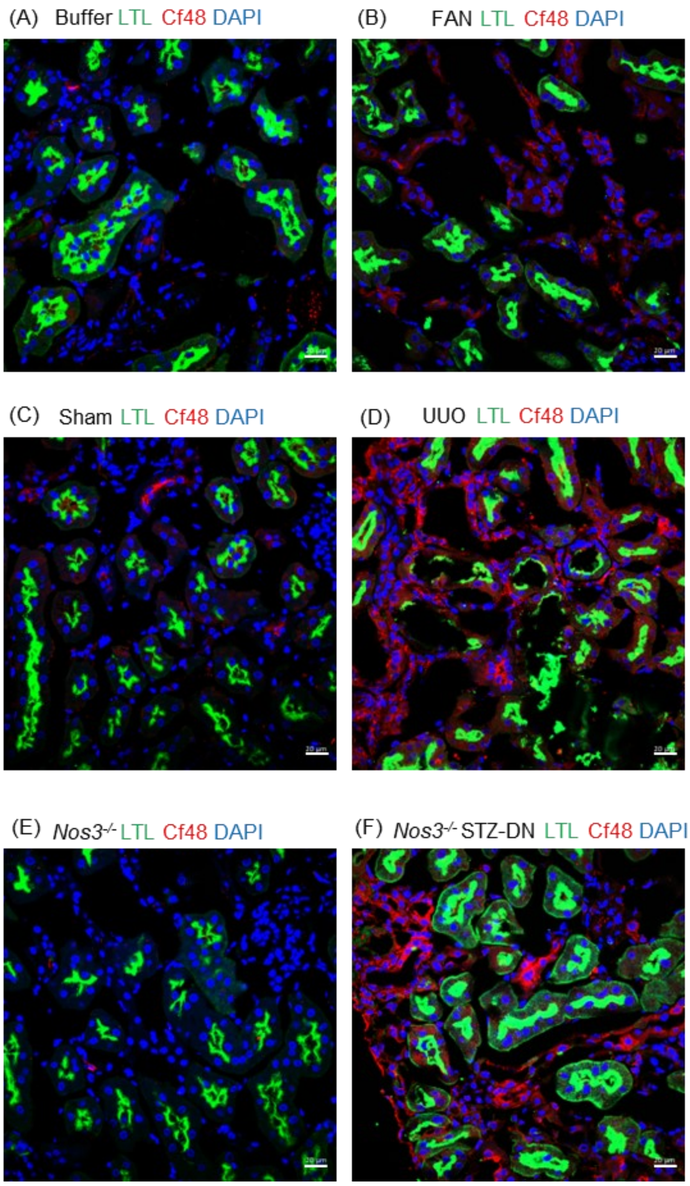

Supplementary Figure S5. Expression of Cf48 in normal control and 3 mouse models of renal fibrosis. *Confocal microscopy shows staining of LTL (green, A-F), Cf48 (red, A-F) and DAPI (blue, A-F). (A) buffer injected control mouse. (B) Day 14 of folic acid-induced nephropathy (FAN). (C) Sham operated control mouse. (D) Day 7 unilateral ureteral obstruction (UUO). (E) Control Nos3*$^{-/-}$ *mouse. (F) Streptozotocin (STZ)-induced diabetic nephropathy in Nos3*$^{-/-}$ *mice. Scale bar, 20 µm.*

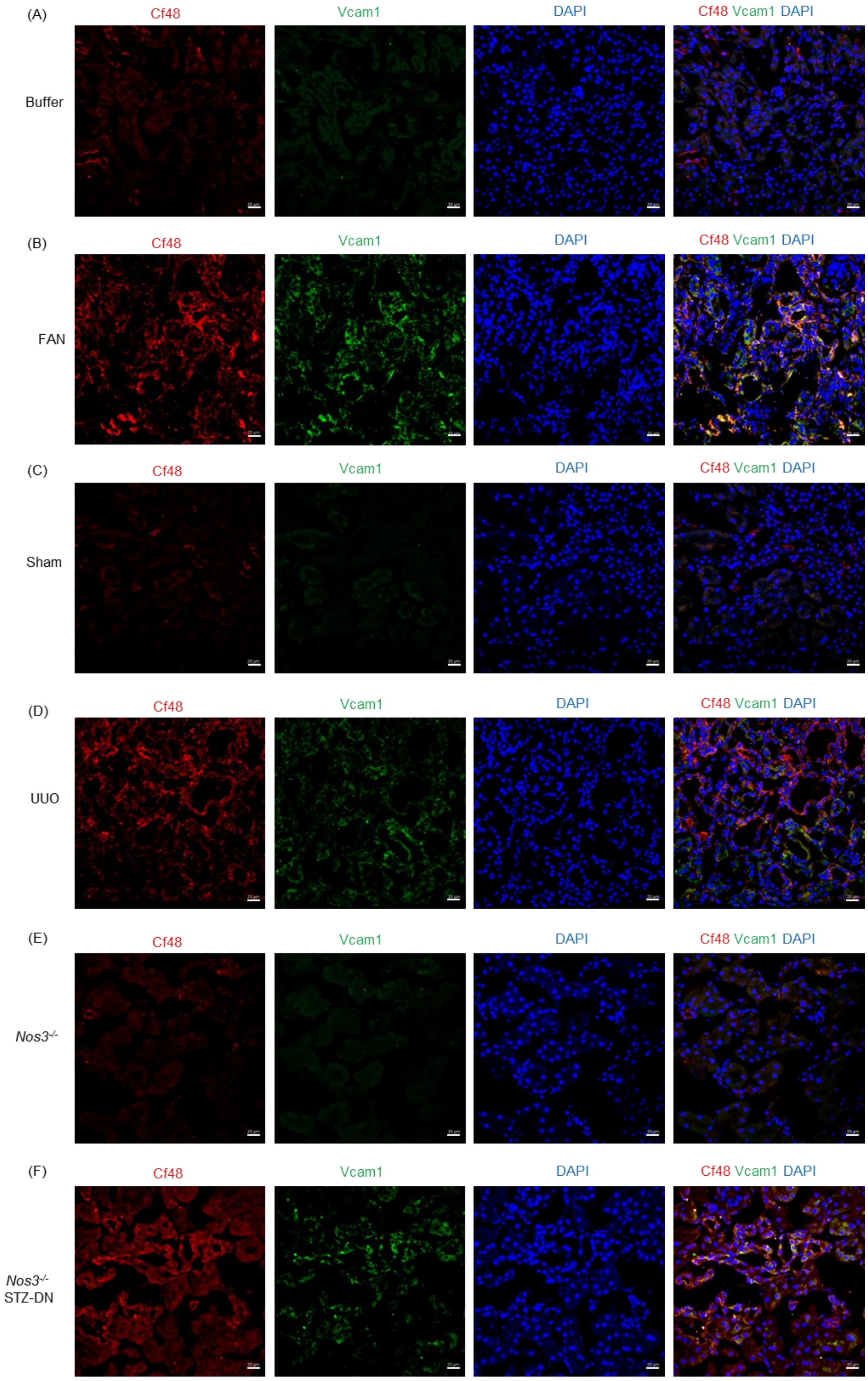
(A)
Cf48
Vcam1
DAPI
Cf48 Vcam1 DAPI
Buffer
(B)
FAN
(C)
Sham
(D)
UUO
(E)
Nos3-/-
(F)
Nos3-/- STZ-DN

Supplementary Figure S6. Expression of Cf48 and Vcam1 in normal control and 3 mouse models of renal fibrosis. *Confocal microscopy shows staining of Cf48 (red, A-F), Vcam1 (green, A-F) and DAPI (blue, A-F). (A) buffer injected control mouse. (B) Day 14 of folic acid-induced nephropathy (FAN). (C) Sham operated control mouse. (D) Day 7 unilateral ureteral obstruction (UUO). (E) Control Nos3*$^{-/-}$ *mouse. (F) Streptozotocin (STZ)-induced diabetic nephropathy (STZ-DN) in Nos3*$^{-/-}$ *mice. Scale bar, 20 µm.*

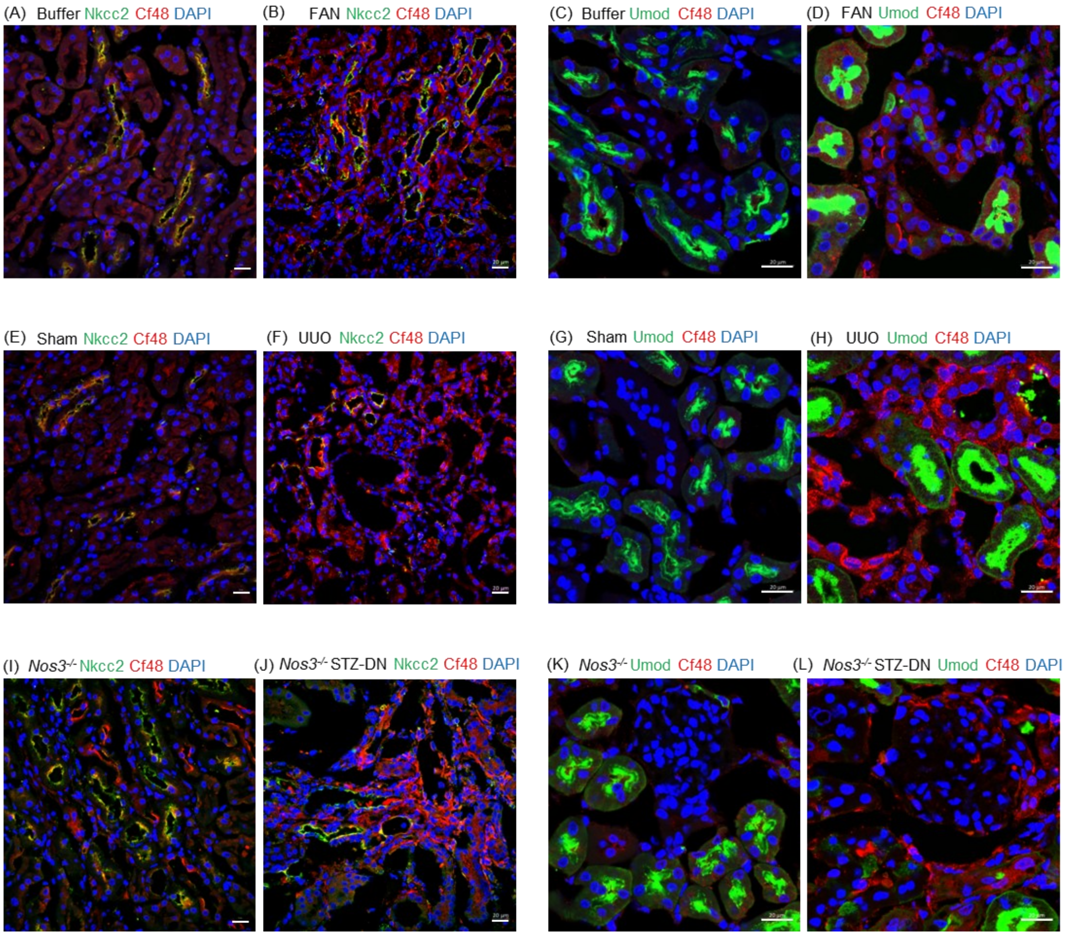

***Supplementary Figure S7. Expression of Cf48, Nkcc2 and Umod in normal control and 3 mouse models of renal fibrosis**. Confocal microscopy shows staining of Nkcc2 (green, A, B, E, F, I and J), Cf48 (red, A-L), Umod (green, C, D, G, H, K, L) and DAPI (blue, A-L). (A and C) buffer injected control mouse. (B and D) Day 14 of folic acid-induced nephropathy (FAN). (E and G) Sham operated control mouse. (F and H) Day 7 unilateral ureteral obstruction (UUO). (I and K) Control Nos3$^{-/-}$ mouse. (J and L) Streptozotocin (STZ)-induced diabetic nephropathy in Nos3$^{-/-}$ mice. Scale bar, 20 µm.*

(A)

Human IgAN		
Clinical index	Spearman	*P Value*
CKD stage	0.320	0.169
Pathological grade	0.564	0.023
BUN	0.211	0.371
Creatinine	0.280	0.232
Serum albumin	0.090	0.705
Fasting glucose	0.078	0.743
HbAC1	0.543	0.266
Uric acid	0.143	0.583
24h urinary protein	0.074	0.758
eGFR	-0.355	0.125

n=20; *P<0.05

(B)

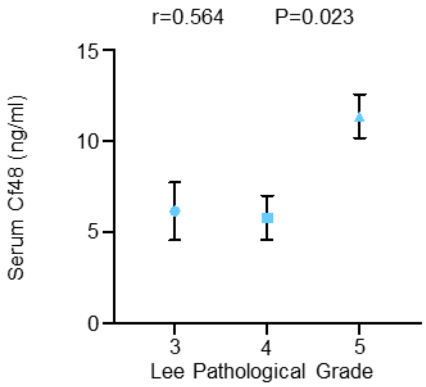

Supplementary Figure S8. Correlations between clinical indices and serum Cf48 levels in human IgAN*. (A) Table of correlations between clinical indices and serum Cf48 levels in IgA nephropathy (IgAN). (B) Correlation between serum Cf48 levels and the Lee pathological grade in IgAN.*

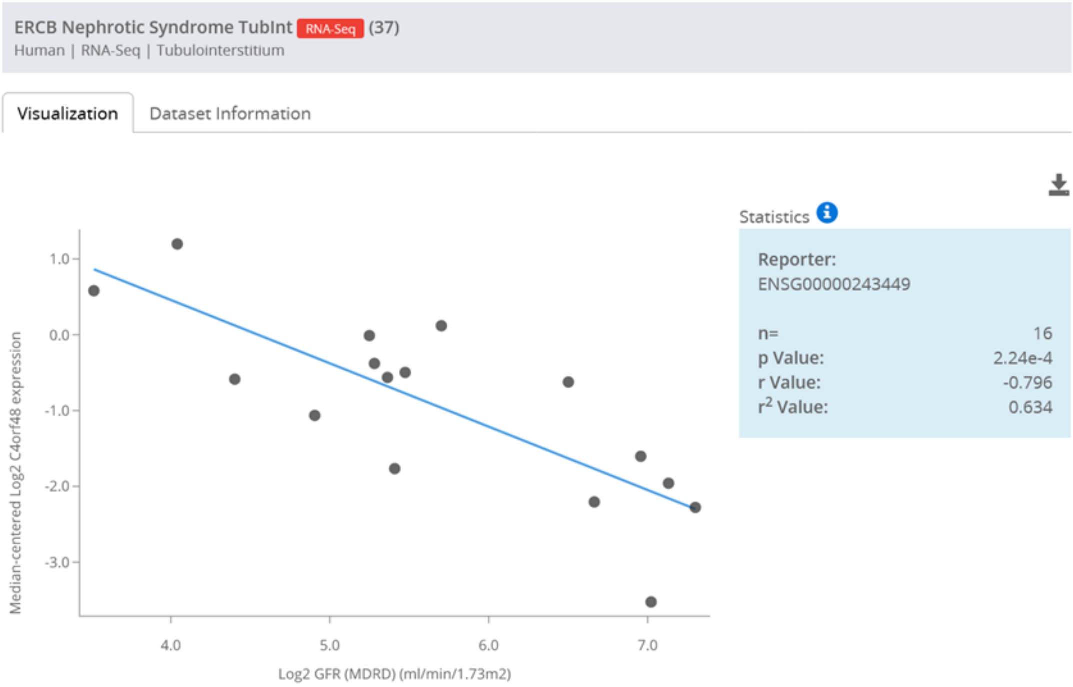

Supplementary Figure S9. Cf48 mRNA levels in the kidney tubulointerstitium correlate with renal function in chronic kidney disease. *Data extracted from the NephroSeq database (http://v5.nephroseq.org) in which glomerular filtration rate (GFR) is compared to Cf48 mRNA levels in a study of 28 patients with chronic kidney disease and 9 controls from the European Renal cDNA Bank-Kroener-Fresenius biopsy bank. RNA sequencing was performed on microdissected tubulointerstitial tissue from human kidney biopsies as described in Martini S, Nair V, Keller BJ, Eichinger F, Hawkins JJ, Randolph A, Böger CA, Gadegbeku CA, Fox CS, Cohen CD, Kretzler M. European Renal cDNA Bank; C-PROBE Cohort; CKDGen Consortium. Integrative biology identifies shared transcriptional networks in CKD. J Am Soc Nephrol. 2014 25:2559-72.*

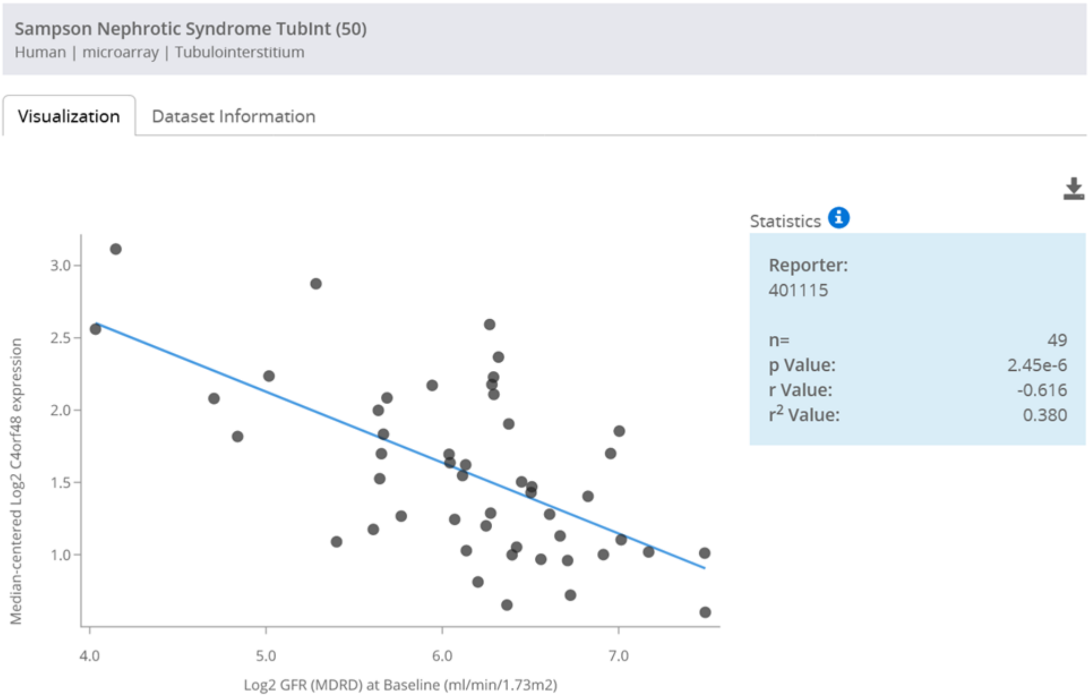

Supplementary Figure S10. Kidney Cf48 mRNA levels correlate with renal function in chronic kidney disease. *Data extracted from the NephroSeq database (http://v5.nephroseq.org) in which glomerular filtration rate (GFR) is compared to kidney Cf48 mRNA levels in a study of 50 patients in the Nephrotic Syndrome Study Network (NEPTUNE). Patients included those with focal segmental glomerulosclerosis, minimal change disease, membranous nephropathy, or other glomerulopathies. Gene expression profiling was performed on either micro-dissected glomerular samples (n=38) or tubulointerstitial samples (n=50) using the Affymetrix Human Gene 2.1 ST Array as described in Sampson MG, Robertson CC, Martini S, Mariani LH, Lemley KV, Gillies CE, Otto EA, Kopp JB, Randolph A, Vega-Warner V, Eichinger F, Nair V, Gipson DS, Cattran DC, Johnstone DB, O'Toole JF, Bagnasco SM, Song PX, Barisoni L, Troost JP, Kretzler M, Sedor JR. Nephrotic Syndrome Study Network. J Am Soc Nephrol. 2016 27:814-23.*

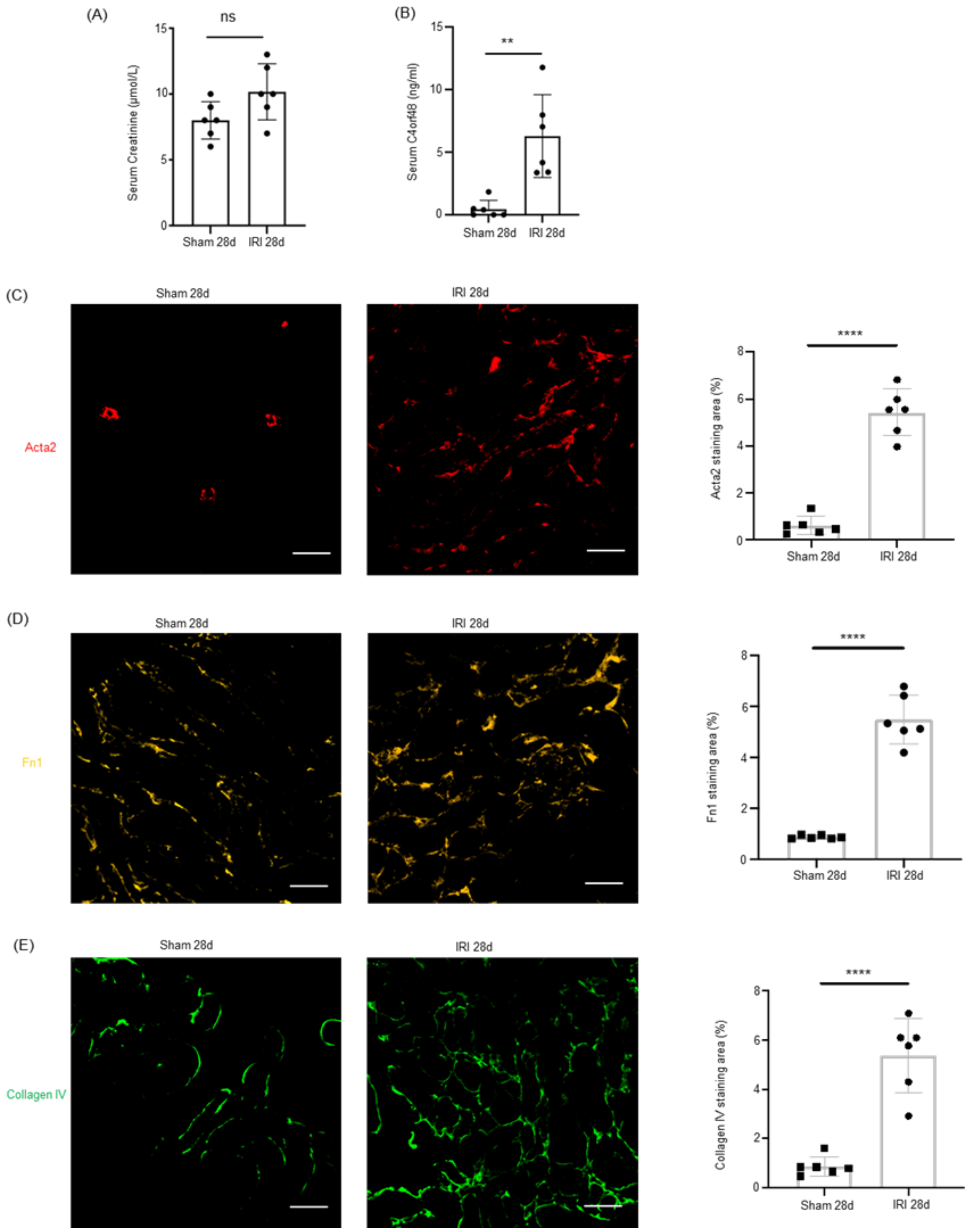

Supplementary Figure S11. Serum Cf48 levels are increased in mice with developing renal fibrosis following ischemia reperfusion injury (IRI). *Sham or renal bilateral 15-min ischemia operation was performed in C57BL/6J mice. Mice were killed 28 days after sham or IRI operation. Serum levels of Creatinine (A) and C4orf48 (B) 28 days after sham or IRI operation. Immunostaining of Acta2, Fn1 and*

*collagen IV and quantitation of staining area (C-E). Unpaired t test. ns, no significance; **, $p<0.01$; ****, $p<0.0001$. Scale bar, 50μm.*

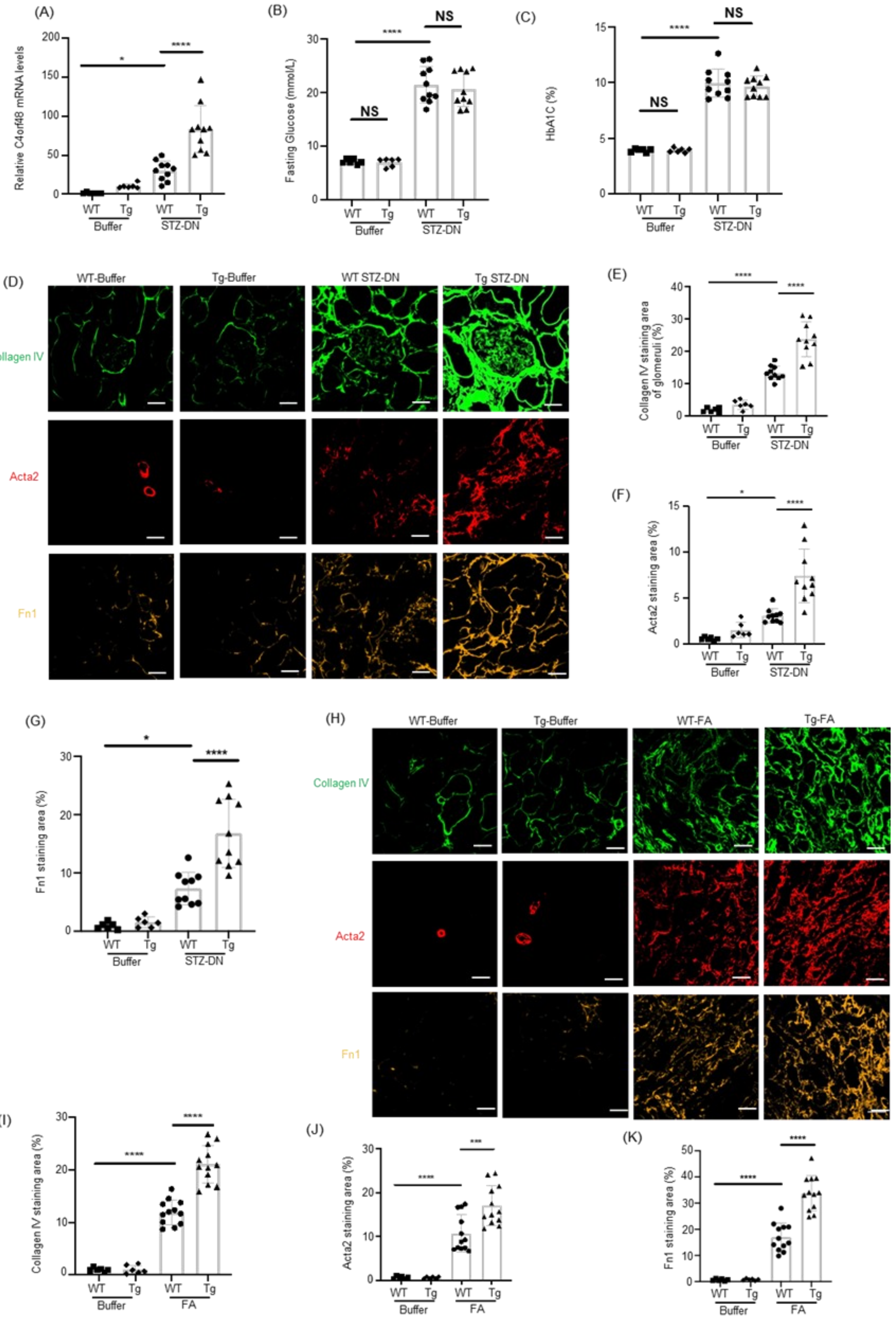

Supplementary Figure S12. Overexpression of Cf48 promotes renal fibrosis in mouse models of CKD. *(A) C4orf48 mRNA levels, (B) fasting blood glucose levels,*

*(C) plasma HbA1c levels, in 12-week buffer-treated or streptozotocin (STZ)-induced diabetic nephropathy (DN) in wild type (WT) and C4orf48 transgenic (Tg) mice. Confocal microscopy shows staining of collagen IV (green), Acta2 (red), Fn1 (yellow) and DAPI (blue) in 12-week buffer-treated or streptozotocin (STZ)-induced diabetic nephropathy (D). (E-G) Quantification of staining area of collagen IV, Acta2 and Fn1. Confocal microscopy shows staining of collagen IV (green), Acta2 (red), Fn1 (yellow) and DAPI (blue) in 4-week buffer-treated or folic-acid (FA)-induced nephropathy in WT or C4orf48 Tg mice (H). (I-K) Quantification of staining area of collagen IV, Acta2 and Fn1. Data were expressed as mean ± SD. One-way ANOVA with Tukey's multiple comparisons test. NS, not significant; *P<0.05; ***P<0.001; ****P<0.0001. Scale bar, 50 µm.*

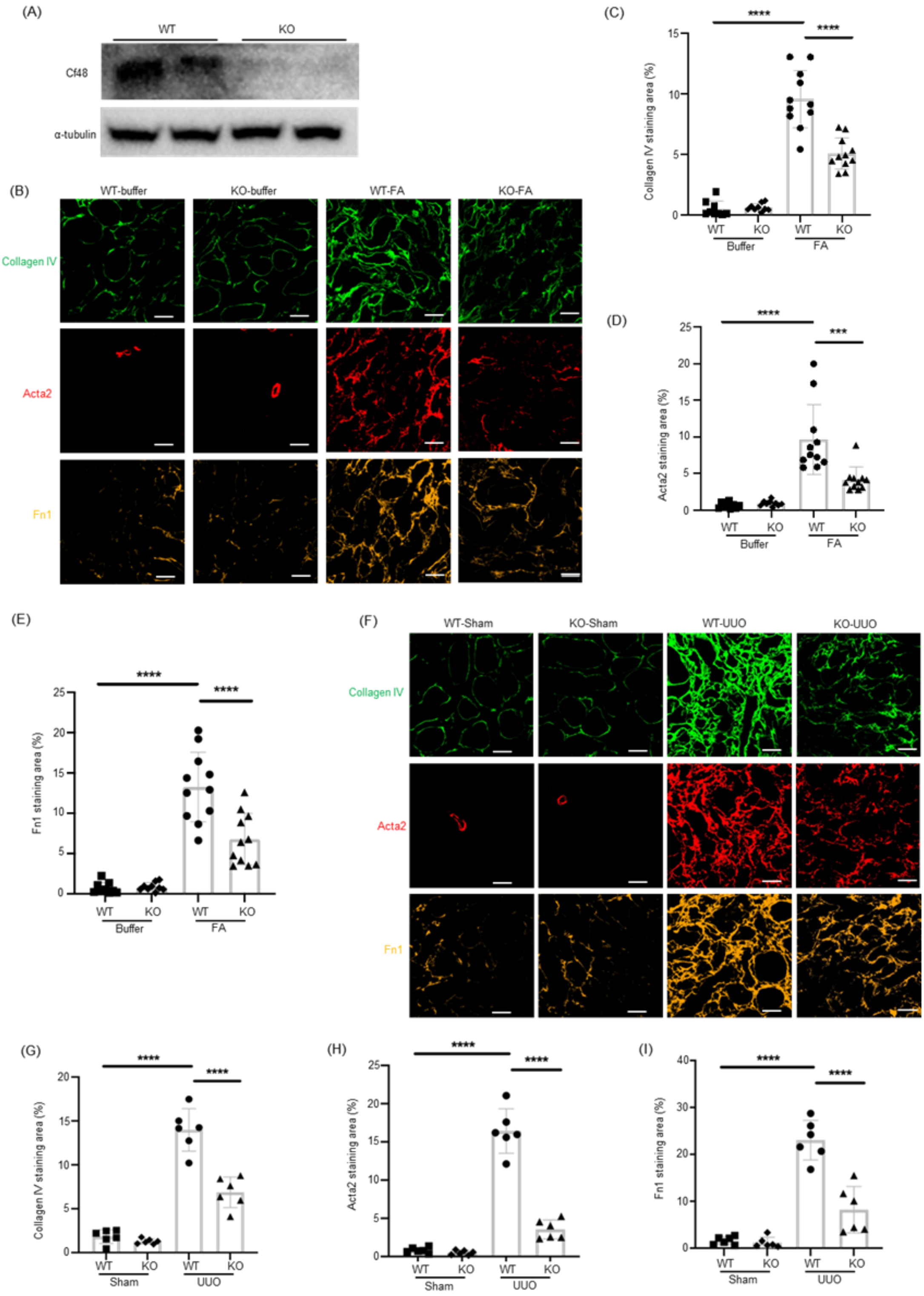

Supplementary Figure S13. Cf48 deficiency suppressed renal fibrosis in mouse models of CKD. *(A) Western blotting showing Cf48 expression in 28-day folic acid-induced nephropathy in wild type (WT) and Cf48 knockout (KO) mice. Confocal microscopy shows staining of collagen IV (green), Acta2 (red), and Fn1 (yellow) in 4-*

*week buffer-treated kidneys or folic-acid (FA)-induced nephropathy (B). (C-E) Quantification of collagen IV, Acta2, and Fn1 staining area. (F) Confocal microscopy shows staining of collagen IV (green), Acta2 (red), and Fn1 (yellow) in 7-day sham or UUO surgery kidneys in wild type (WT) or Cf48 deficient (KO) mice. (G-I) Quantification of collagen IV, Acta2, and Fn1 staining area. Data are expressed as mean ± SD. One-way ANOVA with Tukey's multiple comparisons test. ***P<0.001; ****P<0.0001. Scale bar, 50 μm.*

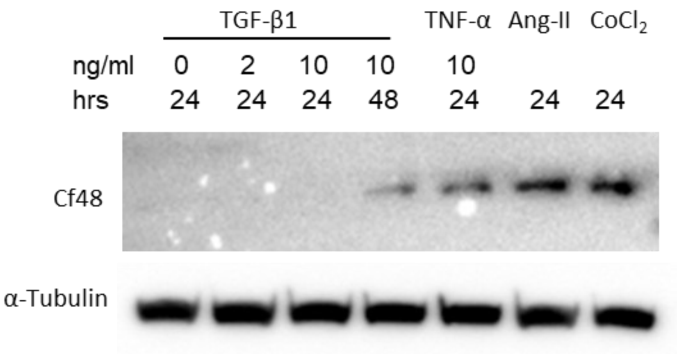

Supplementary Figure S14. Induction of Cf48 expression under various conditions in HK2 cells. *Western blotting shows Cf48 expression in HK2 cells following stimulation with TGF-β1, TNF-α, Angiotensin II (10^{-6}M) or CoCl2 (10^{-4}M) for various times.*

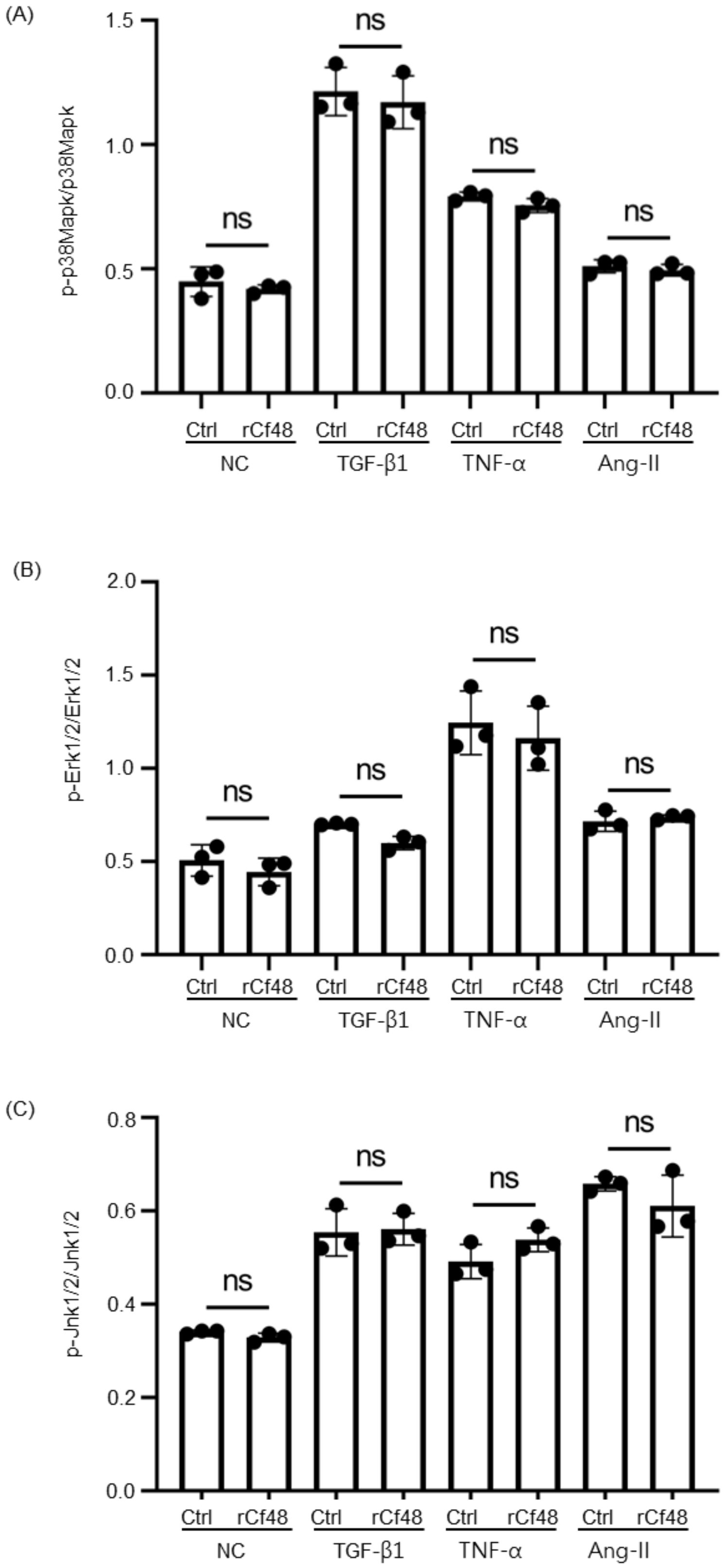

Supplementary Figure S15. Effect of rCf48 on the p38 Mapk, Erk1/2 and Jnk1/2 signaling pathways in NKR49F cells. *Quantification of phosphorylation of p38*

Mapk, Erk1/2 and Jnk1/2 signaling pathways on western blotting at 30 min after stimulation of NRK49F cells with rCf48± TGF-β1, TNF-α, or angiotensin II (ATII). One-way ANOVA with Tukey's multiple comparisons test. ns, $p>0.05$.

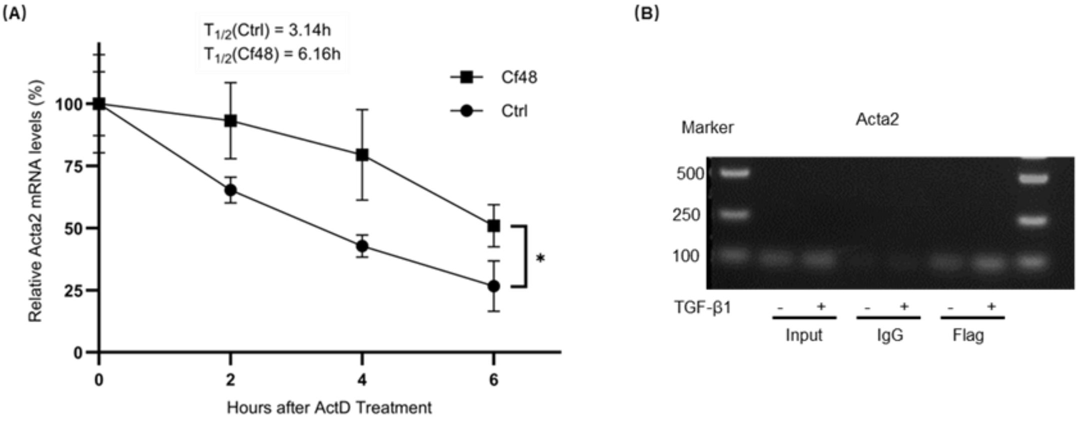

Supplementary Figure S16. Cf48 increased the half-life of Acta2 mRNA. *(A) Renal fibroblast NRK49F cells were stimulated with TGF-β1 for 6 h, then actinomycin D was added and decay of Acta2 mRNA levels in the presence or absence of rCf48 was measured by RT-qPCR. Unpaired student's t-test, *P<0.05. (B) The interaction between Cf48 and Acta2 mRNA was detected by RIP-RT-qPCR. End-products of RIP-RT-qPCR Acta2 was visualized using agarose gel electrophoresis.*

(A)

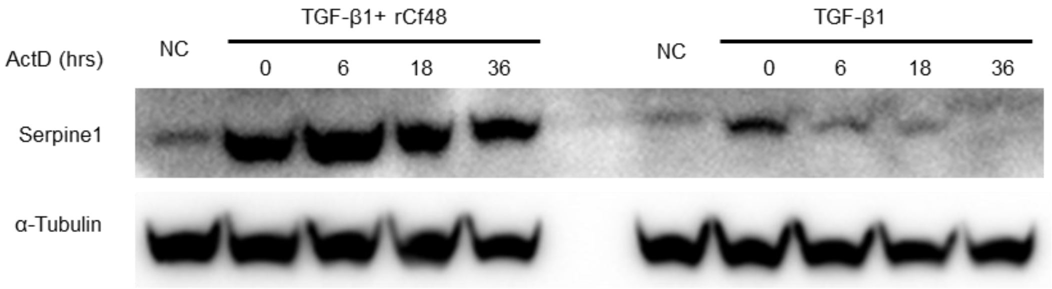

(B)

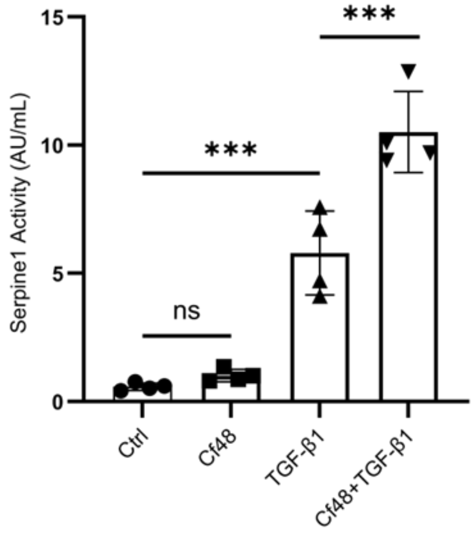

Supplementary Figure S17. Cf48 enhanced TGF-β1-induced upregulation of Serpine1 protein and activity in renal fibroblasts. *NRK49F cells were stimulated with recombinant TGF-β1 (10ng/ml) with or without flag-tagged rCf48 (100ng/ml) for 48hrs, then actinomycin D (ActD) was added in the presence or absence of rCf48 for various periods of time. (A) Western blot shows Serpine1 protein levels at different times after ActD addition. (B) Rat renal fibroblast NRK49F cells were stimulated with or without recombinant TGF-β1/Cf48 for 24 hrs and then Serpine1 activity was measured by Chromogenic Activity Assay kit. Data were expressed as mean ± SD. One-way ANOVA with Tukey's multiple comparisons test. ns, not significant; ***P<0.001.*

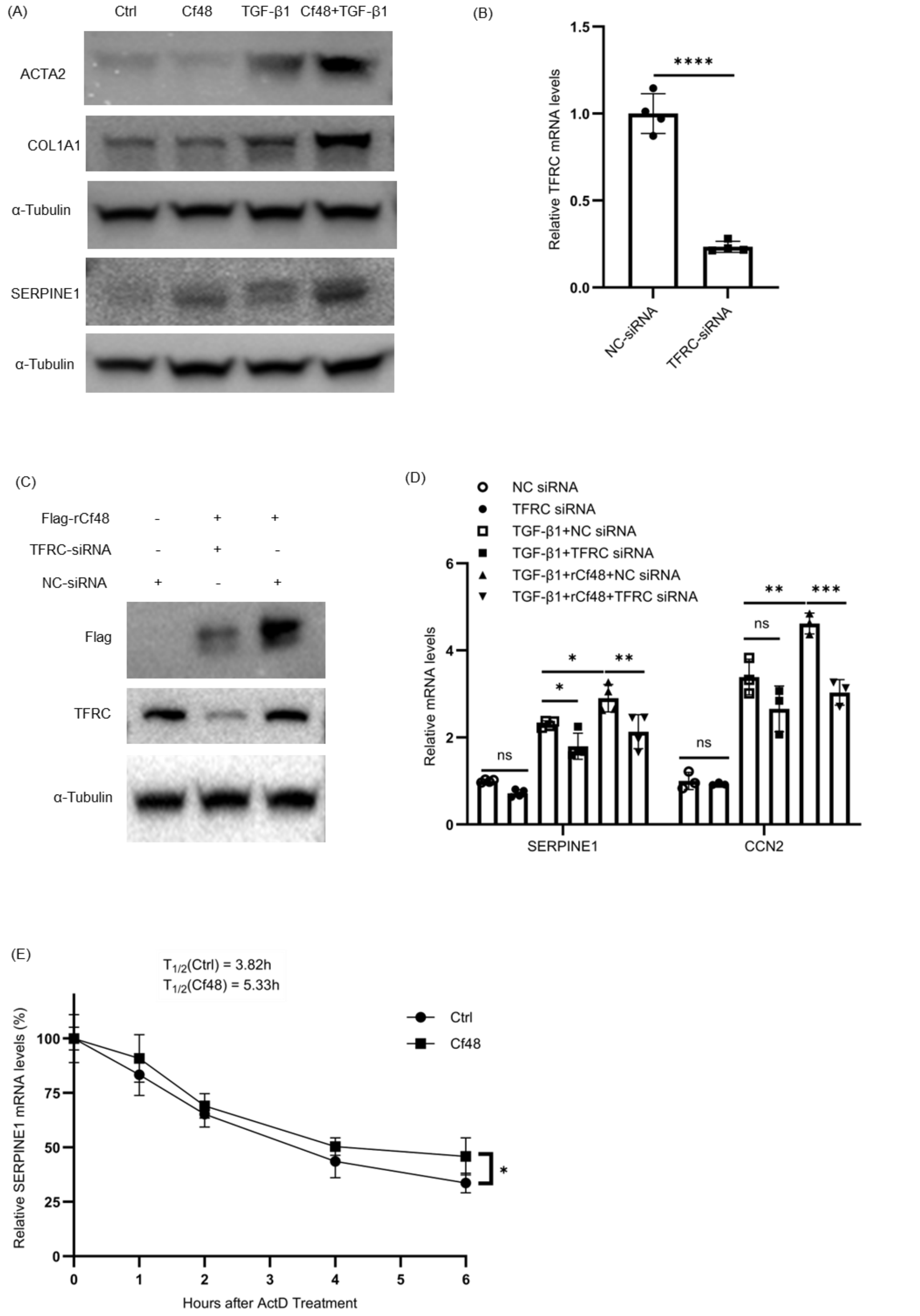

Supplementary Figure S18. Cf48 enhanced the TGF-β1-induced fibrotic response in primary human renal fibroblasts. *(A) Western blot showing ACTA2, COL1A1 and SERPINE1 in primary human renal fibroblasts after treatment with recombinant Cf48*

*or with Cf48 + TGF-β1 for 48 hrs. (B) Primary human renal fibroblasts were treated with control siRNA (NC siRNA) or TFRC siRNA for 24hrs and then TFRC mRNA levels determined by RT-qPCR. Unpaired student's t-test; ****P<0.0001. (C) Primary human renal fibroblasts were treated with NC-siRNA or TFRC-siRNA for 24 hrs, and then treated with or without Flag-tagged Cf48 for 2hrs. Western blot of cell lysates show cellular uptake of Flag-tagged Cf48 which is substantially reduced by knockdown of TFRC expression. (D) Primary human renal fibroblasts were treated with NC-siRNA or TFRC-siRNA for 24 hrs then treated with TGF-β1 or TGF-β1 + Flag-tagged Cf48 for 6hrs. SERPINE1 and CCN2 mRNA levels were assessed by RT-qPCR. One-way ANOVA with Tukey's multiple comparisons test: NS, not significant; *P<0.05; **P<0.01; ***P<0.001. (E) Primary human renal fibroblasts were stimulated with TGF-β1 for 6 h, then actinomycin D was added and decay of PAI-1 mRNA levels in the presence or absence of rCf48 was measured by RT-qPCR. Unpaired student's t-test, *P<0.05.*

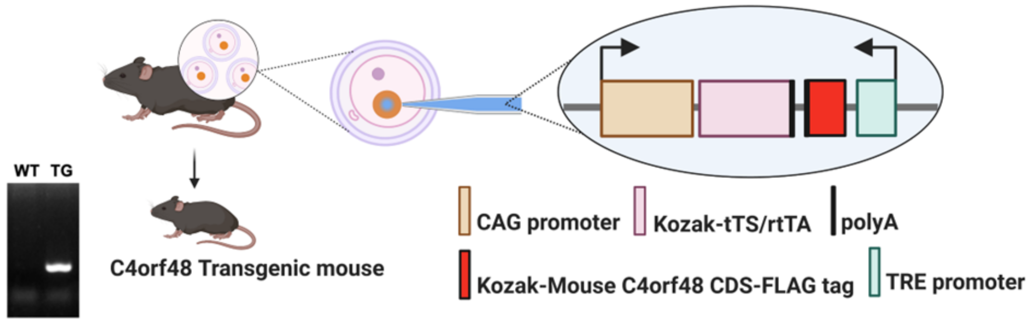

Supplementary Figure S19. *Schematic diagram outlining the construct used for generating the C4orf48 transgenic mouse line.*

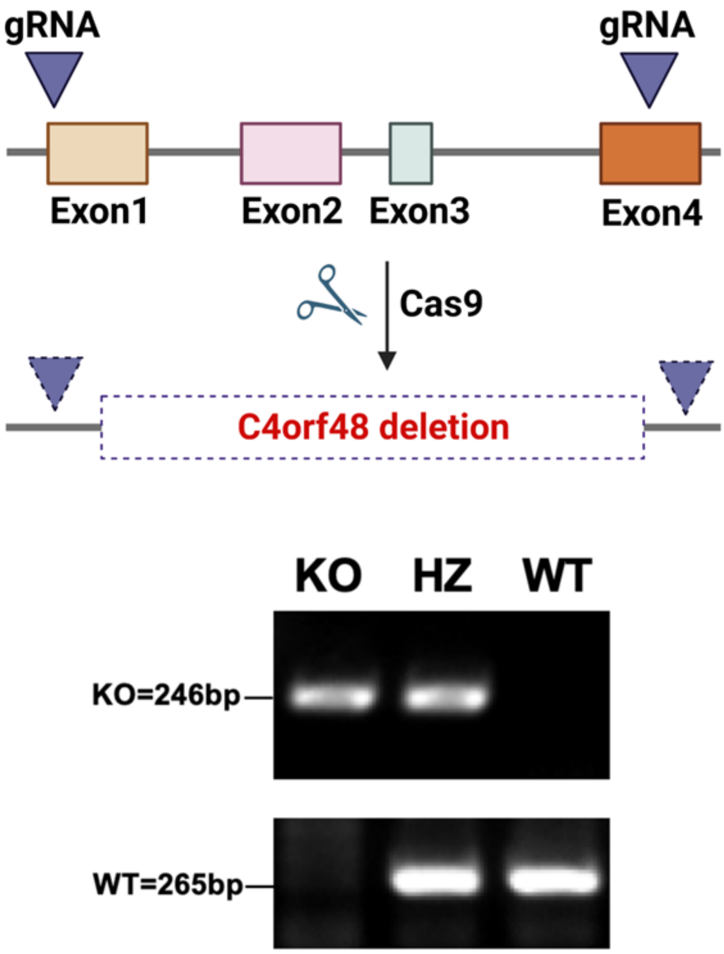

Supplementary Figure S20. Generation of the C4orf48 gene knockout mouse line.

Schematic diagram of the constructs used for the C4orf48 gene deletion, and PCR-based genomic analysis of the knockout (KO) and wild type (WT) alleles in homozygous knockout, heterozygous knockout and wild type mice.

Supplementary Materials and Methods

Analysis of Cf48 and ACTA2 expression in kidney biopsies

Indirect immunofluorescence staining for Cf48 was performed in formalin-fixed paraffin sections of renal biopsies using rabbit monoclonal anti-Cf48 antibody (ab185315, Abcam, USA) followed by Alexa Fluor 488 goat anti-rabbit IgG (A32731, Life Technology, USA). The area of staining for Cf48 and ACTA2-cy3 (C6198, Sigma-Aldrich, USA) was quantified in 10 consecutive high power fields (x400) fields of the tubulointerstitium, avoiding medium and large vessels, on blinded slides using the Image J program (http://www.imagej.nih.gov/ij/) as previously reported (1-3).

Mouse models of CKD

Diabetes was induced in 8-week-old male mice by 5 daily intraperitoneal injections of 55 mg/kg streptozotocin (STZ, S0130, Sigma-Aldrich, USA) in 0.1 mmol/L sodium citrate buffer (pH 4.5). Fasting blood glucose levels were measured at 2, 4, 6, 8, 10 and 12 weeks after STZ injection using a Glucose Meter (Sinocare, Changsha, Hunan, China) after deprivation of food for 6 h. Diabetes was defined as sustained fasting blood glucose above 16 mmol/L. The HbA1C level was measured in plasma samples taken at the time of killing. Spot urine was collected as time indicated or urine samples (24 h in a metabolic cage) were collected prior to killing mice.

Folic acid nephropathy (FAN) was induced in 8-12 weeks old male mice by an intraperitoneal injection of 250 mg/kg folic acid (F8758, Sigma-Aldrich, USA) in 0.3 mol/L

sodium bicarbonate buffer (1). A blood sample was taken on day 2 to measure renal function (assess AKI). Mice were killed 28 days after folic acid injection.

Renal interstitial fibrosis was induced by unilateral ureteral obstruction (UUO) surgery as previously described (2). Mice were killed at 7 days after UUO surgery. The blood and kidneys were collected for analysis.

Ischemia Reperfusion Injury (IRI)

Bilateral IRI was induced in 8-12 weeks old C57BL/6J mice. Briefly, after anesthesia, mice were transferred to a heat pad and the body temperature was maintained at 36.5-37.5°C throughout the procedure. Ischemia was induced bilaterally by clamping the renal pedicles with the atraumatic vascular clamps for 15 minutes. The clamps were removed at time completion and kidneys returned to the peritoneal cavity. Mice were killed 28 days after operation and the blood and kidneys were collected for analysis. Serum levels of Cf48 in mice were measured by ELISA (abx555467, Abbexa, UK) in sham or IRI animal model.

Induction of Cf48 overexpression in mice

7-week-old Cf48 Transgenic (Tg) and Cf48 wild type (WT) mice received a 2 mg/mL doxycycline (HY-N0565, MedChemExpress, China) in 5% sucrose solution as drinking water. We changed doxycycline-enriched water every 2 days and for a total duration of 7 consecutive days to induce the Cf48 expression in Cf48 Transgenic mice. Diabetes or folic acid nephropathy (FAN) was induced after the doxycycline-enriched water was supplied.

To keep Cf48 expression, mice were treated with doxycycline-enriched water until the mice were killed at the end of the experiment. The control mice were also treated with doxycycline-enriched water.

Locked nucleic acid (LNA) oligonucleotide treatment in mouse CKD models

Mouse Cf48 LNA and control LNA were designed by and purchased from QIAGEN (Hilden, Germany). LNA sequences are as follows: Cf48 LNA1 5'-CGCTGCATGAATTCAA-3'; Cf48 LNA2 5'-CCGTAGGTCCTGAAGG-3'; negative control-LNA (CTL LNA) 5'-AACACGTCTATACGC-3'. A preliminary experiment was performed to determine the potency of Cf48 LNA in the UUO model. CTL LNA (10 mg/kg), Cf48 LNA1 (10 mg/kg), Cf48 LNA2 (10 mg/kg) or Cf48 LNA1+ Cf48 LNA2 (5 mg/kg each) were administered to 8-week-old C57BL/6J mice on days 1 and 4 after UUO surgery and then killed on day 7 (n=3 mice/group). Subsequent studies used a combination of 5 mg/kg of each of Cf48 LNA1 + Cf48 LNA2 (referred to as Cf48 LNA) in the UUO model. In addition, diabetes was induced in *Nos3*$^{-/-}$ mice by 5 daily STZ injections. Two weeks after STZ injections, Cf48 LNA or CTL LNA (in PBS) was given once weekly by intraperitoneally injection for 6 weeks. Mice were killed at 8 weeks for analysis.

All animal studies were approved by the Sun Yat-Sen University Institutional Animal Care and Use Committee (No. SYSU-IACUC-2022-000134, -000361, -000943) and Guangdong Medical University Institutional Animal Care and Use Committee (No. GDY2204005).

Cell culture and treatments

Normal rat kidney fibroblast cells (NRK49F, CRL-1570, ATCC, USA), rat renal proximal tubule cells (NRK52E, CRL-1571, ATCC, USA) and human embryonic kidney cells (293T, CRL-3216, ATCC, USA) were cultured in DMEM mixed 1:1 (vol./vol.) with F12 medium (Life Technologies) and 10% FBS (Gibco). Human primary kidney fibroblasts (iCell Bioscience Inc, Shanghai, China) were maintained in primary fibroblast medium (PriMed-iCell-003, iCell Bioscience Inc, Shanghai, China) and 10% FBS (Gibco). Cells were cultured at 37 °C in 5% CO_2 and grown to approximately 60~80% confluence, transduced with a retroviral vector or expressing plasmid (VectorBuilder Inc, Guangzhou, China), and stimulated with or without recombinant Cf48 (rCf48) ± TGF-β1 (R&D, USA), TNF-α (R&D, USA), Angiotensin II (Sigma-Aldrich, USA) or $CoCl_2$ (Sigma-Aldrich, USA), rat Tfrc or human TFRC siRNA or NC siRNA (Genebiogist, Shanghai, China) in different experiments.

Histopathology

Kidney histology was assessed on Periodic Acid–Schiff (PAS, Sigma-Aldrich, USA) stained sections of formalin-fixed, paraffin-embedded tissue. Collagen deposition in renal tissue was evaluated by Masson's trichrome staining (Sigma-Aldrich, USA), with the area of blue collagen staining quantified in ten consecutive cortical fields (x200), excluding medium and large vessels, using ImageJ. Analysis was performed on blinded slides.

Immunohistochemistry and immunofluorescence staining

To detect macrophages, tissue sections were incubated with the F4/80 antibody (#70076, Cell Signaling Technology, USA), followed by Dako EnVision+ System-HRP Labelled Polymer Antibody (Dako, Carpinteria, CA, USA) and developed with diaminobenzidine (DAB) to

produce a brown color. Ten consecutive high power (x400) cortical fields, excluding glomeruli, were taken and F4/80-stained macrophages annotated and counted as cells per high field as previously reported (1). To detect Cf48 expression and ACTA2 expression, tissue sections were incubated with primary antibodies against Cf48 (ab185315, Abcam, USA) and ACTA2 (A5228, Sigma-Aldrich, USA), respectively, followed by 1h incubation with HRP Labelled Secondary Antibody and developed with DAB to produce a brown color. Analysis was performed on blinded slides.

For immunofluorescence staining, tissue sections or chamber slides (Lab-Tek II Chamber Slide System; Thermo Fisher Scientific, Waltham, MA) were incubated with primary antibodies against Cf48 (ab185315, Abcam, USA), Collagen IV (1340-01, SouthernBiotech, USA), ACTA2 (A5228, Sigma-Aldrich, USA), PECAM-1 (CD31, ab9498, Abcam, USA), Fibronectin (Fn1, ab2413, Abcam, USA), TFRC (ab214039, Abcam, USA) and Flag (ab205606, Abcam, USA) overnight, followed by 1h incubation with Alexa Fluor Secondary Antibody (Life Technology, USA).

For double labelling, tissue sections were incubated with primary antibody rabbit anti-Cf48, followed by goat anti-rabbit Alexa Fluor 555 Secondary Antibody, then LTL-conjugated with Alexa Fluor 488 (FL-1321-2, Vectorlabs, USA), VCAM1 conjugated with Alexa Fluor 488 (bs-0920R-AF488, bioss, China), or UMOD- FITC (LS-B3105, Lsbio, USA). Tissue sections were incubated with goat anti-NKCC2 (ab240542, Abcam, USA), followed by chicken anti-goat Alexa Fluor 488, then rabbit anti-Cf48 antibody followed by goat anti-rabbit Alexa Fluor 555. For triple labelling, issue sections were incubated with primary antibody rabbit anti-Flag, followed by Alexa Fluor 488 Secondary Antibody, followed by PAX8-AF647 (ab215953, Abcam, USA) and ACTA2-cy3 (C6198, Sigma-Aldrich, USA).Sections were counterstained with DAPI (4, 6-diamidino-2-phenylindole; Sigma-Aldrich, USA) to

visualize nuclei. For cell immunofluorescent staining, NRK49F cells were seeded into a chamber slide and cultured with or without flag-tagged rCf48 for 2 h. Fluorescence images were captured using a Zeiss LSM880 confocal microscope (Oberkochen, Germany).

Renal function and albuminuria

Serum Cystatin C levels were measured with a commercially available ELISA kit (ab201280, Abcam, UK). Serum creatinine and blood urea nitrogen (BUN) were analyzed using an autoanalyzer (Hitachi 717 Chemistry Analyzer, Tokyo, Japan). Urine samples were collected each week after 2 weeks of STZ treatment in DN mice. Urinary albumin levels were measured with a Mouse Albumin ELISA Kit (ab207620, Abcam, UK), and urine creatinine levels were measured using a Creatinine Assay Kit (KGE005, R&D, USA) according to the manufacturer's instructions. Results are expressed as the urine albumin-to-creatinine ratio (μg albumin/mg creatinine).

Cf48 conditioned medium.

Expression plasmids for mouse Cf48 cDNA (pMSCV-Cf48) and control plasmid (pMSCV) were transfected into 293T cells using Lipofectamine 3000 (Invitrogen, USA). Cells were then incubated with serum-free Expression Medium (Invitrogen, USA) for 48 h to generate conditioned medium. The conditioned media was collected, filtered by 0.2μm filter and used to stimulate normal rat kidney fibroblast cells (NRK-49F, ATCC, USA) with or without 2 ng/ml TGF-β1 for 48 h to determine the fibrotic response.

Synthesis of recombinant Cf48 protein.

The sequence of the mouse Cf48 secreted peptide was cloned into the Fc-Flag pATX1 expression vector (see Supplementary Table S12), and the Fc-Flag-Cf48-pATX1 plasmid was transfected into Chinese Hamster Ovary (CHO) cells. Fc-Flag-Cf48 protein was purified from 2 L of CHO conditioned culture media. The free FC-tag was cleaved with Enterokinase and purification of recombinant Flag-Cf48 protein was confirmed by SDS-PAGE (Figure 7A).

Real-time quantitative PCR (RT-qPCR)

Total RNA was isolated from kidney tissues or cells using Trizol (Invitrogen, CA) according to the manufacturer's instructions. Real-time PCR was performed with the SYBR Green Supermix using the CFX96 PCR System (Bio-Rad, CA) or the LightCycle480 PCR System (Roche, Switzerland). Primer sequences are shown in Supplementary Table S13. The relative mRNA levels of target genes were normalized to mRNA levels of the housekeeping gene β-Actin.

Western blotting

Kidney cortex or cultured cells were lysed in RIPA buffer. Protein samples were separated by SDS-PAGE and transferred to polyvinylidene difluoride membranes. The membrane was incubated with the primary antibody at 4°C overnight followed by horseradish-peroxidase (HRP)-conjugated secondary antibody (anti-rabbit IgG, #7074 or anti-mouse IgG, #7076) and detected by Amersham ECL Advance Western Blotting Detection Kit (GE Healthcare Life Sciences, Code: RPN2108). Primary antibodies used were: Acta2 (A5228, Sigma-Aldrich, USA), Col1a1 (PB0981, Boster, China) and Serpine1 (NBP1-19773, Novus, USA); Cf48 (ab185315), Fibronectin (Fn1, ab2413), Smad3 (ab40854), Flag (ab205606), Tfrc

(ab214039), Serpine1 (ab222754) and Ccn2 (ab209780) were from Abcam (UK); phospho-Smad3 (#9520), p38 MAPK (#54470), phospho-p38 MAPK (#4511), phospho-Erk1/2 (#4370), Erk1/2 (#4695), phospho-Jnk (#9255), Jnk (#9252), E-cadherin (#14472), α-Tubulin (HRP conjugated, #12351) and GAPDH (HRP conjugated, #8884) were from Cell Signaling Technology (USA).

SBE4-luciferase activity assay

One day after SBE4-luciferase plasmid was transfected into 293T cells by Lipofectamine 3000, 293T cells were treated with or without rCf48 ± TGF-β1 for 15 h. Then, cells were harvested and SBE4-luciferase assay was performed according to the instruction of the luciferase assay kit (Promega).

Immunoprecipitation Mass spectrometry (IP/MS)

To explore the Cf48-binding proteins, we performed immunoprecipitation/mass spectrometry (IP/MS) to identify the receptors that could interact with Cf48. 293T cells were transfected with flag-tagged Cf48 plasmids and the cell lysates from Cf48-overexpressing 293T cells were collected and added rabbit anti-Flag (ab205606, Abcam, UK) antibody with gentle rocking overnight at 4°C. The immune complex was immunoprecipitated using protein A/G agarose beads (Santa Cruz Biotechnology) then washed three times with 0.1 ml of lysis buffer. The bound proteins were separated by SDS-PAGE, collected and analyzed by MS to identify proteins that interacted with Cf48. The list of proteins that interacted with Cf48 is presented in Supplementary Table S6.

Immunoprecipitation/Western blotting (IP/WB)

Two days after flag-tagged Cf48 and HA-tagged TFRC plasmids were transfected into 293T cells by Lipofectamine 3000, cells were harvested and lysed in 0.4 ml RIPA lysis buffer. Cell lysates (0.5-1 mg) were added rabbit anti-Flag (Abcam, Cat. No. ab205606) or rabbit control IgG (Cell Signaling Technology, Cat. No. 3900) with gentle rocking overnight at 4°C then immune complex was immunoprecipitated using protein A/G agarose beads (Santa Cruz Biotechnology). In western blotting, protein samples were separated by SDS-PAGE and transferred to polyvinylidene difluoride membranes. The membrane was incubated with the primary antibody TFRC (Abcam, ab214039) at 4°C overnight followed by horseradish-peroxidase (HRP)-conjugated secondary antibody.

Surface plasmon resonance (SPR) Assay

SPR assay was performed using OpenSPR™ Instrument with OpenSPR™ COOH Sensor Chip and Amine Coupling Kit according to the standard procedure found in the OpenSPR™ manual.

Serpine1 mRNA and Acta2 mRNA half-life experiments

For Serpine1 mRNA/Acta2 mRNA half-life experiments, actinomycin D (ActD, Sigma-Aldrich, Shanghai, China) was used to block transcription. NRK49F cells or primary human kidney fibroblasts were stimulated with recombinant TGF-β1 (10ng/ml) with or without flag-tagged rCf48 (100ng/ml) for 6 h, and then treated with ActD (10μg/ml) with or without rCf48 to shut off transcription for different time points. The relative Serpine1 mRNA/Acta2 mRNA

levels (%) were normalized to the level of Serpine1 mRNA/Acta2 mRNA expression before treatment with ActD.

Ribo-seq

Tissues were immediately frozen in liquid nitrogen and ground to power in liquid nitrogen with a mortar and pestle, then dissolved in 400μL of lysis buffer containing 10 mM Tris-HCl (pH 7.4), 5 mM MgCl 2, 100 mM KCl, 1% Triton X-100, 2 mM DTT, 100 μg/mL Cycloheximide, 500 U/mL RNasin Plus and cOmplete EDTA-free protease inhibitor (1 mini tablet per 10 mL). Ribosome footprints (RFs) were prepared by adding 7.5μL of RNase I and 5μL of DNase I to 300μL of tissue lysate, and incubatinge for 45 min at room temperature with gentle mixing on a Nutator mixer. Nuclease digestion was stopped by adding 10μL of SUPERase·In RNase inhibitor. Digested RFs (100μL) were added to size exclusion columns (illustra MicroSpin S-400 HR Columns; GE Healthcare; catalog no. 27-5140-01) and centrifuged at 600g for 2 min and eluate collected. Next, 10μL 10% (wt/vol) SDS was added to the elution, and RFs with a size greater than 17nt isolated using the RNA Clean and Concentrator-25 kit (Zymo Research; R1017). rRNA was removed using DNA probes complementary to rRNA sequences, then RNase H and DNase I was used to digest the probes. RFs were purified using magnet beads (Vazyme) and Ribo-seq libraries constructed using the NEBNext® Multiple Small RNA Library Prep Set for Illumina® (catalog no. E7300S, E7300L). Briefly, adapters were added to both ends of RFs, followed by reverse transcription and PCR amplification. The 140-160bp size PCR products were enriched to generate a cDNA library and sequenced using Illumina HiSeqTM 2500 by Gene Denovo Biotechnology Co. (Guangzhou, China).

Ribo-seq data analysis

Reads filtering

Raw reads containing over 50% of low-quality bases or over 10% of N bases were removed. Adapter sequences were trimmed. Reads with length between 10-50bp were retained for subsequent analysis.

rRNA removal

Short reads alignment tool Bowtie2 was used for mapping reads to ribosome RNA (rRNA) database. The rRNA mapped reads will be removed. The remaining reads were further used in downstream analysis.

Reference genome alignment

The rRNA removed reads of each sample were mapped to reference genome by Bowtie2 allowing no mismatches. RFs were assigned to different genomic features (5'UTR, CDS, 3'UTR and others) based on the position of the 5' end of the alignment.

Ribosome density analysis

To monitor sequencing reliability, RFs density at different codon positions was calculated. Generally speaking, RFs have the highest density at the first base of codon.

Quantification of gene abundance

Reads number in the open reading frame of coding genes was calculated by software RiboTaper, and the gene expression level was normalized by using FPKM (Fragments Per Kilobase of transcript per Million mapped reads) method, and the formula is shown as follows:

$$FPKM=10^6C/(NL/10^3)$$

Given FPKM(A) to be the expression of gene A, C to be number of fragments mapped to gene A, N to be total number of fragments that mapped to reference genes, and L to be number of bases on gene A. The FPKM method is able to eliminate the influence of different gene lengths and sequencing data amount on the calculation of gene expression. Therefore, the calculated gene expression can be directly used for comparing the difference of gene expression among samples.

Sample relationship analysis

To evaluate the reliability of experimental results, the correlation coefficient between two replicas was calculated. Meanwhile, Principal component analysis (PCA) was performed with R package gmodels (http://www.r-project.org/) to reveal the structure/relationship of the samples/datas.

Differentially translated genes (DTGs) analysis

To identify differentially translated genes across sample groups, the edgeR package (http://www.r-project.org/) was used. Genes with a fold change ≥2 and a false discovery rate (FDR)<0.05 in a comparison were considered as significant DTGs. DTGs were then subjected to enrichment analysis of GO functions and KEGG pathways.

4D-DIA Proteomics

4D-DIA Proteomics was performed by PTM Biolab (Hangzhou, Zhejiang, China). Samples were ground into a powder in liquid nitrogen and four volumes of lysis buffer (8 M urea, 1% Protease Inhibitor Cocktail) was added to the powder, followed by sonication and centrifugation. The supernatant was collected, and trypsin digestion was performed. The sample was then fractionated by high pH reverse-phase HPLC using an Agilent 300Extend

C18 column (5 µm particles, 4.6 mm ID, 250 mm length). The iRT kit was added to all the samples according to manufacturer's instructions. The LC gradient was kept consistent with those in the spectral library building method. The separated peptides were analyzed in Q ExactiveTM HF-X (Thermo Fisher Scientific) with a nano-electrospray ion source. The full MS scan resolution was set to 120,000 for a scan range of 385–1200 m/z. The data acquisition was performed in DIA mode. Each cycle contains one full scan followed by 70 DIA MS/MS scans with a predefined precursor m/z range. The HCD fragmentation was performed at a normalized collision energy (NCE) of 27%. The fragments were detected in the Orbitrap at a resolution of 15,000. Fixed first mass was set as 200 m/z. Automatic gain control (AGC) target was set at 5E5. All DIA data were analyzed in Skyline (v 20.1.0). The FDR was estimated with the mProphet approach and set to 1%. Relative quantification of proteins was performed using MSstats package. Gene Ontology (GO) annotation proteome was derived from the UniProt-GOA database. Kyoto Encyclopedia of Genes and Genomes (KEGG) database was used to annotate protein pathway. (http://www.ebi.ac.uk/GOA/). Proteins were classified by GO annotation into three categories: biological process, cellular compartment and molecular function. For each category, a two-tailed Fisher's exact test was employed to test the enrichment of the differentially expressed protein against all identified proteins. The GO with a corrected P-value < 0.05 is considered significant.

RNA electrophoretic mobility shift assay

The Thermo Scientific Light Shift Chemiluminescent RNA EMSA Kit (Catalog number: 20158, Thermo Fisher Scientific Co, Shanghai, China) was used to detect the interactions between rCf48 and Serpine1 mRNA sequences, and between rCf48 and Acta2 mRNA sequences, according to the manufacturer's instructions.

RNA immunoprecipitation sequencing (RIP-seq)

NRK49F cells were treated with flag-tagged rCf48 (50ng/ml) for 2 hr. Cells (1x 10^7) were collected, lysed in freshly prepared RIP buffer and split into two fractions of 500 µL each (for Input and IP). Chromatin shearing was performed using a Dounce homogenizer with 15–20 strokes. Rabbit anti-Flag antibody (Abcam, Cat. No. ab205606) was used to perform immunoprecipitation. RNA that was bound to precipitated RBP was purified, and a cDNA library created via reverse transcription. High-throughput sequencing of the cDNA library was performed in NovaSeq 6000. After the RIP-Sequencing data pre-processing, genome mapping by Hisat2 (4), peak calling and annotation by feature Counts (5) and DESeq2 (6), we identified 3929 Cf48 targets (IP/Input > 1). Gene Ontology analysis was performed to reveal Cf48 molecular function (7-9).

RNA immunoprecipitation-Reverse Transcription-quantitative PCR (RIP-RT-qPCR)

The PureBinding RNA Immunoprecipitation Kit (Catalog number: P0102, Geneseed Biotech Co, Guangzhou, China) was used to confirm the interactions between Flag-Cf48 and Serpine1, Acta2 or Ccn2 mRNA sequences according to the manufacturer's instructions. NRK49F cells were transduced with retroviral vector PMSCV-Flag-Cf48-IRES-puro (puromycin resistance). Puromycin resistant NRK49F cells were selected with puromycin (Solarbio, 8µg/ml) and then cultured with or without recombinant 10 ng/ml TGF-β1 for 6 hr. Cells (1x 10^7) were collected, lysed in freshly prepared RIP buffer and split into three fractions (for Input, IgG and RIP). Rabbit anti-Flag antibody (Abcam, Cat. No. ab205606) was used to perform RIP and Rabbit IgG Control (CST, Cat. No.3900) was used for negative control RIP. RNA product was purified. Reverse transcription of RNA to DNA and real-time

quantitative PCR were performed to detect Serpine1, Acta2 and Ccn2 mRNA levels. Normalize each RIP RNA fractions' Ct value to the Input RNA fraction Ct value for the same qPCR Assay (ΔCt) to account for RNA sample preparation differences, and Fold enrichment above the sample control (IgG) was calculated as linear conversion of ΔΔCt: Fold enrichment = 2^ (-ΔΔCt [Flag RIP/IgG RIP]). Finally, end-products of RIP-RT-qPCR were revealed by agarose gel electrophoresis.

In situ hybridization

Expression of C4orf48 RNA in human renal biopsy samples was examined with in situ hybridization. Briefly, sections of paraffin-embedded kidney tissue were boiled in the retrieval solution for 10 minutes and digested with proteinase K (20 ug/ml) at 37℃ for 15min. After fixation in 4% paraformaldehyde, sections were hybridized with the Digoxin labeled C4orf48 probe at 52 °C overnight. After washing, tissues were incubated at 37 °C for 60min with rhodamine labeled Digoxin antibody (Roche, cat.11207750910) and counterstained with DAPI. The sequence of DNA to generate C4orf48 RNA probe was listed as follow: 5'-CCACTGGGCATTTATTCACACGGAGCAGCGT-3'.

Serpine1 activity

The activity of Serpine1 protein in NRK49F cell culture supernatant samples was examined with Serpine1 Chromogenic Activity Assay Kit (Abnova, China). Rat renal fibroblast NRK49F cells were stimulated with or without recombinant TGF-β1/Cf48 for 24 hrs and then Serpine1 activity was measured by Chromogenic Activity Assay kit, according to the manufacturer's instructions.

Mass spectrometry-based D-DIA proteomic quantification

To test for secretion of a bioactive peptide, 293T cells were transfected with a Cf48 overexpression plasmid, or empty control plasmid, and conditioned media was collected after 24hrs. Mass spectrometry-based D-DIA proteomic quantification (NKY GENEREADER, China) was used to detect molecules in the collected conditioned medium.

References:

1. Jiang M, et al. Combined Blockade of Smad3 and JNK Pathways Ameliorates Progressive Fibrosis in Folic Acid Nephropathy. *Front Pharmacol.* 2019;10:880.

2. Qu X, et al. The Smad3/Smad4/CDK9 complex promotes renal fibrosis in mice with unilateral ureteral obstruction. *Kidney Int*. 2015;88(6):1323-1335.

3. Li J, et al. Smad4 promotes diabetic nephropathy by modulating glycolysis and OXPHOS. *EMBO Rep*. 2020;21(2):e48781.

4. Kim D, et al. HISAT: a fast spliced aligner with low memory requirements. *Nat Methods*. 2015;12(4):357-360.

5. Liao Y, et al. featureCounts: an efficient general purpose program for assigning sequence reads to genomic features. *Bioinformatics*. 2014;30(7):923-930.

6. Love MI, et al. Moderated estimation of fold change and dispersion for RNA-seq data with DESeq2. *Genome biol*. 2014;15(12):550.

7. Ashburner M, et al. Gene ontology: tool for the unification of biology. *Nat Genet.* 2000;25(1):25-29.

8. The Gene Ontology Consortium. The gene ontology resource: 20 years and still GOing strong. *Nucleic Acids Res.* 2019;47(D1):D330-D338.

9. Kanehisa M, Goto S. KEGG: kyoto encyclopedia of genes and genomes. *Nucleic Acids Res.* 2000;28(1):27-30.
