# Supplementary material for: The secreted micropeptide C4orf48 enhances renal fibrosis via an RNA-binding mechanism
Source: J Clin Invest. 2024 Apr 16;134(10):e178392. doi: 10.1172/JCI178392 (PMC11093611; doi:10.1172/JCI178392)

Unedited blot and gel images for C4orf48 project

2024-03-24

Fig.1A

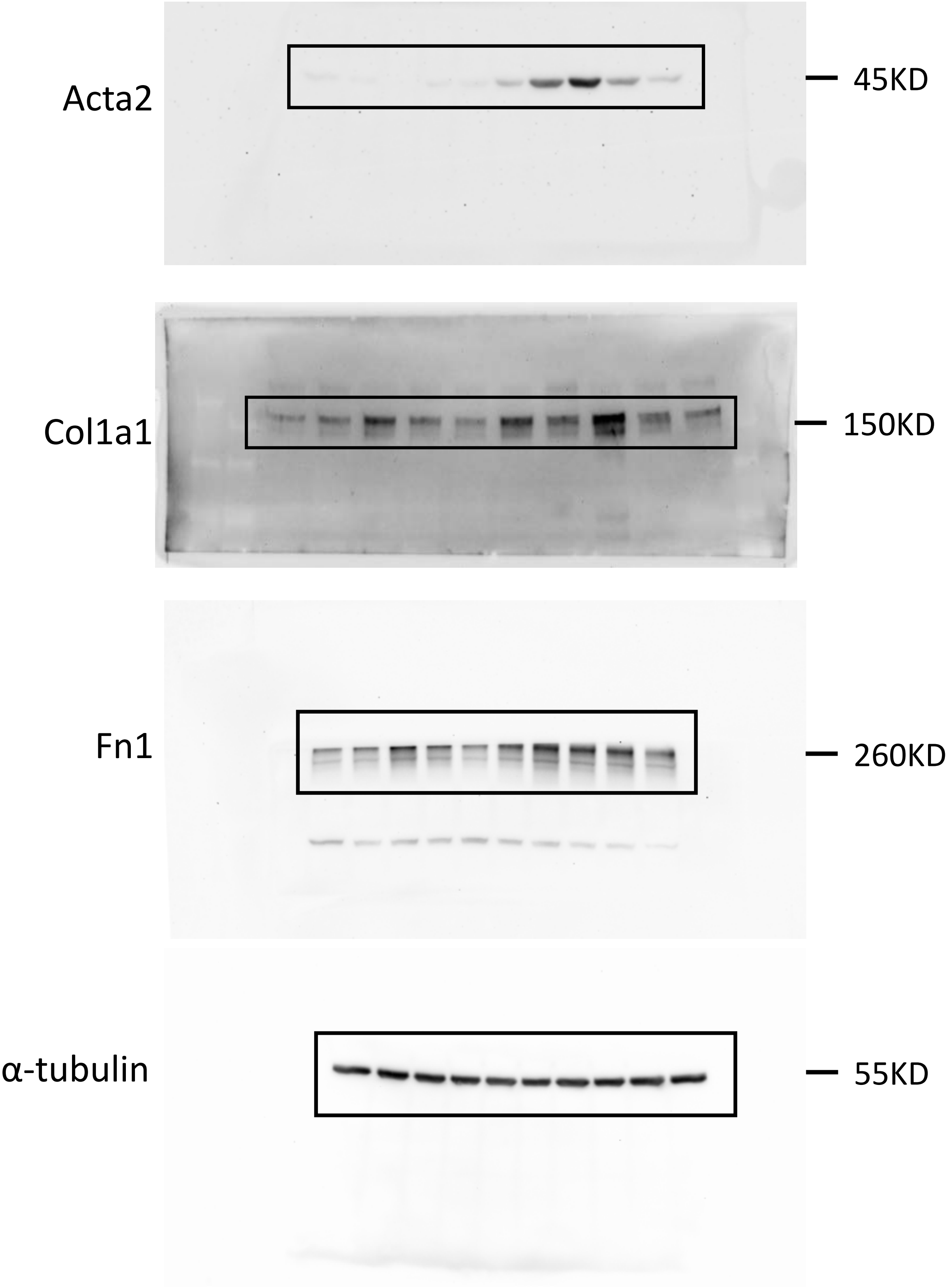

Fig.1C

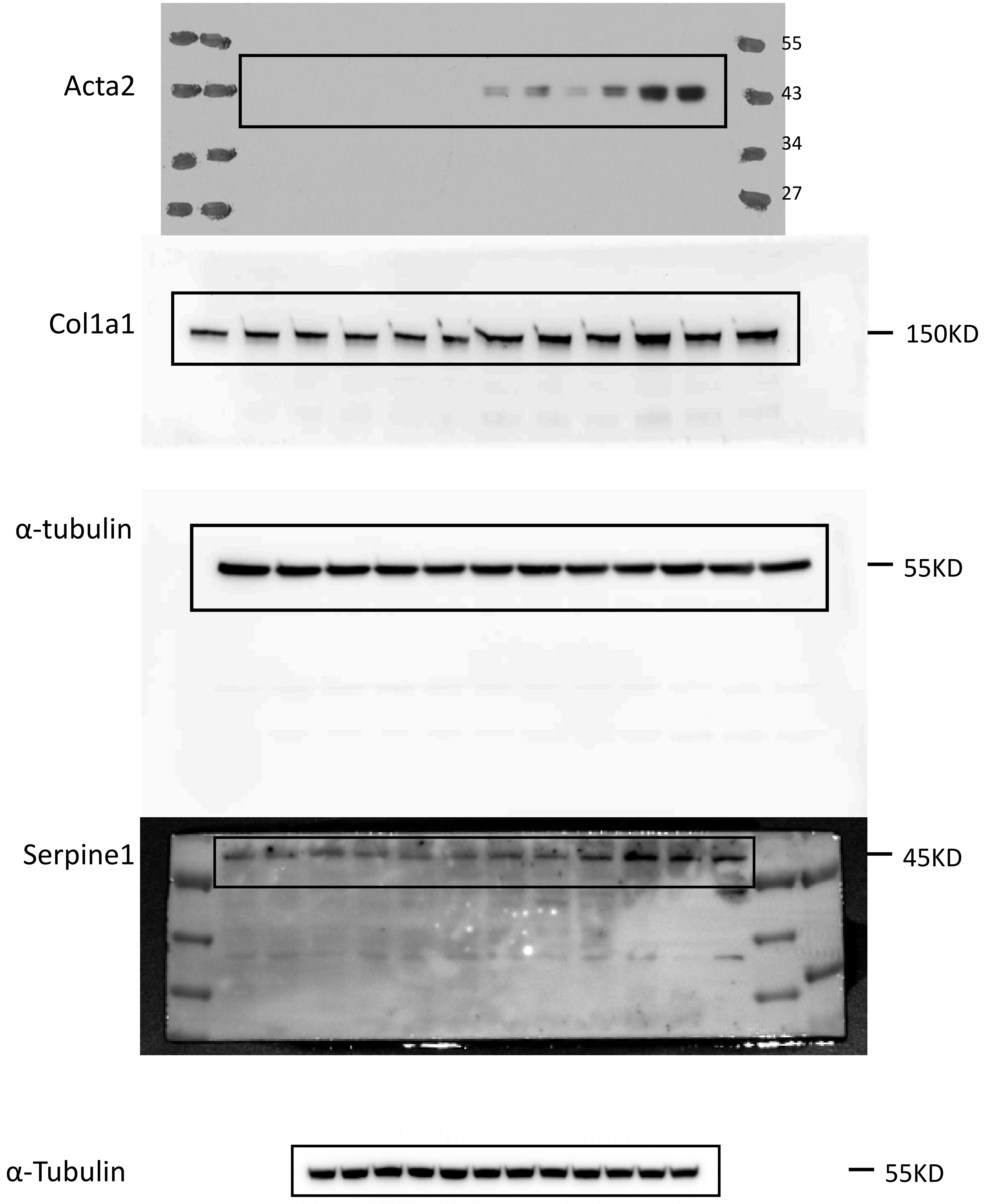

Acta2
55
43
34
27
Col1a1
150KD
α-tubulin
55KD
Serpine1
45KD
α-Tubulin
55KD

Fig.1F

Cf48

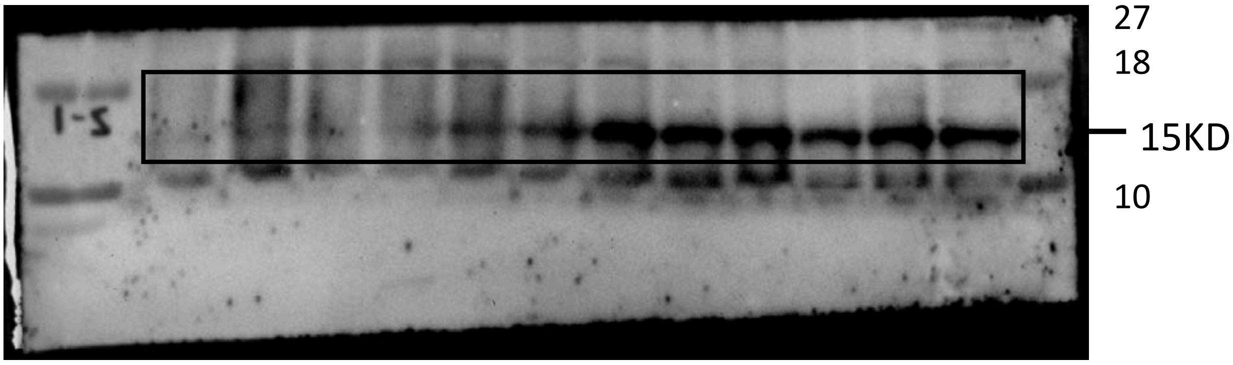

27
18
15KD
10

α-Tubulin

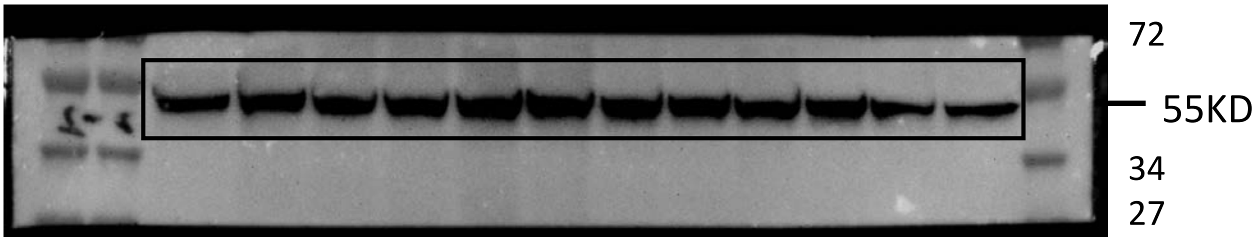

72
55KD
34
27

Fig.5H

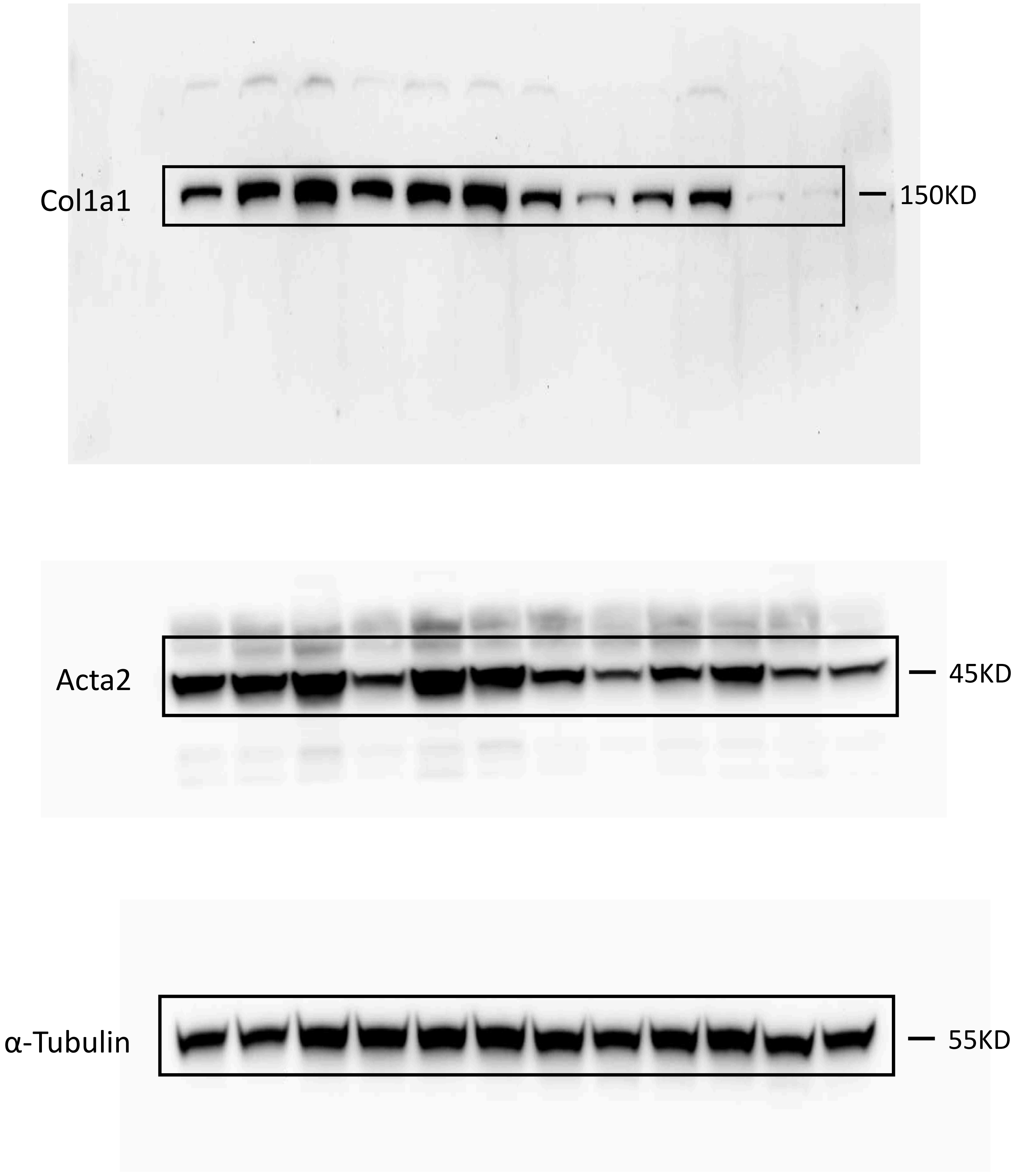

Fig.6A

Cf48

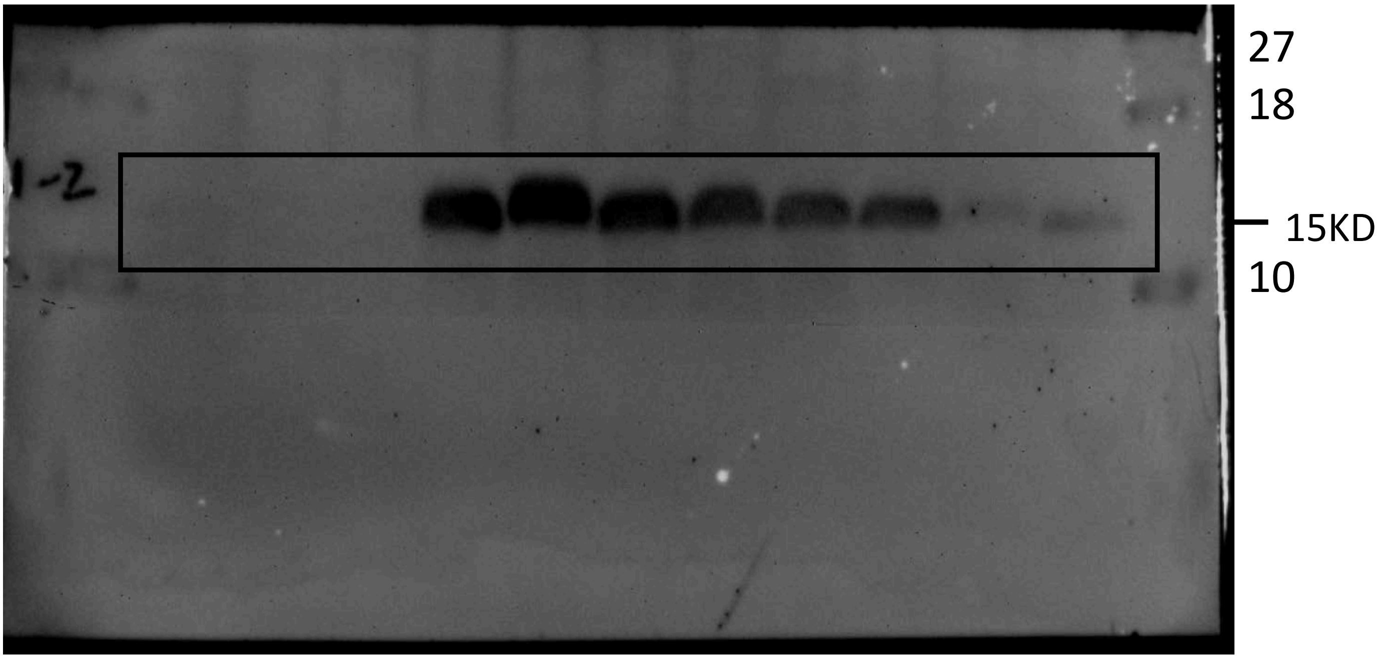

α-Tubulin

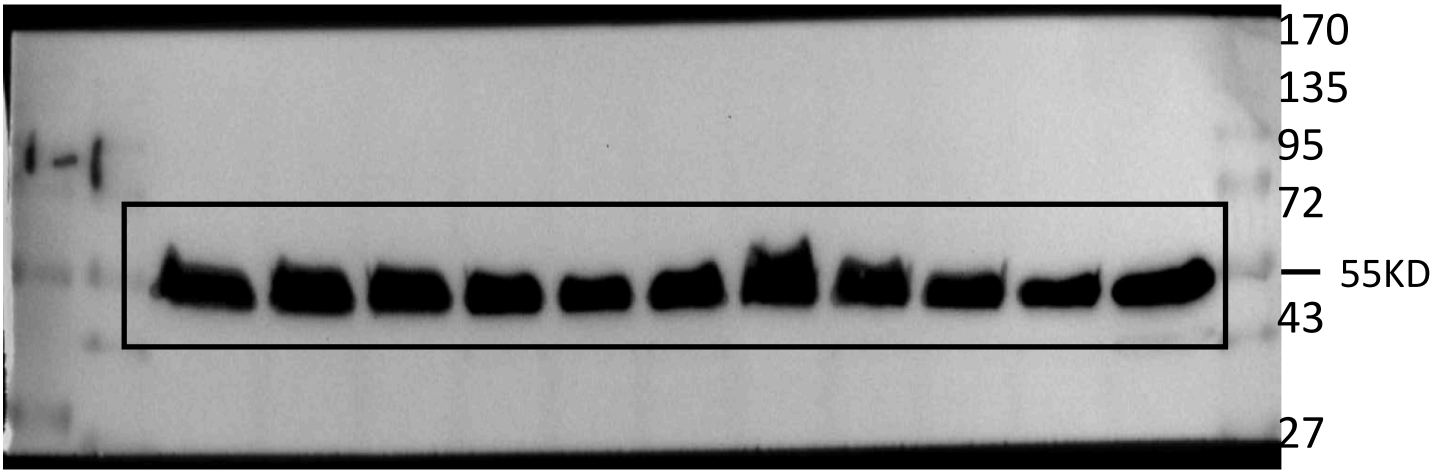

Fig.6B

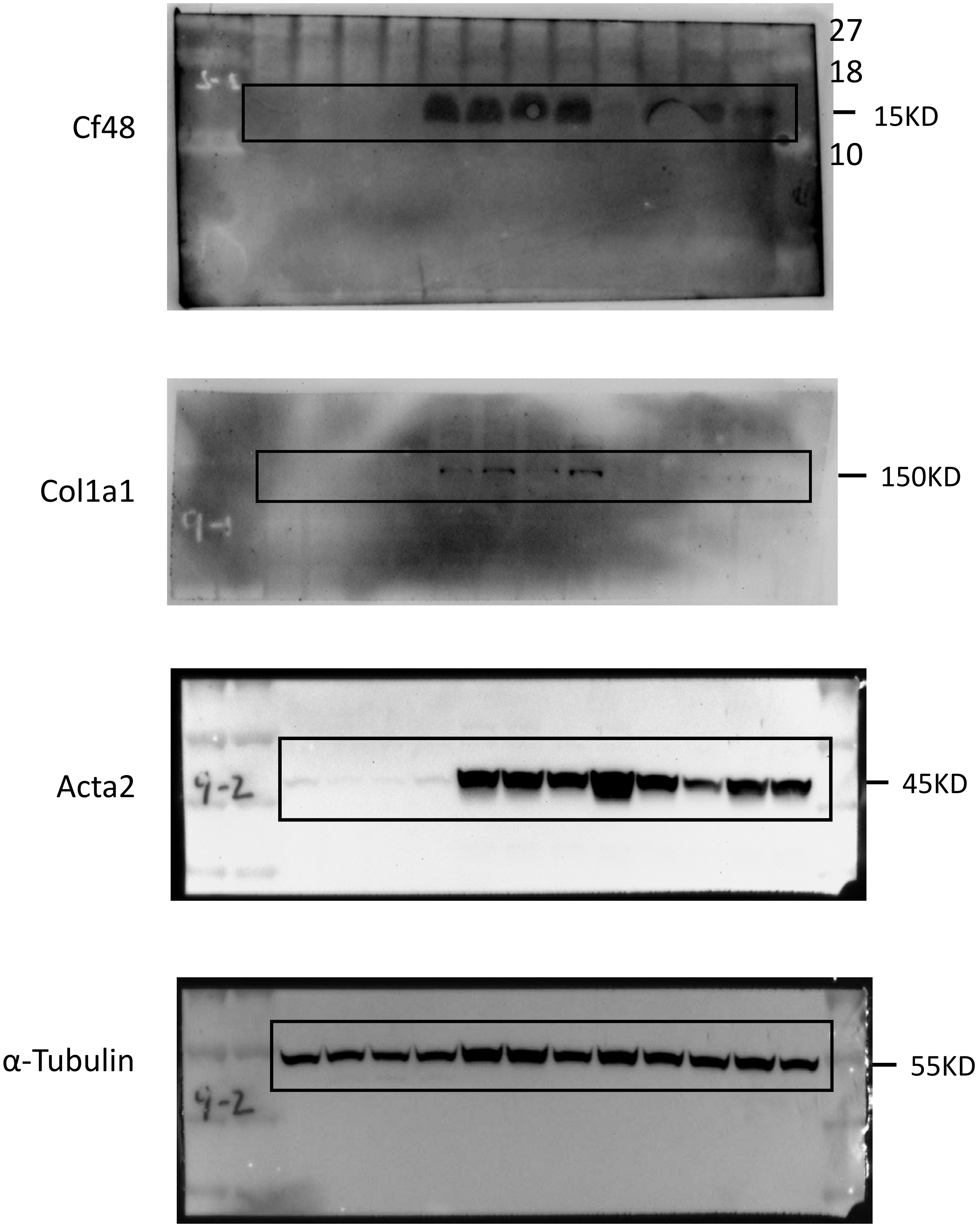

Fig.7B

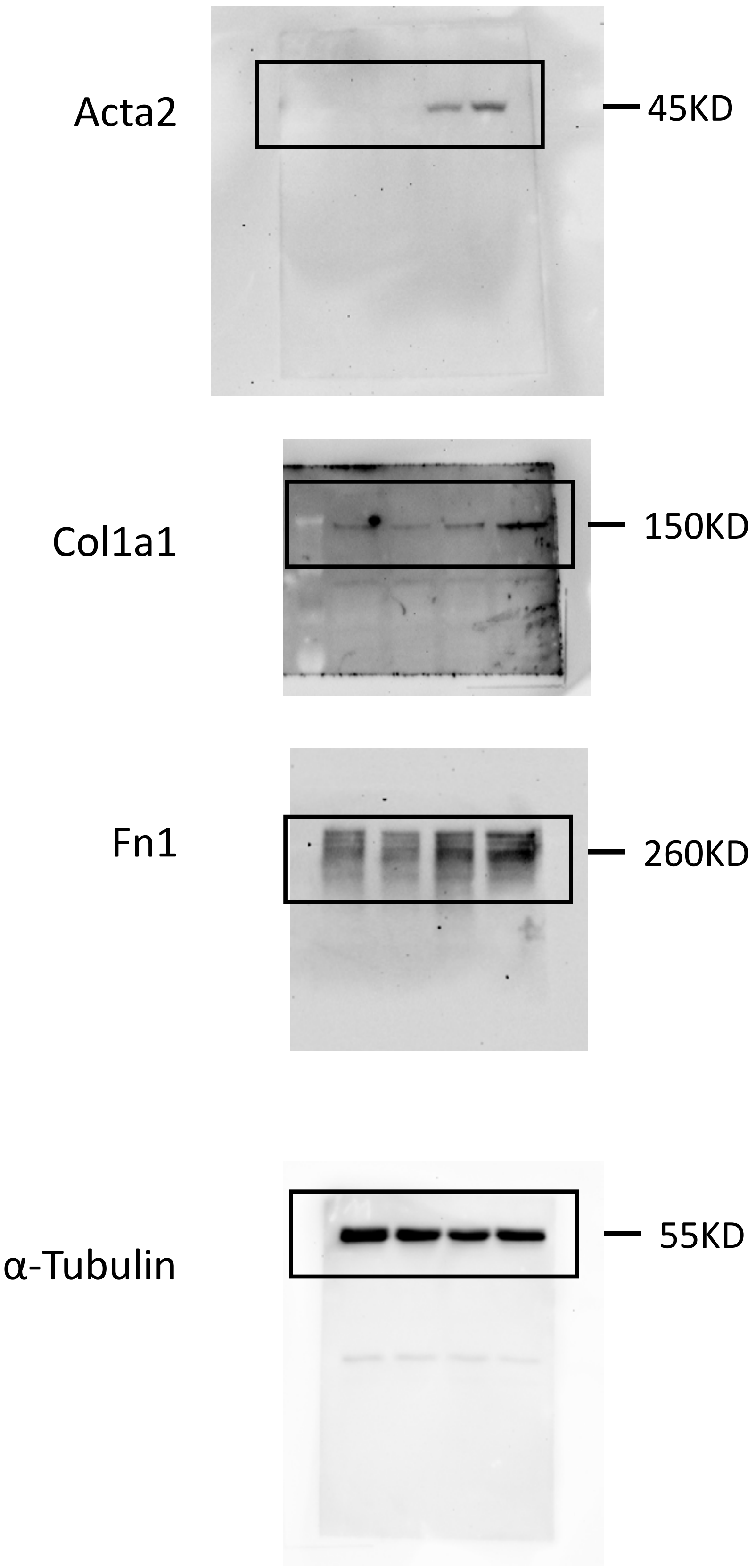

Fig.7C

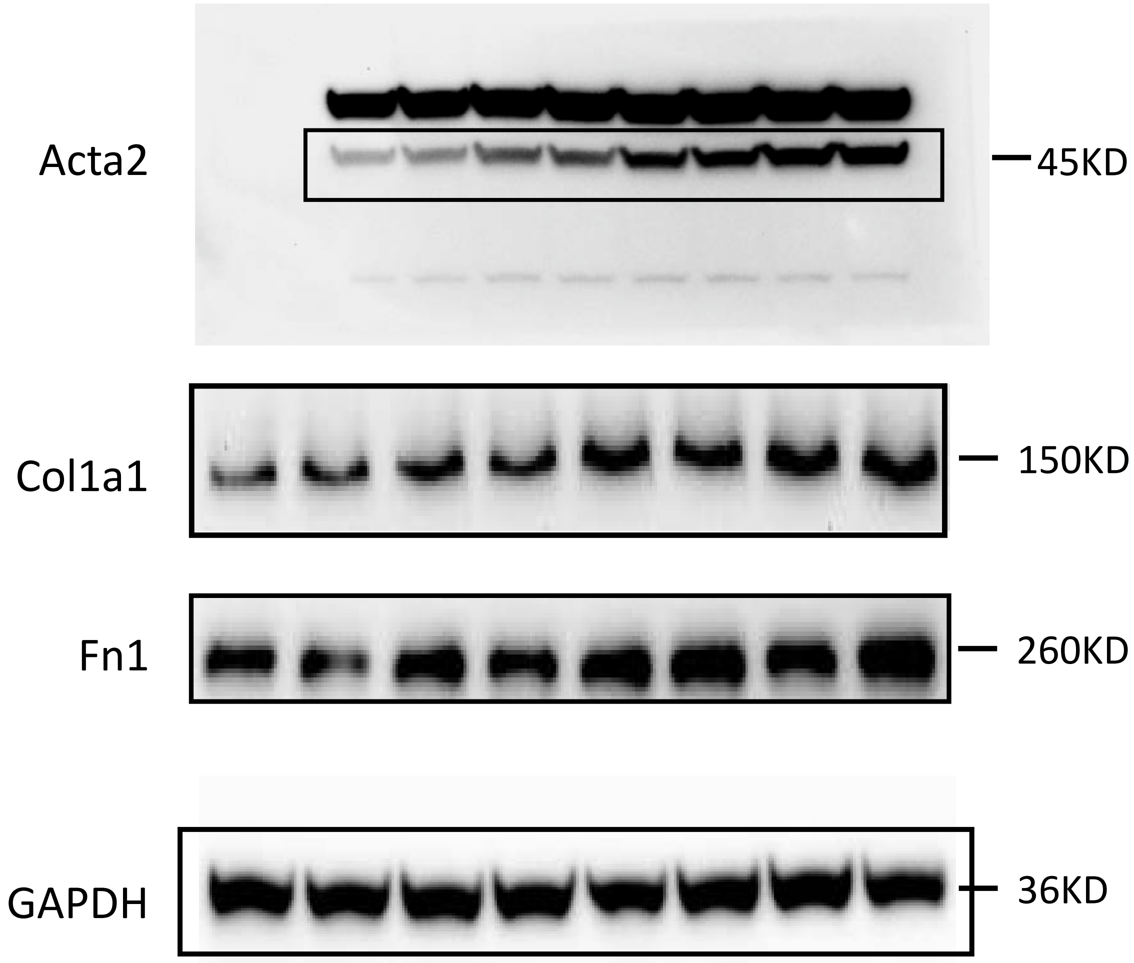

Fig.7D

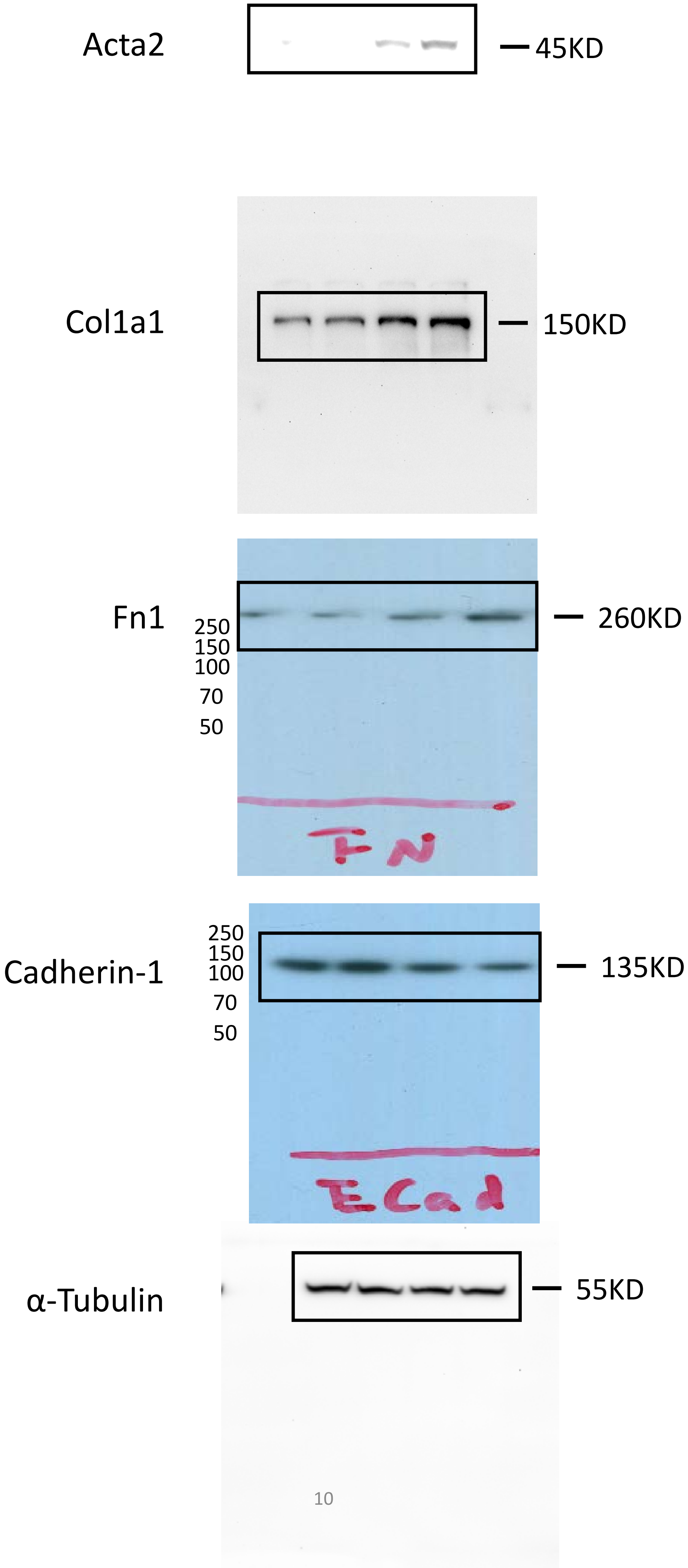
Acta2
45KD
Col1a1
150KD
Fn1
250
150
100
70
50
260KD
Cadherin-1
250
150
100
70
50
135KD
α-Tubulin
55KD

Fig.7E

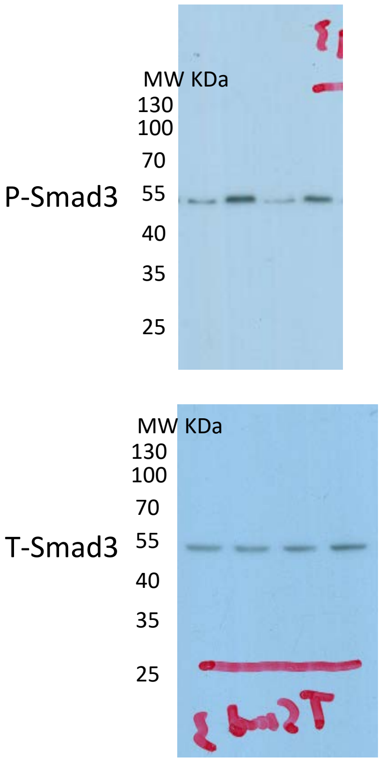
MW KDa
130
100
70
P-Smad3
55
40
35
25
MW KDa
130
100
70
T-Smad3
55
40
35
25

Fig.7F

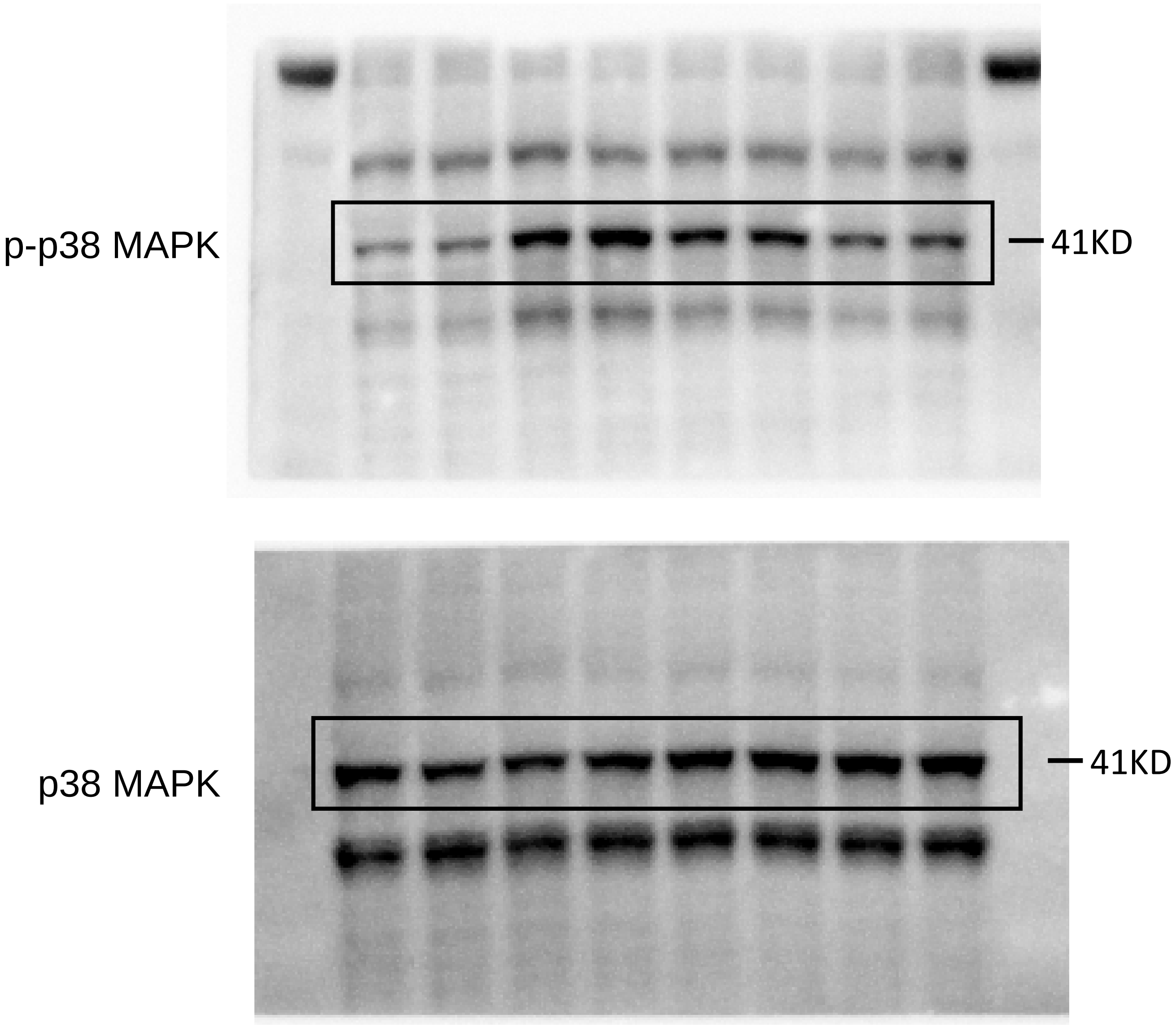

Fig.7F

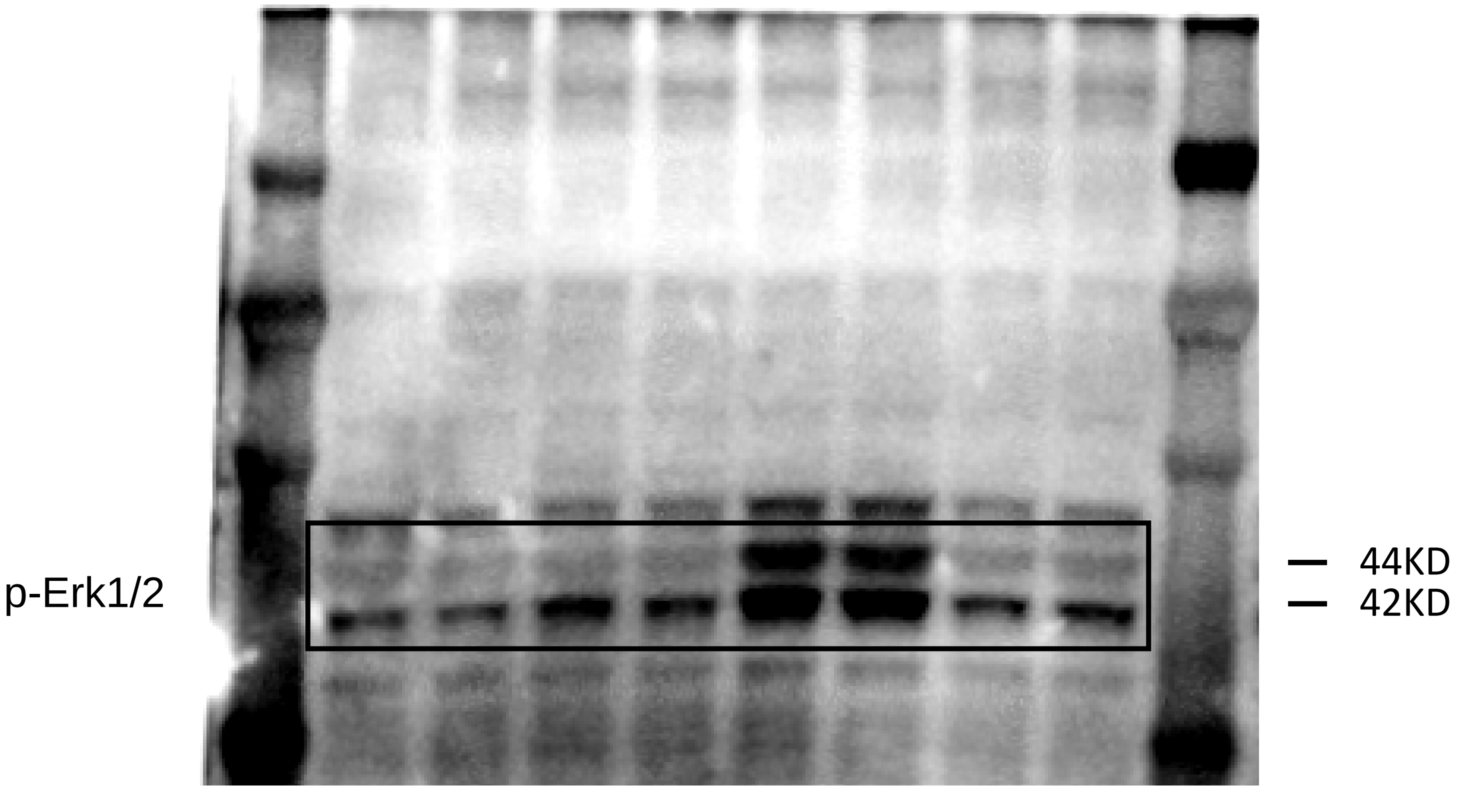

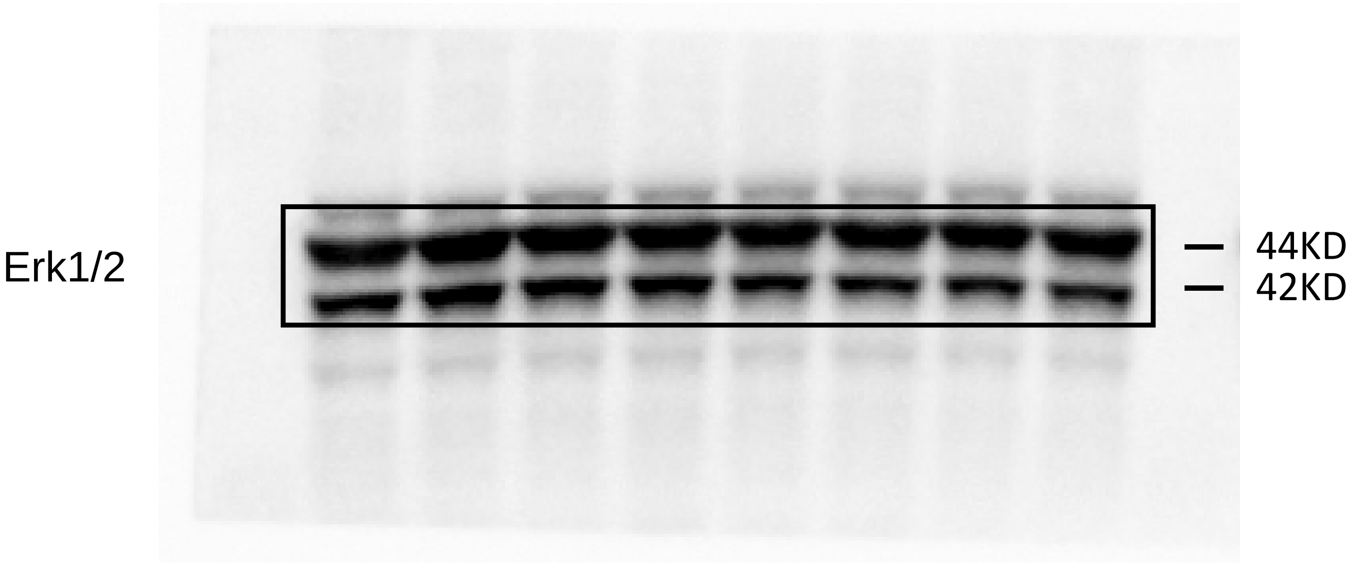

Fig.7F

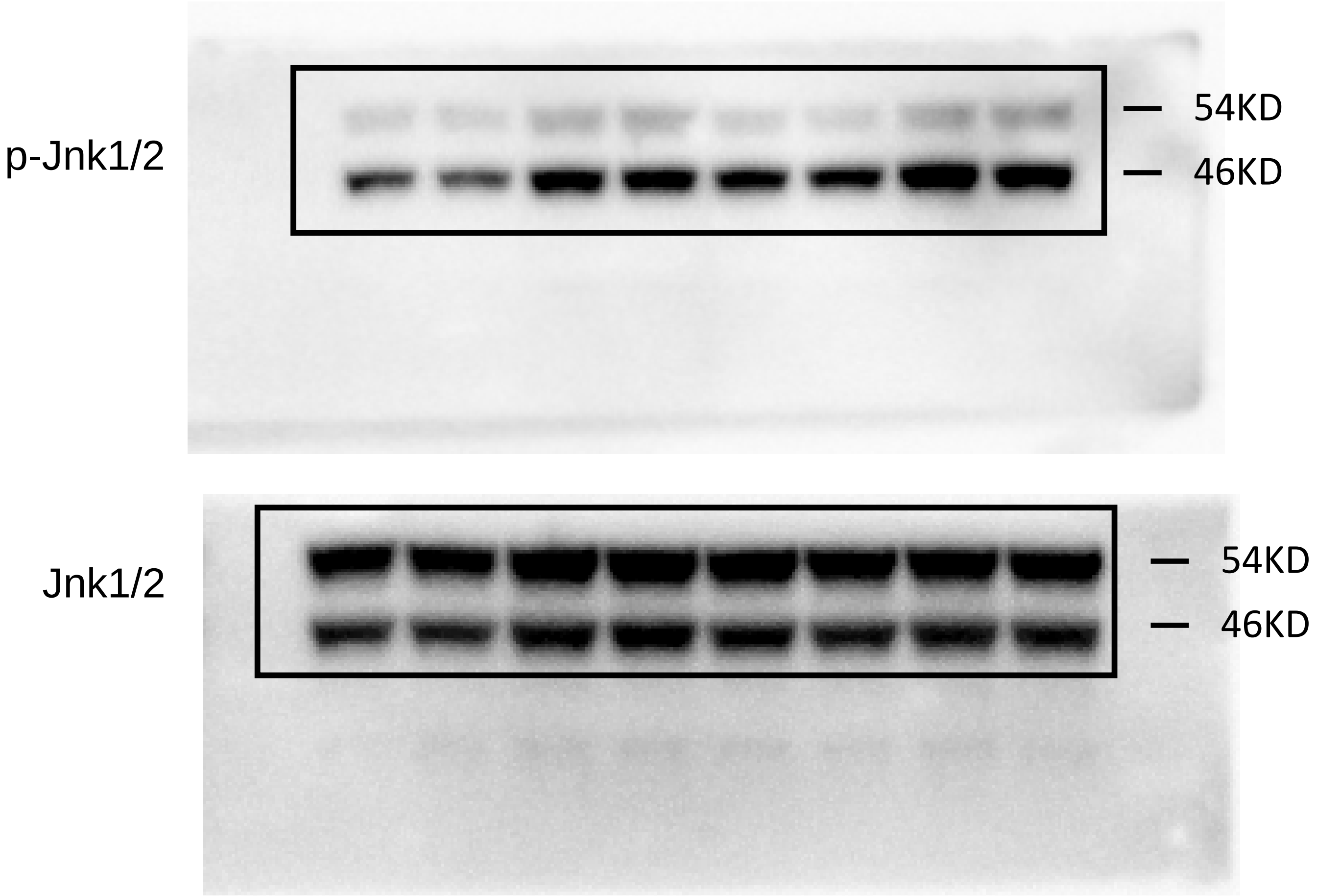

Fig.7G

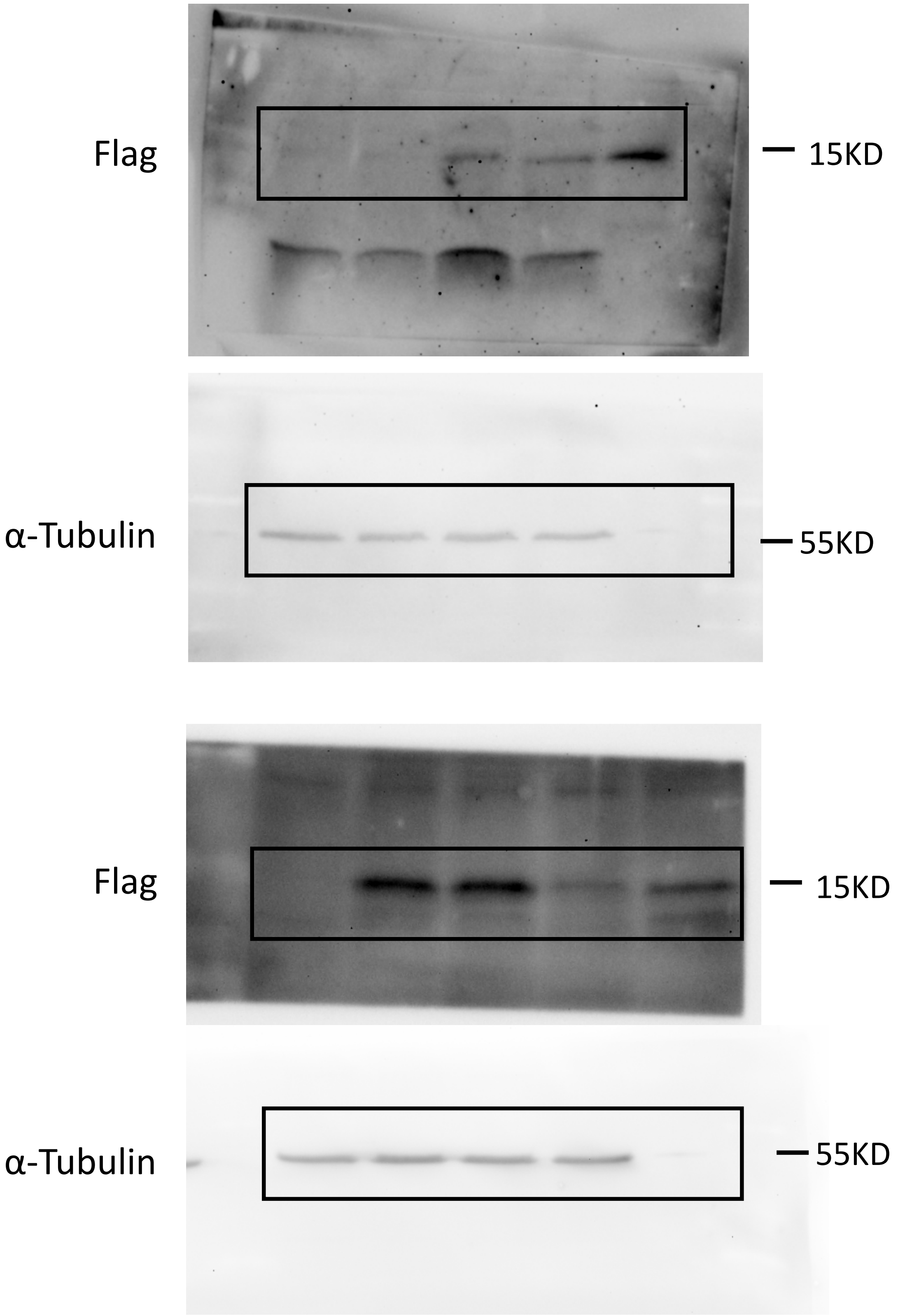

Fig.7G

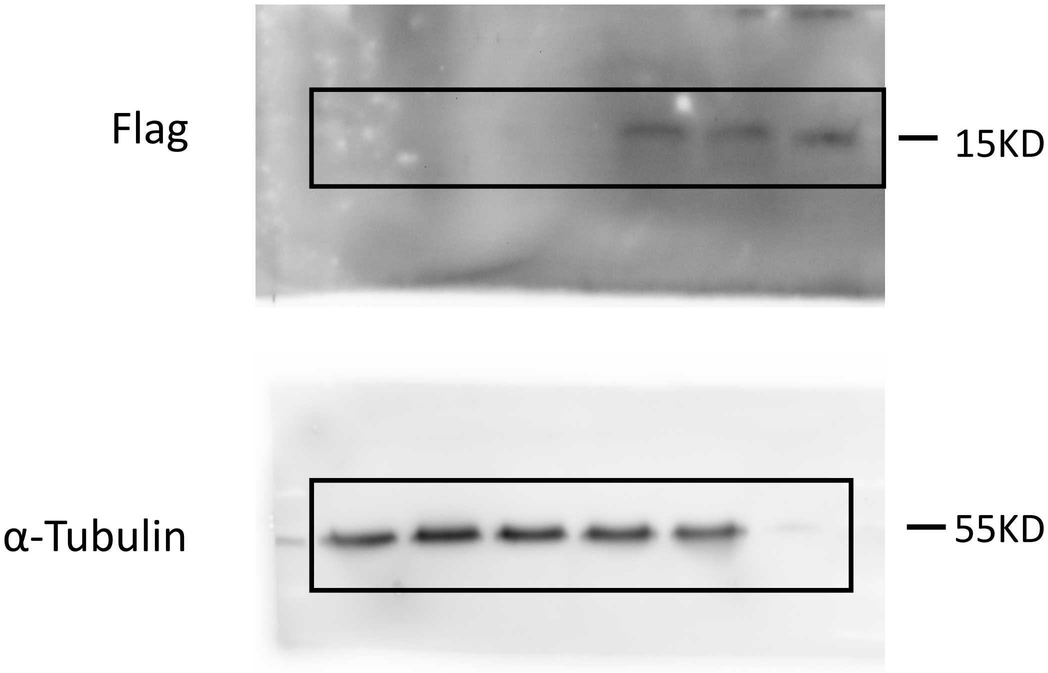

Fig.7K

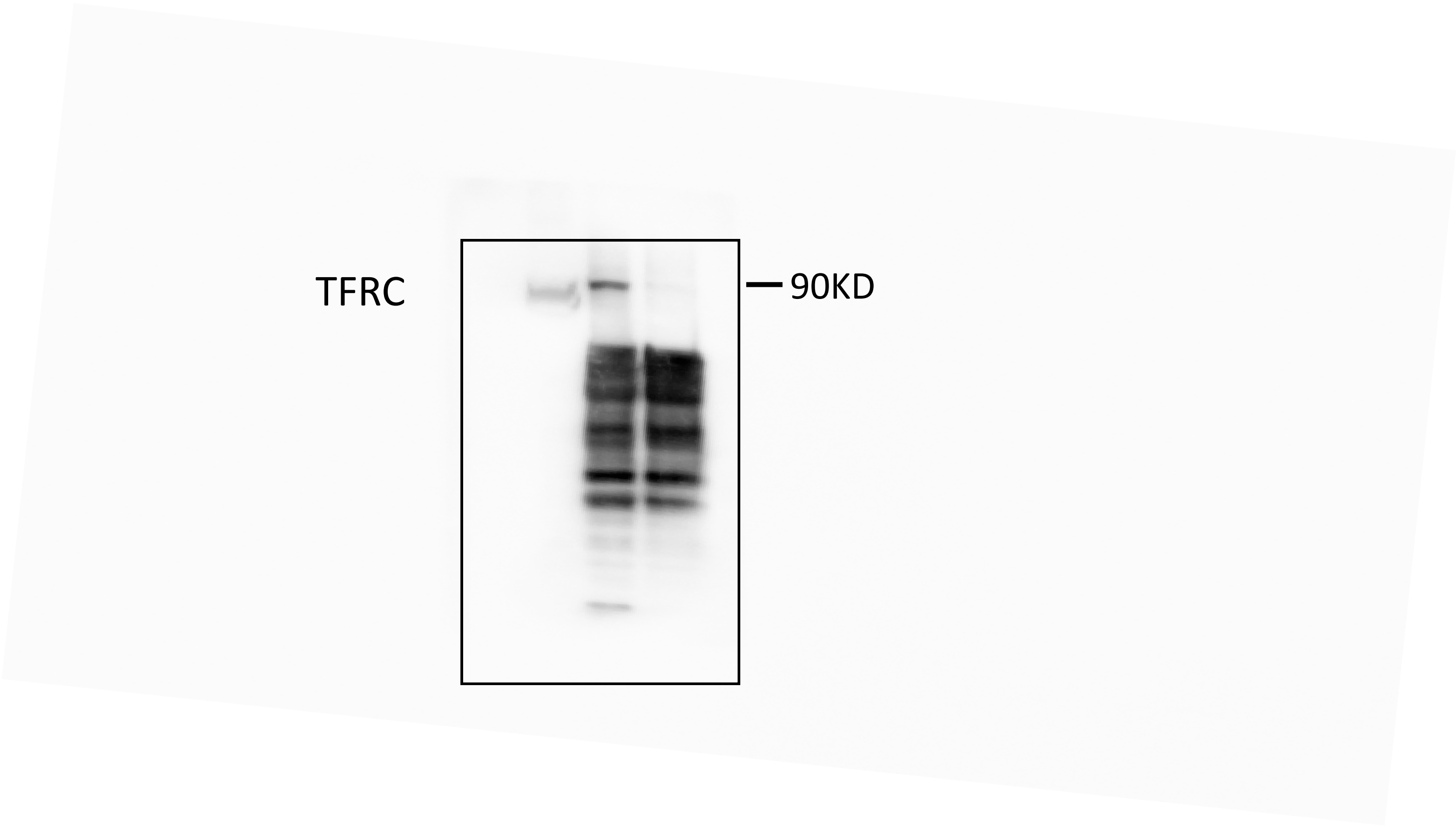

Fig.7M

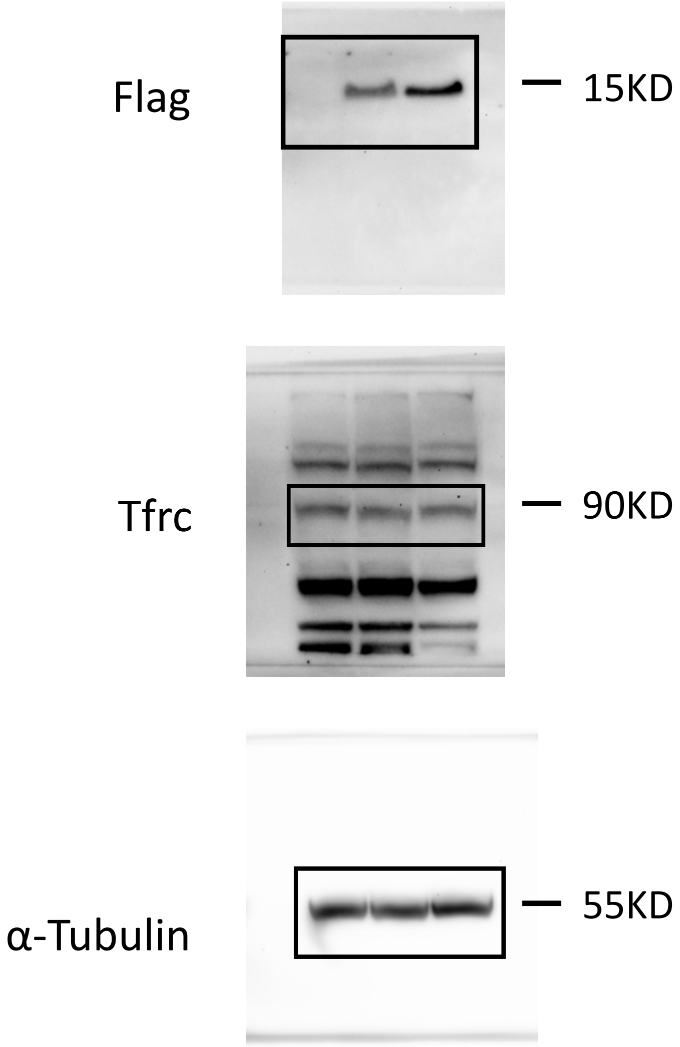

Fig.7N

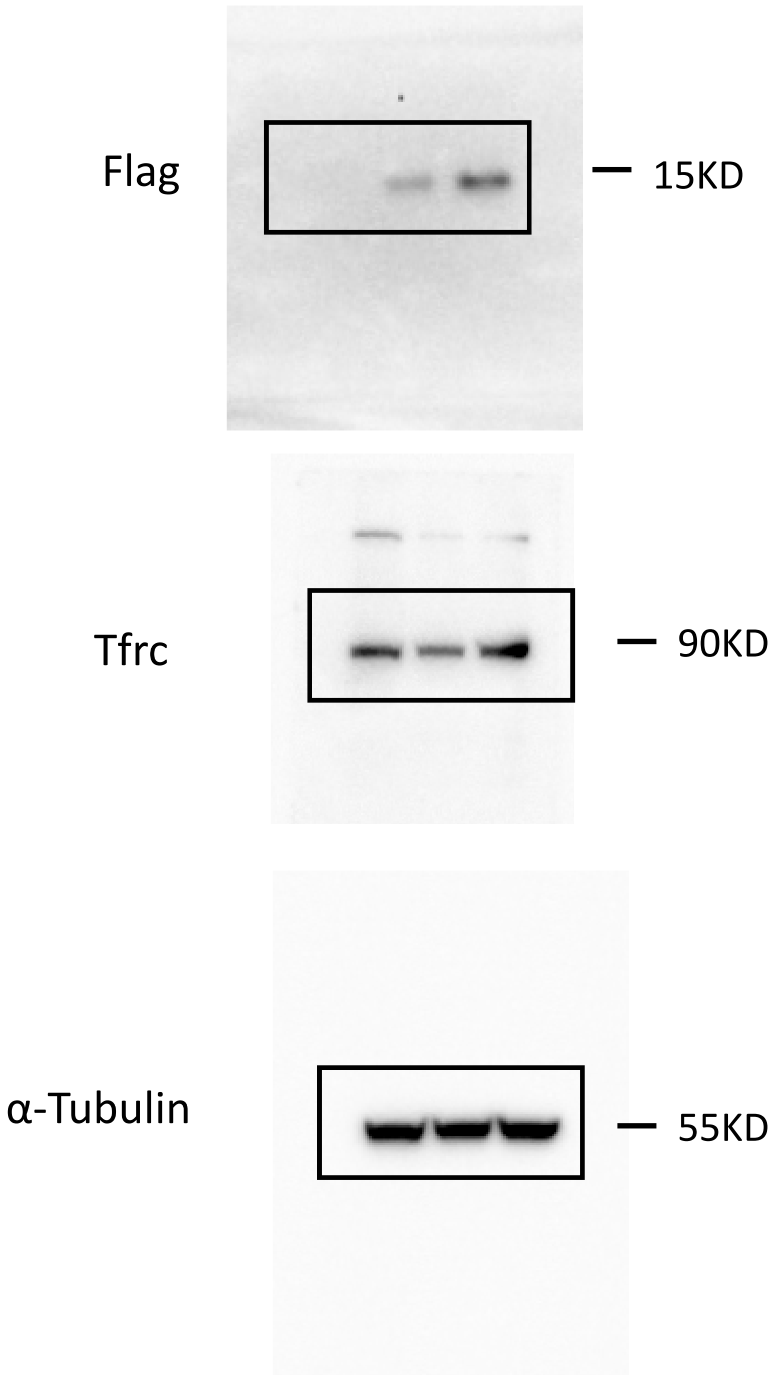

Fig.8C

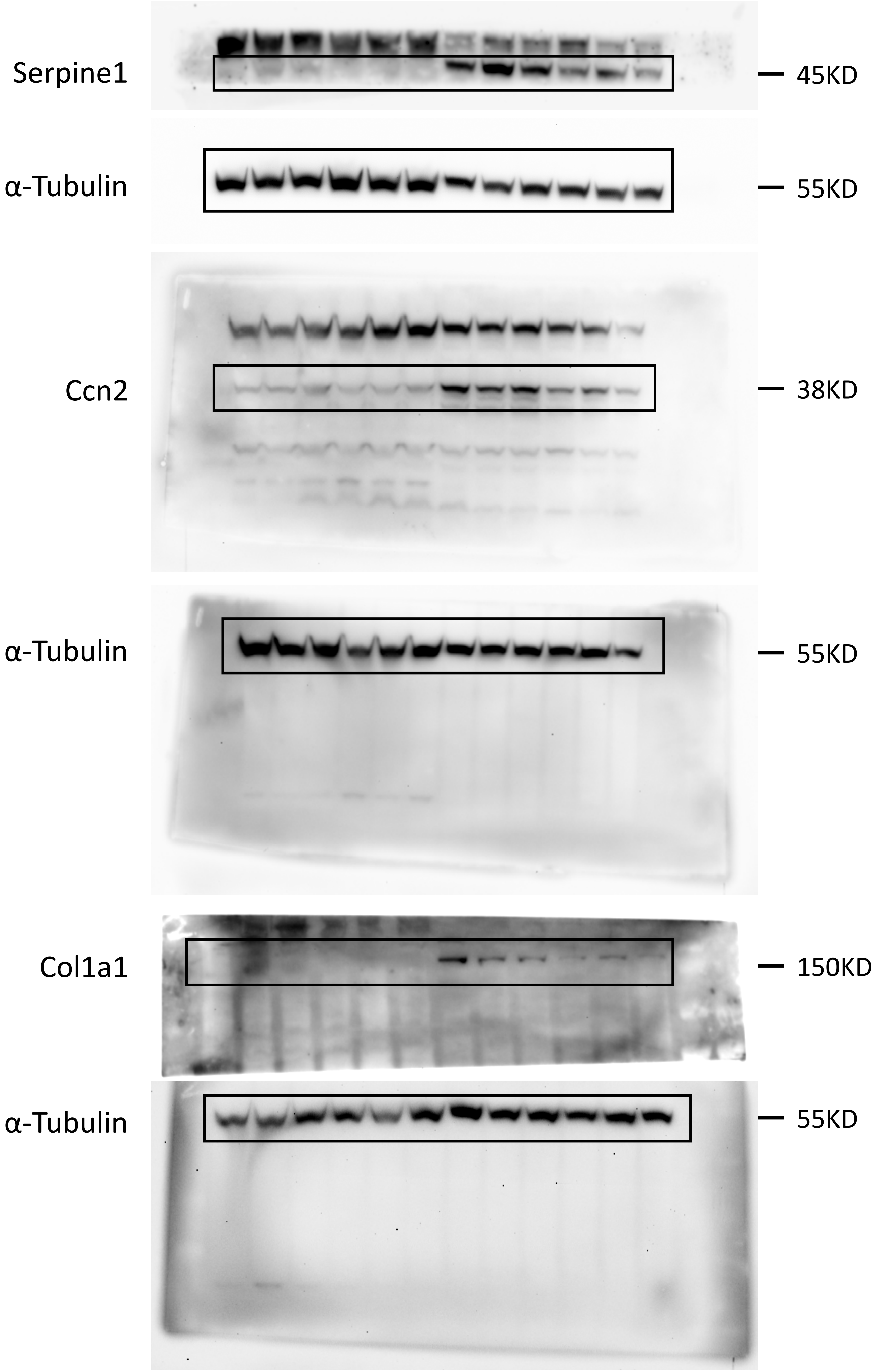
Serpine1
45KD
α-Tubulin
55KD
Ccn2
38KD
α-Tubulin
55KD
Col1a1
150KD
α-Tubulin
55KD

Fig.8D

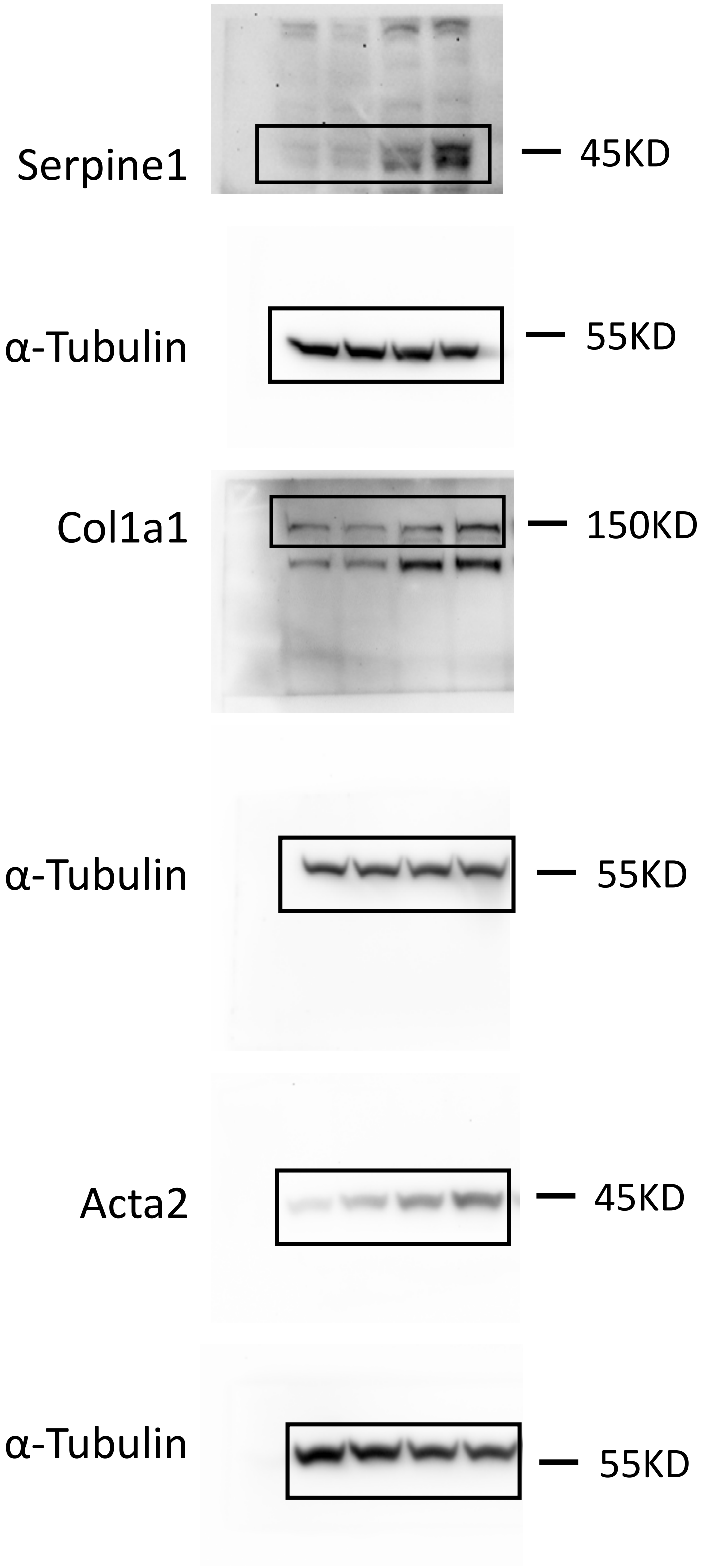

Serpine1
45KD
α-Tubulin
55KD
Col1a1
150KD
α-Tubulin
55KD
Acta2
45KD
α-Tubulin
55KD

Fig.8J

Serpine1

250

100

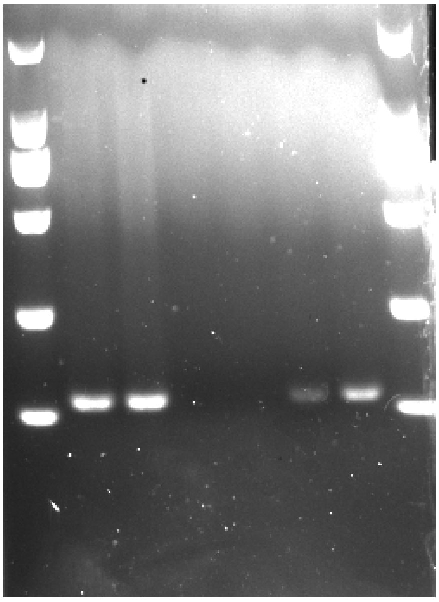

TGF-β1 - + - + - +

input IgG Flag

Ccn2

500

250

100

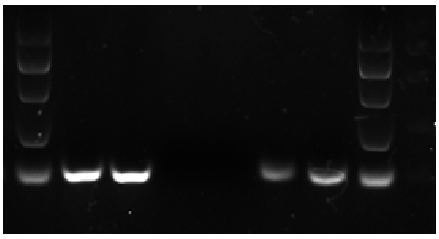

TGF-β1 - + - + - +

input IgG Flag

Supplementary Figure S13A

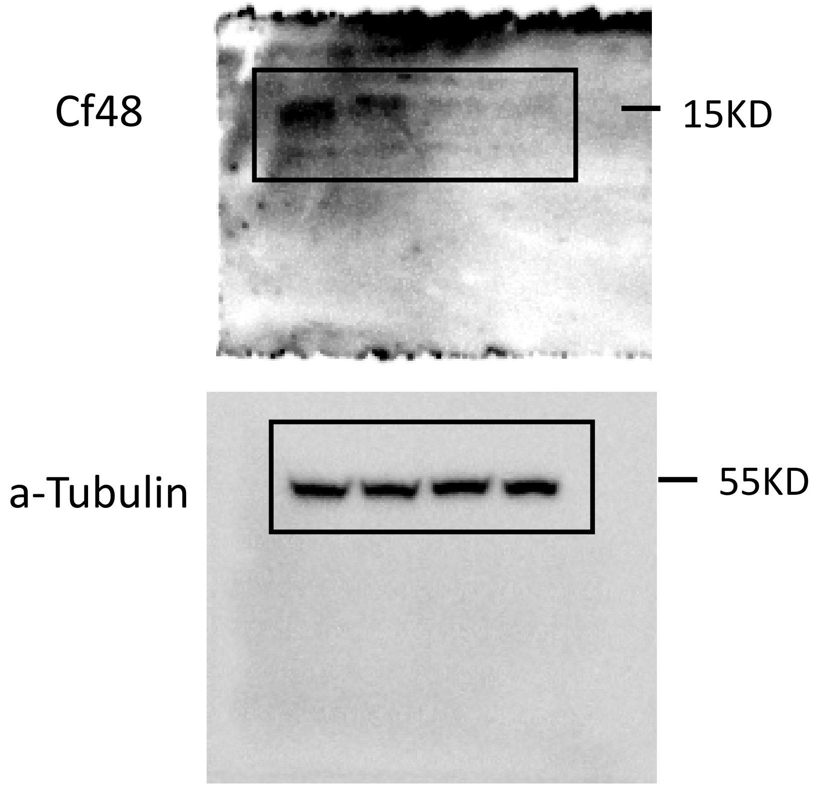

Supplementary Figure S14

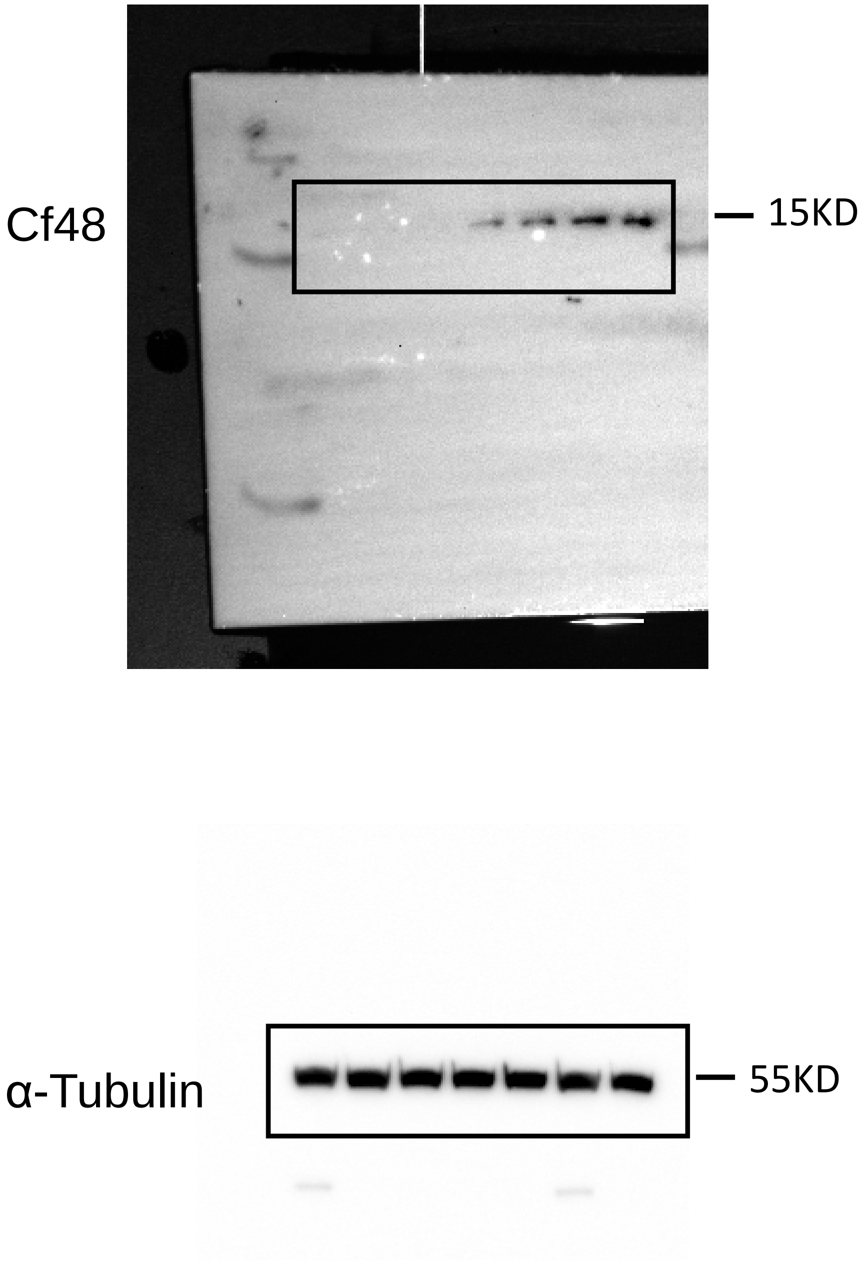

Supplementary Figure S16B

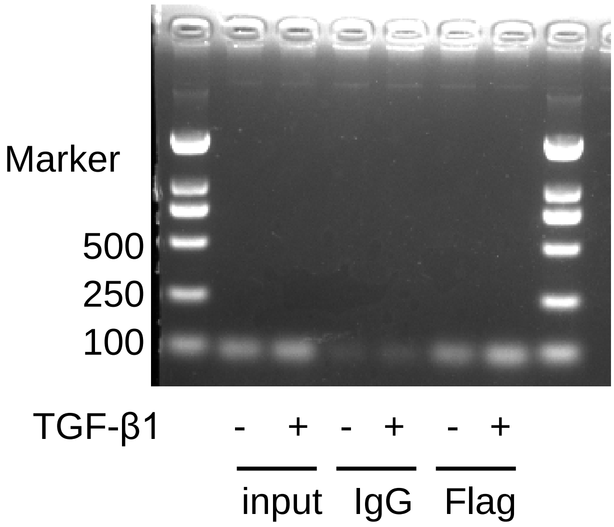

Supplementary Figure S17A

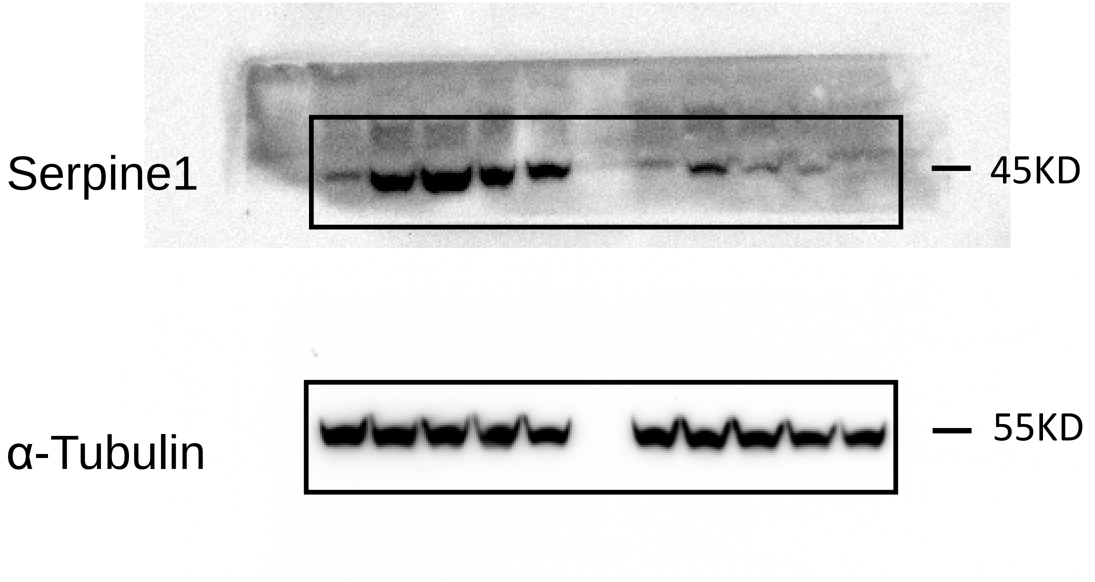

Supplementary Figure S18A

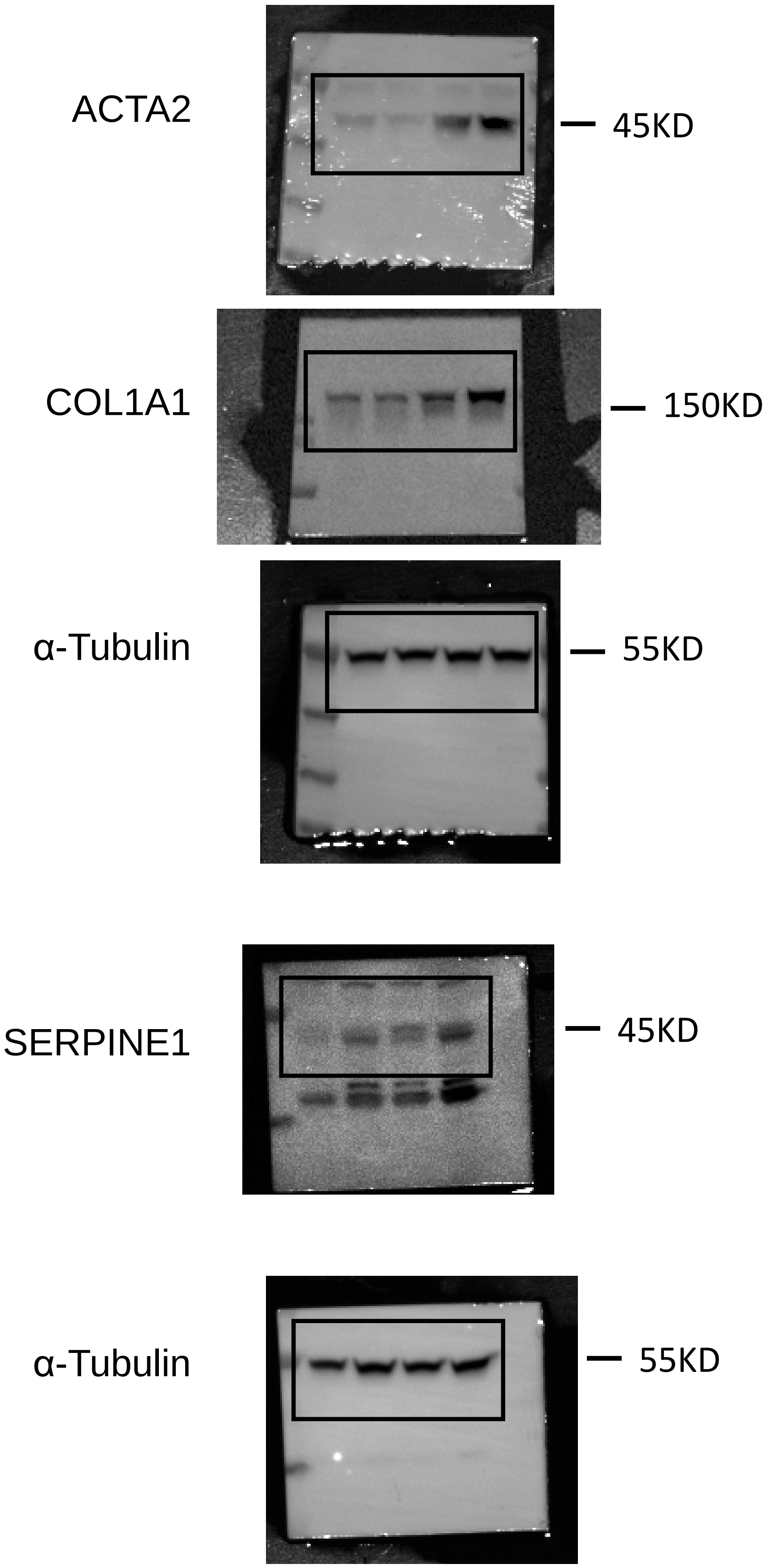

Supplementary Figure S18C

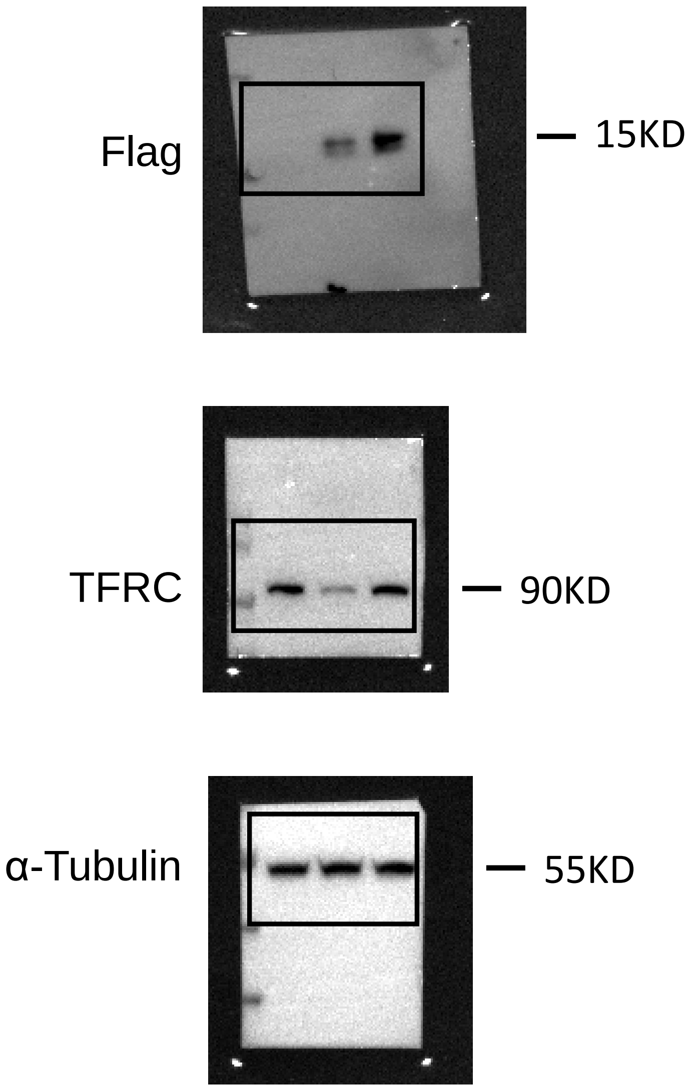

Supplement: Unedited blot and gel images [file jci-134-178392-s176.pdf]
