# Supplementary material for: The secreted micropeptide C4orf48 enhances renal fibrosis via an RNA-binding mechanism
Source: J Clin Invest. 2024 Apr 16;134(10):e178392. doi: 10.1172/JCI178392 (PMC11093611; doi:10.1172/JCI178392)
Supplement: Supplemental table 12 [file jci-134-178392-s180.pdf]

Supplementary Table S12. Recombinant Fc-Flag-Cf48 protein sequence

Sequence
MKHLWFFLLLVAAPRWVLSCPAPELLGGPSVFLFPPKPKDQLMISRTPEVTCVVVDVSHEDPEVKFNWYVDGVEVHNAKTKPREEQYNSTYRVVSVLTVLHQDWLNGKEYKCKVSNKALPAPIEKTISKAKGQPREPQVYTLPPSREEMTKNQVSLTCLVKGFYPSDIAVEWESNGQPENNYKTTPPVLDSDGSFFLYSKLTVDKSRWQQGNVFSCSVLHEALHNHYTQKSLSLSPGKDDDDKSRDYKDDDDK**EPATGSAVPAQSRPCVDCHAFEFMQRALQDLRKTAYSLDARTETLLLQAERRALCACWPAGR*****

Features:

Signal Peptide [1:19]

Fc tag [20:238]

Enterokinase sites [239:243]

Flag tag [244:251]

C4orf48 secreted peptide [252:315]
