# Supplementary material for: The secreted micropeptide C4orf48 enhances renal fibrosis via an RNA-binding mechanism
Source: J Clin Invest. 2024 Apr 16;134(10):e178392. doi: 10.1172/JCI178392 (PMC11093611; doi:10.1172/JCI178392)
Supplement: Supplemental table 13 [file jci-134-178392-s181.pdf]

Supplementary Table S13. cDNA primers used in real-time PCR measurements

	Primer Sequence
Cf48 (mouse)	Forward: 5'-TGT CGC CAC GGA CTT TAC TG-3'<br>Reverse: 5'-TAG GCG GTT TTC CGT AGG TC-3'
Ccl2 (mouse)	Forward: 5'-CCC ACT CAC CTG CTG CTA C-3'<br>Reverse: 5'-TTC TTG GGG TCA GCA CAG A-3'
Tnf (mouse)	Forward: 5'-TCG TAG CAA ACC ACC AAG TG-3'<br>Reverse: 5'-CCT TGA AGA GAA CCT GGG AG-3'
Il1b (mouse)	Forward: 5-ACT GTG AAA TGC CAC CTT TTG-3'<br>Reverse: 5'-TGT TGA TGT GCT GCT GTG AG-3'
Havcr1 (mouse)	Forward: 5'-ACA TAT CGT GGA ATC ACA ACG AC-3'<br>Reverse: 5'-ACT GCT CTT CTG ATA GGT GAC A-3'
Lcn2 (mouse)	Forward: 5'-GCA GGT GGT ACG TTG TGG G-3'<br>Reverse: 5'-CTC TTG TAG CTC ATA GAT GGT GC-3'
Serpine1 (mouse)	Forward: 5'-CCG ATG GGC TCG AGT ATG AC-3'<br>Reverse: 5'-TCC AAG ATG TTG GTG AGG GC-3'
β-Actin (mouse)	Forward: 5'-GAC ATG GAG AAG ATC TGG CA-3'<br>Reverse: 5'-GGT CTT TAC GGA TGT CAA CG-3'
Serpine1 (rat)	Forward: 5'-CTT CTT AGA GGC CAG CAC CC-3'<br>Reverse: 5'-ATG TCG TAC TCG TGC CCA TC-3'
β-Actin (rat)	Forward: 5'-TGC TGA CAG GAT GCA GAA GG-3'<br>Reverse: 5'-AGC CAC CAA TCC ACA CAG AG-3'

Acta2 (rat)	Forward: 5'-AGACCTTCAATGTCCCTGCC -3'<br>Reverse: 5'-GTCCAGCACAATACCAGTTGT-3'
Ccn2 (rat)	Forward: 5'-GCGTGTGCACTGCCAAAGAT-3'<br>Reverse: 5'-CAGGCAAGTGCACTGGTATTTG-3'
SERPINE1 (human)	Forward: 5'- ACC GCA ACG TGG TTT TCT CA -3'<br>Reverse: 5'- TTG AAT CCC ATA GCT GCT TGA AT -3'
CCN2 (human)	Forward: 5'- CAG CAT GGA CGT TCG TCT G -3'<br>Reverse: 5'- AAC CAC GGT TTG GTC CTT GG -3'
TFRC (human)	Forward: 5'- GGC TAC TTG GGC TAT TGT AAA GG -3'<br>Reverse: 5'- CAG TTT CTC CGA CAA CTT TCT CT -3'
β-Actin (human)	Forward: 5'- CAT GTA CGT TGC TAT CCA GGC -3'<br>Reverse: 5'- CTC CTT AAT GTC ACG CAC GAT -3'
