# Supplementary material for: The secreted micropeptide C4orf48 enhances renal fibrosis via an RNA-binding mechanism
Source: J Clin Invest. 2024 Apr 16;134(10):e178392. doi: 10.1172/JCI178392 (PMC11093611; doi:10.1172/JCI178392)
Supplement: Supplemental table 14 [file jci-134-178392-s182.pdf]

Supplementary Table S14. Rat Tfrc and human TFRC siRNA and NC siRNA sequences

rat-Tfrc siRNA:
sense: GCUACUUCUAGACUAACAACUtt
antisense: AGUUGUUAGUCUAGAAGUAGCtt

rat-Tfrc scramble siRNA (NC siRNA):
sense: GUCGAUCUCUGCCCAUAGGUUtt
antisense: AACCUAUGGGCAGAGAUCGACtt

human-TFRC siRNA:
sense: GGAUCUAUAGUGAUUGUCAtt
antisense: UGACAAUCACUAUAGAUCCtt

human-TFRC scramble siRNA (NC siRNA):
sense: GGGUUAUGCGUUAAACUUAtt
antisense: UAAGUUUAACGCAUAACCCtt
