# Supplementary material for: The secreted micropeptide C4orf48 enhances renal fibrosis via an RNA-binding mechanism
Source: J Clin Invest. 2024 Apr 16;134(10):e178392. doi: 10.1172/JCI178392 (PMC11093611; doi:10.1172/JCI178392)
Supplement: Supplemental table 4 [file jci-134-178392-s185.pdf]

Supplementary Table S4. Characteristics of CKD patients among groups

Variable	DKD	IgAN	LN	*P* value
No. of patients	33	20	20	-
Cf48 concentration (ng/ml)	9.68±4.66	7.13±2.71	9.10±4.90	0.113
Age (year)	51.76±9.62	35.10±10.42	35.65±11.88	<0.0001
Male (%)	30 (90.9%)	10 (50.0%)	5 (25.0%)	-
Creatinine (μmol/l)	301.42±228.66	230.15±221.62	158.05±127.22	0.056
BUN (mmol/l)	14.47±8.05	9.65±5.93	11.10±8.10	0.065
Serum albumin (g/l)	34.24±6.36	36.31±3.77	30.51±8.49	0.022
24h Urinary protein (g/24h)	5.80±3.63	1.81±1.15	3.01±3.68	<0.0001
eGFR (ml/min/1.73m^2)	40.25±31.61	49.62±30.10	58.58±36.34	0.141
CKD Stage (%)				-
1-2	8 (24.2%)	8 (40.0%)	9 (45.0%)	-
3	7 (21.2%)	7 (35.0%)	6 (30.0%)	-
4	7 (21.2%)	1 (5.0%)	1 (5.0%)	-
5	11 (33.4%)	4 (20.0%)	4 (20.0%)	-

Values are shown as means ± SDs. Abbreviations: DKD= diabetic kidney disease; LN= lupus nephritis; Cf48= C4orf48; BUN= blood urea nitrogen; eGFR= estimated glomerular filtration rate.

Sequence
MKHLWFFLLLVAAPRWVLSCPAPELLGGPSVFLFPPKPKDQLMISRTPEVTCVVVDVSHEDPEVKFNWYVDGVEVHNAKTKPREEQYNSTYRVVS<br>VLTVLHQDWLNGKEYKCKVSNKALPAPIEKTISKAKGQPREPQVYTLPPSREEMTKNQVSLTCLVKGFYPSDIAVEWESNGQPENNYKTTPPVLD<br>SDGSFFLYSKLTVDKSRWQQGNVFSCSVLHEALHNHYTQKSLSLSPGKDDDDKSRDYKDDDDK**EPATGSAVPAQSRPCVDCHAFEFMQRALQDLR**<br>**KTAYSLDARTETLLLQAERRALCACWPAGR***

Supplementary table 2. Recombinant Fc-Flag-Cf48 protein sequence

Features:

Signal Peptide [1:19]

Fc tag [20:238]
Enterokinase sites [239:243]
Flag tag [244:251]
C4orf48 secreted peptide [252:315]

Supplementary table 3. cDNA primers used in real-time PCR measurements

	Primer Sequence
C4orf48	Forward: 5'-TGT CGC CAC GGA CTT TAC TG-3'
	Reverse: 5'-TAG GCG GTT TTC CGT AGG TC-3'
MCP-1	Forward: 5'-CCC ACT CAC CTG CTG CTA C-3'
	Reverse: 5'-TTC TTG GGG TCA GCA CAG A-3'
TNF-	Forward: 5'-TCG TAG CAA ACC ACC AAG TG-3'
	Reverse: 5'-CCT TGA AGA GAA CCT GGG AG-3'
Il-1	Forward: 5-ACT GTG AAA TGC CAC CTT TTG-3'
	Reverse: 5'-TGT TGA TGT GCT GCT GTG AG-3'
Kim1	Forward: 5'-ACA TAT CGT GGA ATC ACA ACG AC-3'
	Reverse: 5'-ACT GCT CTT CTG ATA GGT GAC A-3'
NGAL	Forward: 5'-GCA GGT GGT ACG TTG TGG G-3'
	Reverse: 5'-CTC TTG TAG CTC ATA GAT GGT GC-3'
PAI-1	Forward: 5'-CCG ATG GGC TCG AGT ATG AC-3'
	Reverse: 5'-TCC AAG ATG TTG GTG AGG GC-3'
-Actin	Forward: 5'-GAC ATG GAG AAG ATC TGG CA-3'
	Reverse: 5'-GGT CTT TAC GGA TGT CAA CG-3'
PAI-1 (rat)	Forward: 5'-CTT CTT AGA GGC CAG CAC CC-3'
	Reverse: 5'-ATG TCG TAC TCG TGC CCA TC-3'
-Actin (rat)	Forward: 5'-TGC TGA CAG GAT GCA GAA GG-3'
	Reverse: 5'-AGC CAC CAA TCC ACA CAG AG-3'
