# Supplementary material for: The secreted micropeptide C4orf48 enhances renal fibrosis via an RNA-binding mechanism
Source: J Clin Invest. 2024 Apr 16;134(10):e178392. doi: 10.1172/JCI178392 (PMC11093611; doi:10.1172/JCI178392)
Supplement: Supplemental table 5 [file jci-134-178392-s186.pdf]

Supplementary Table S5. Multiple linear regression analysis of the risk factors for eGFR in CKD patients.

Variables	Standardized Coefficients Beta	95%CI	t	P-value
C4orf48(ng/mL)	-0.484	-5.259, -2.058	-4.562	<0.0001
Sex	0.046	-14.123, 20.269	0.357	0.722
Age(year)	0.098	-0.381, 0.872	0.782	0.437
Renal disease	0.220	-2.885, 20.170	1.496	0.139
